# Conserved and species-specific molecular denominators in mammalian skeletal muscle aging

**DOI:** 10.1038/s41514-017-0009-8

**Published:** 2017-05-05

**Authors:** Evi M. Mercken, Miriam Capri, Bethany A. Carboneau, Maria Conte, Juliana Heidler, Aurelia Santoro, Alejandro Martin-Montalvo, Marta Gonzalez-Freire, Husam Khraiwesh, José A. González-Reyes, Ruin Moaddel, Yongqing Zhang, Kevin G. Becker, José M. Villalba, Julie A. Mattison, Ilka Wittig, Claudio Franceschi, Rafael de Cabo

**Affiliations:** 1 Translational Gerontology Branch, National Institute on Aging, National Institutes of Health, 251 Bayview Boulevard, Baltimore, MD 21224 USA; 2 Department of Experimental, Diagnostic and Specialty Medicine (DIMES), University of Bologna, 40126 Bologna, Italy; 3 Interdepartmental Centre “L. Galvani” (CIG), University of Bologna, 40126 Bologna, Italy; 4 Functional Proteomics, SFB815 Core Unit, Cluster of Excellence Frankfurt “Macromolecular Complexes,” Goethe-University, Theodor-Stern-Kai 7, 60590 Frankfurt am Main, Germany; 5 Departamento de Biología Celular, Fisiología e Inmunología, Universidad de Córdoba, Campus de Excelencia Internacional Agroalimentario ceiA3, Campus Rabanales Edificio Severo Ochoa, 3ª planta, 14014 Córdoba, Spain; 6 Bioanalytical and Drug Development Unit, National institute on Aging, National Institutes of Health, 251 Bayview Boulevard, Baltimore, MD 21224 USA; 7 Gene Expression and Genomics Unit, National Institute on Aging, National Institutes of Health, 251 Bayview Boulevard, Baltimore, MD 21224 USA; 8 Translational Gerontology Branch, National Institute on Aging, Intramural Research Program, Poolesville, MD 20837 USA; 9 German Center of Cardiovascular Research (DZHK), Partner site RheinMain, Frankfurt, Germany; 10 IRCCS, Institute of Neurological Sciences of Bologna, 40139 Bologna, Italy

## Abstract

Aging is a complex phenomenon involving functional decline in multiple physiological systems. We undertook a comparative analysis of skeletal muscle from four different species, i.e. mice, rats, rhesus monkeys, and humans, at three different representative stages during their lifespan (young, middle, and old) to identify pathways that modulate function and healthspan. Gene expression profiling and computational analysis revealed that pathway complexity increases from mice to humans, and as mammals age, there is predominantly an upregulation of pathways in all species. Two downregulated pathways, the electron transport chain and oxidative phosphorylation, were common among all four species in response to aging. Quantitative PCR, biochemical analysis, mitochondrial DNA measurements, and electron microscopy revealed a conserved age-dependent decrease in mitochondrial content, and a reduction in oxidative phosphorylation complexes in monkeys and humans. Western blot analysis of key proteins in mitochondrial biogenesis discovered that (i) an imbalance toward mitochondrial fusion occurs in aged skeletal muscle and (ii) mitophagy is not overtly affected, presumably leading to the observed accumulation of abnormally large, damaged mitochondria with age. Select transcript expression analysis uncovered that the skeletal inflammatory profile differentially increases with age, but is most pronounced in humans, while increased oxidative stress (as assessed by protein carbonyl adducts and 4-hydroxynonenal) is common among all species. Expression studies also found that there is unique dysregulation of the nutrient sensing pathways among the different species with age. The identification of conserved pathways indicates common molecular mechanisms intrinsic to health and lifespan, whereas the recognition of species-specific pathways emphasizes the importance of human studies for devising optimal therapeutic modalities to slow the aging process.

## Conserved and unique aging regulatory pathways

Aging is a complex phenomenon involving functional declines in multiple physiological systems with the passage of time. Focusing on skeletal muscle, a group of international scientists identified pathways involved in healthspan and by determining global gene expression profiles across species they exposed common mechanisms fundamental to the aging process. Their experimental design involved comparative analysis of mice, rats, rhesus monkeys and humans, targeting three key time points during their respective lifespans. Pathways related to oxidative stress, inflammation and nutrient signaling, which function collectively to affect the quality and status of mitochondria, emerged across all species in an age-influenced manner. The identification of conserved pathways reveals molecular mechanisms intrinsic to health and survival, whereas the unveiling of species-specific pathways emphasizes the importance of human studies for devising optimal therapeutic modalities to slow the aging process.

## Introduction

Aging is a complex and inevitable phenomenon that almost certainly involves the gradual accumulation of damage to the cellular machinery, eventually leading to the progressive deterioration of normal biological functions, and an increased risk of disease and death.^1^ One of the hallmarks of aging is a marked decline in human skeletal muscle mass and strength, commonly referred to as sarcopenia.^2^ Indeed, sarcopenia has been observed across a wide variety of species, including worms, flies, rodents and primates.^3–5^ Sarcopenia is a multifactorial condition involving many potential mechanisms, such as an altered hormonal status (decreased levels of testosterone, androgen), inefficient protein synthesis, satellite cell dysfunction, muscle denervation, and altered neuromuscular junction integrity. Some of the key processes thought to muscle loss include mitochondrial dysfunction, oxidative stress, chronic inflammation, and perturbations to the insulin-like-growth factor (IGF-1) and the mechanistic target of rapamycin (mTOR) signaling pathways.^6, 7^

Mitochondria are considered the powerhouse of the cell, and use oxygen to generate energy in the form of ATP, creating reactive oxygen species (ROS) as by-products in this process. Importantly, these organelles are highly dynamic, exhibiting the ability to carry out fission and fusion.^8^ The former allows mitochondria to transmit among dividing cells, meet increased ATP demands, and maintain mitochondrial quality, primarily through autophagy.^9, 10^ Fusion is the counterpart biological event, and involves the mixture of mitochondrial compartments to (i) preserve the equilibrium of oxidative phosphorylation (OXPHOS) proteins critical to respiration, (ii) carry out energy exchange, and (iii) complement defects in the mitochondrial genome.^11, 12^ Additionally, it has been uncovered that mitochondrial electron transport chain (ETC) components can exist as supercomplexes, facilitating the electron flow, and hence making the system more efficient.^13, 14^ Destabilization of the supramolecular organization may contribute to the development of an aging phenotype, as it has been shown that a lack of supercomplex formation leads to mitochondrial activity impairment, and reduced ATP levels.^15^

In many studies, age-dependent mitochondrial dysfunction has been reported to drive life-threatening disorders, such as frailty, and accelerate the aging process.^16, 17^ Recent reviews by Nair and others^18–20^ have highlighted the specific involvement in human sarcopenia of reduced mitochondrial DNA (mtDNA) and protein content, impaired enzymatic activity and elevated markers of oxidative damage. However, a report by Lustgarten et al.^21^ found that oxidative stress, introduced by deletion of the radical scavenger MnSOD in mice, resulted in mitochondrial impairment, but not muscle atrophy during aging. Thus, the molecular choreography between mitochondria and aging is multi-factorial and requires further investigation.

Often associated with defects in mitochondrial function is oxidative stress. Elevated levels of ROS can lead to increased macromolecular damage, namely to lipids, proteins and nucleic acids, which in turn can promote cellular dysfunction and organismal aging. Although the free radical theory of aging has experienced its setbacks, there is still significant evidence indicating an age-dependent accumulation of oxidative modifications in the main cellular components in skeletal muscle.^22^ Nevertheless, a revised look at this theory has suggested that harmful imbalances in redox homeostasis or signaling may be more central to the aging phenomenon than to the oxidative damage itself.^23^

Another major etiological event in aging muscle is low-grade chronic inflammation, or the so-called inflamm-aging phenomenon.^24^ The inflammatory response is also a common denominator of major diseases such as diabetes, obesity and cancer.^25^ This event has been proposed to arise due to an overall condition of oxidative stress, which activates signaling pathways, such as the nuclear factor-κB-driven (NF-κB) transcriptional response. The resulting production of pro-inflammatory cytokines and ROS further augment this perturbed oxidative redox state, leading to a vicious cycle.

The nutrient-sensing insulin/IGF-1/mTOR pathway is a highly conserved pathway in evolution that is involved in the regulation of cell growth, survival, and energy metabolism.^26^ The kinase Akt plays an important role in this pathway, as Akt stimulates (i) protein synthesis by activating the kinase mTOR and glycogen synthase kinase 3β (GSK-3β) and (ii) protein degradation by repressing the transcription factor FoxO.^7, 27^ This insulin/IGF-1/mTOR network also plays a complex role in the aging process. In the case of muscle growth, the pathway is activated, whereas reduced signaling has been associated with longevity, both in humans and model organisms.^28^

While the above processes all have supporting evidence for their involvement in the aging phenomenon, there are also reports that have raised questions about their participation in this process. To attempt to consolidate the current picture regarding skeletal muscle aging, we have analyzed four distinct species with vastly different lifespans, i.e., humans, monkeys, rats and mice, to identify genetic pathways that are commonly regulated. Conserved pathways are of specific interest because they may reveal molecular mechanisms intrinsic to aging, which ultimately play a major role in modulating human health and lifespan. A particular strength of this study is the inclusion of multiple age-groups that span the comparative life stages across species, and the involvement of an advanced-age group, which allows for the detection of gradually changing molecular events and those that occur strictly later in life.

## Results

### Age-related differences in body composition among species

Aging has been associated with alterations in body composition in various species, including humans, and such changes can promote adverse health outcomes and retard successful aging. We determined the effects of aging on bodyweight and body composition across four different species—C57Bl/6J male mice, Fischer 344 male rats, male rhesus monkeys, and male humans—at three different ages chosen to represent similar stages during their respective lifespans (see below), broadly defined as young, middle-aged and old (Table 1). All species were heavier at middle and old age compared to their young counterparts, although no significant difference in bodyweight was observed for humans between the middle and old age classifications (Table 1). We note that in humans one individual was an outlier for these parameters, and excluding that participant from the analysis indicates a statistically significant difference, similar to what is seen for mice, rats and monkeys, i.e., higher weight at middle and old age relative to young.

**Table 1:** Study characteristics

		Mice	Rats	Rhesus	Humans
Young	*n*	5	5	6	5
	Age (m/y)	5 month	5 month	6.00 ± 0.00 years	31.2 ± 0.86 years
	Height, cm	–	–	–	173.4 ± 4.0
	BW (g/kg)	29.93 ± 0.85	243.1 ± 8.2	8.50 ± 0.21	78.40 ± 7.28
	BMI (kg/m^2^)	–	–	–	25.86 ± 2.23
	Fat mass (%)	19.61 ± 0.93	25.00 ± 0.22	20.13 ± 4.65	–
	Lean mass (%)	59.44 ± 0.39	65.70 ± 0.21	79.87 ± 4.64	–
	MT (cm)	–	–	–	1.91 ± 0.08
Middle	*n*	5	5	6	4
	Age (m/y)	17 months	17 months	16.48 ± 0.31 years	46.0 ± 0.71 years
	Height (cm)	–	–	–	176.2 ± 2.2
	BW (g/kg)	41.67 ± 2.11^*^	401.0 ± 14.6^*^	13.31 ± 0.33^*^	87.50 ± 7.15
	BMI (kg/m^2^)	–	–	–	28.28 ± 1.91
	Fat mass (%)	24.30 ± 0.57^*^	30.68 ± 0.31^*^	21.07 ± 6.68	–
	Lean mass (%)	58.25 ± 0.17^*^	61.04 ± 0.33^*^	78.92 ± 6.68	–
	MT (cm)	–	–	–	1.71 ± 0.09
Old	*n*	5	5	6	7
	Age (m/y)	28 months	30 months	26.83 ± 0.22 years	74.0 ± 4.38 years
	Height (cm)	–	–	–	169.9 ± 2.2
	BW (g/kg)	46.55 ± 7.30^*^	417.1 ± 15.7^*^	11.12 ± 0.68^*^	85.86 ± 3.16
	BMI (kg/m^2^)	–	–	–	29.74 ± 0.99
	Fat mass (%)	13.76 ± 0.36^*^	30.41 ± 0.31^*^	19.43 ± 5.23	–
	Lean mass (%)	60.91 ± 0.18^*^	61.19 ± 0.32^*^	70.32 ± 11.4	–
	MT (cm)	–	–	–	1.35 ± 0.13^*^

Values are mean ± SEM. Fat mass and lean mass data from mice (young: *n* = 20, middle: *n* = 15, old: *n* = 11), rats (young: *n* = 29, middle: *n* = 27, old: *n* = 21), monkeys (young: *n* = 6, middle: *n* = 6, old: *n* = 3)

*m*/*y* months/years, *BW* body weight, *BMI* body mass index, *MT* muscle thickness

*p < 0.05 compared to young

Contrary to our expectations, middle-aged mice had higher percentage fat mass and lower percentage muscle mass compared to young mice, whereas old mice exhibited lower percentage fat mass and higher percentage lean mass (Table 1). However, when lean mass was not normalized to bodyweight, a significant decrease in lean mass was observed in old mice compared to young (20.3 g ± 1.23 young vs. 18.94 g ± 0.83 old). In agreement with these data, wet mass of hindlimb muscles (soleus, plantaris, gastrocnemius, tibialis, and extensor digitorumlongus) were lighter in old mice compared to young (Fig. S1). In rats, our data indicate that fat mass increases with age, while lean mass decreases. A similar pattern is observed for rhesus monkeys, comparing middle and old aged animals, and for humans, as measured by muscle thickness (Table 1). Consistent with previous studies,^16, 29–31^ our results show that muscle mass among all species declines with age (Table 1). Most striking was the fat mass distribution between mice as compared to rats, rhesus monkeys, and humans; old mice have lower fat mass compared to their younger counterparts (Table 1). Therefore, in terms of body composition, humans seem to be most closely related to their nearest evolutionary counterparts.

### Common signature across species indicative of disruptive mitochondrial homeostasis

**Fig. 1:**
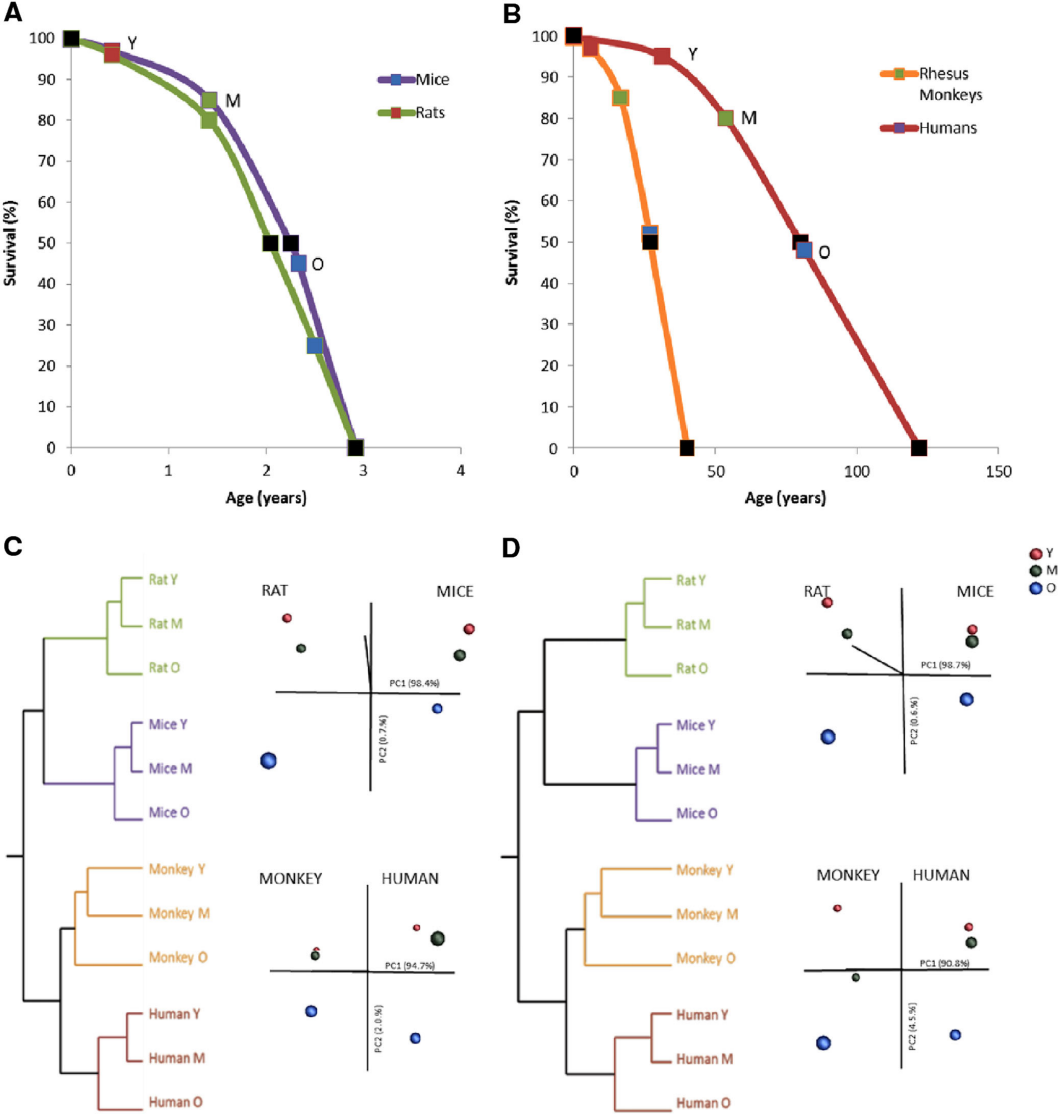

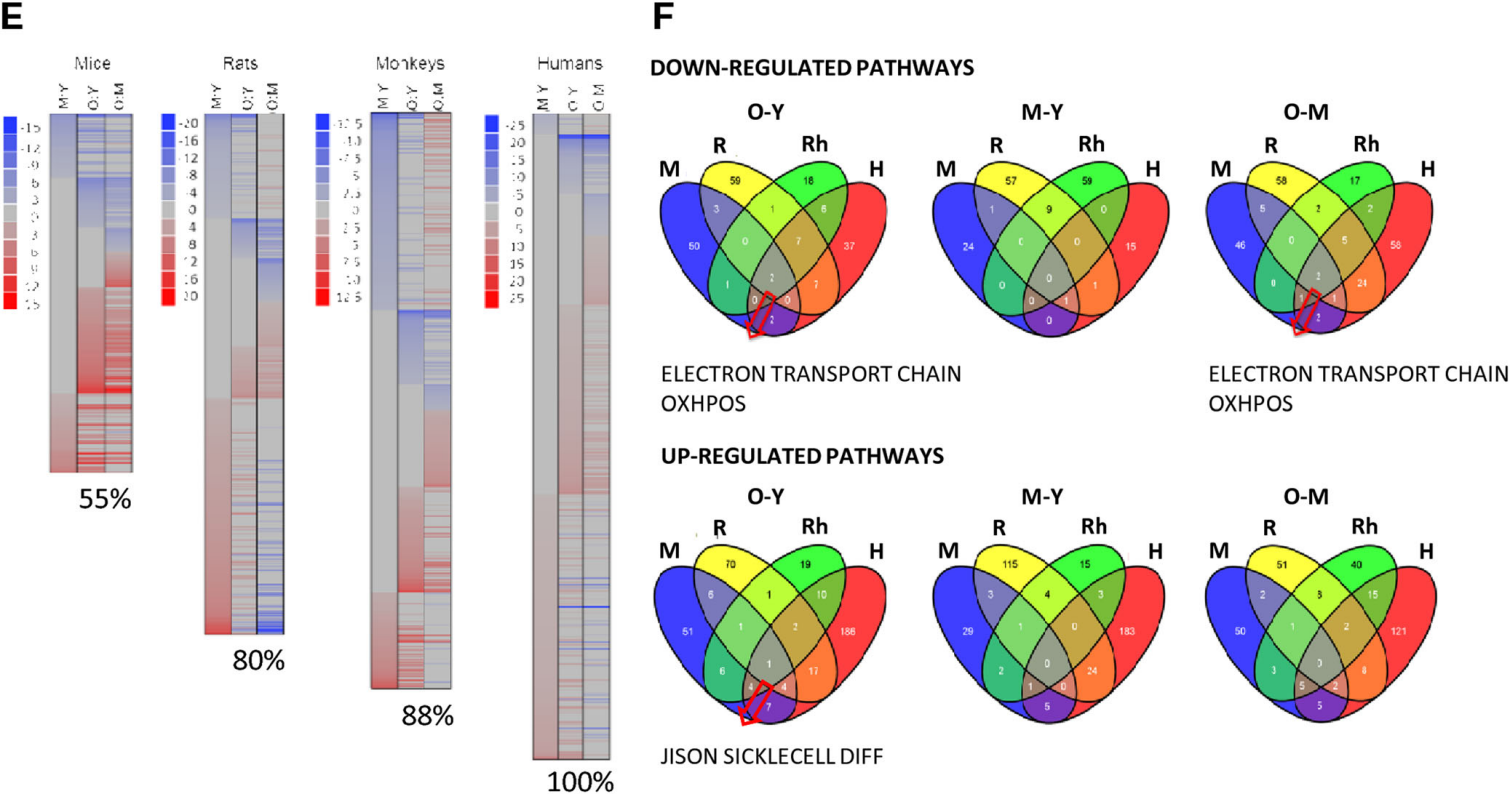
Identification of disrupted mitochondrial homeostasis as a common age-related signature across species. Mean and maximum lifespan curves for mice and rats (**a**), and rhesus monkeys and humans (**b**). Data were taken from the following references: mice mean^76^ and maximum^77^ lifespan, rat mean^78^ and maxium^78^ lifespan, rhesus mean^79^ and maximum^79^ lifespan and human mean (https://www.nia.nih.gov/research/publication/global-health-and-aging/living-longer) and maximum^80^ lifespan. *Black squares* represent mean and maximum lifespan, whereas *red*, *green*, and *blue squares* stand for young, middle-aged, and old species, respectively. PCA analysis from **c** genes and **d** pathways of mice, rats, rhesus monkeys, and humans of different ages are shown in *red* (Y, young), *green* (M, middle-aged), and *blue* (O, old). **e** Parametric analysis of gene-set enrichment (PAGE) was performed on micro-array data from different species. Columns show every gene set significantly upregulated (*red*) or downregulated (*blue*) by age. **f** Venn diagrams showing the up and downregulated gene set interactions between the different species and ages. Full names and Z-ratios for each of the gene set represented in the Venn diagrams are presented in the supplemental material (Table S5)

Next, we determined whether age modifies the gene expression profile in skeletal muscle of mice, rats, and rhesus monkeys in a pattern similar to what is observed in humans. We emphasize again that the three age groups selected (young, middle, and old) fall at similar time points along the lifespan curves of each organism (Fig. 1a, b). Principle component analysis (PCA) revealed a very distinct separation of the three age groups among each species for genes (Fig. 1c) and pathways (Fig. 1d). Notably, young animals/humans are more closely related to their middle-aged counterparts than to old individuals, and this pattern is consistent among all species studied. The pattern is also consistent with the idea that young and middle-aged groups fall closer to each other on the lifespan trajectory than the middle-aged and old groups (Fig. 1a, b).

To further define the role of age in modifying skeletal muscle gene expression in diverse species, we performed parametric analysis of gene set enrichment (PAGE), a computational method that determines differences between genes and pathways using *a priori* defined gene sets.^32^ PAGE analysis revealed that as one moves from mice to humans, pathway complexity increases, and as mammals age, there is predominantly an upregulation of pathways in all species (Fig. 1e, and Table S2). Furthermore, this analysis indicates that aging leads to perturbations in pathways associated with energy metabolism (i.e., oxidative phosphorylation, glucose, and fatty acid metabolism), diabetes (synthesis and function of insulin, IGF, and ghrelin), immune response and protein synthesis (Table S3). Our transcriptional profiling also revealed an age-induced increase in the inflammatory response (a list of the top 20 up and downregulated inflammatory genes can be found in Table S4). Notably, only two downregulated pathways, ETC and OXPHOS, were common among all four species in response to aging (Fig. 1f, and Table S5). To define the interacting networks and canonical pathways that are associated with muscle aging, ingenuity pathway analysis (IPA) was performed. IPA identified multiple genes encoding various canonical signaling pathways that were modulated by age, including mitochondrial dysfunction and the IGF-1/mTOR signaling pathways (Figs. S2–S4 from A–D). Collectively, our array data and subsequent analysis identify the highly conserved pathways of mitochondrial function, inflammatory responses, and nutrient sensing networks as common denominators of aging.

### Aging leads to altered mitochondrial composition

**Fig. 2:**
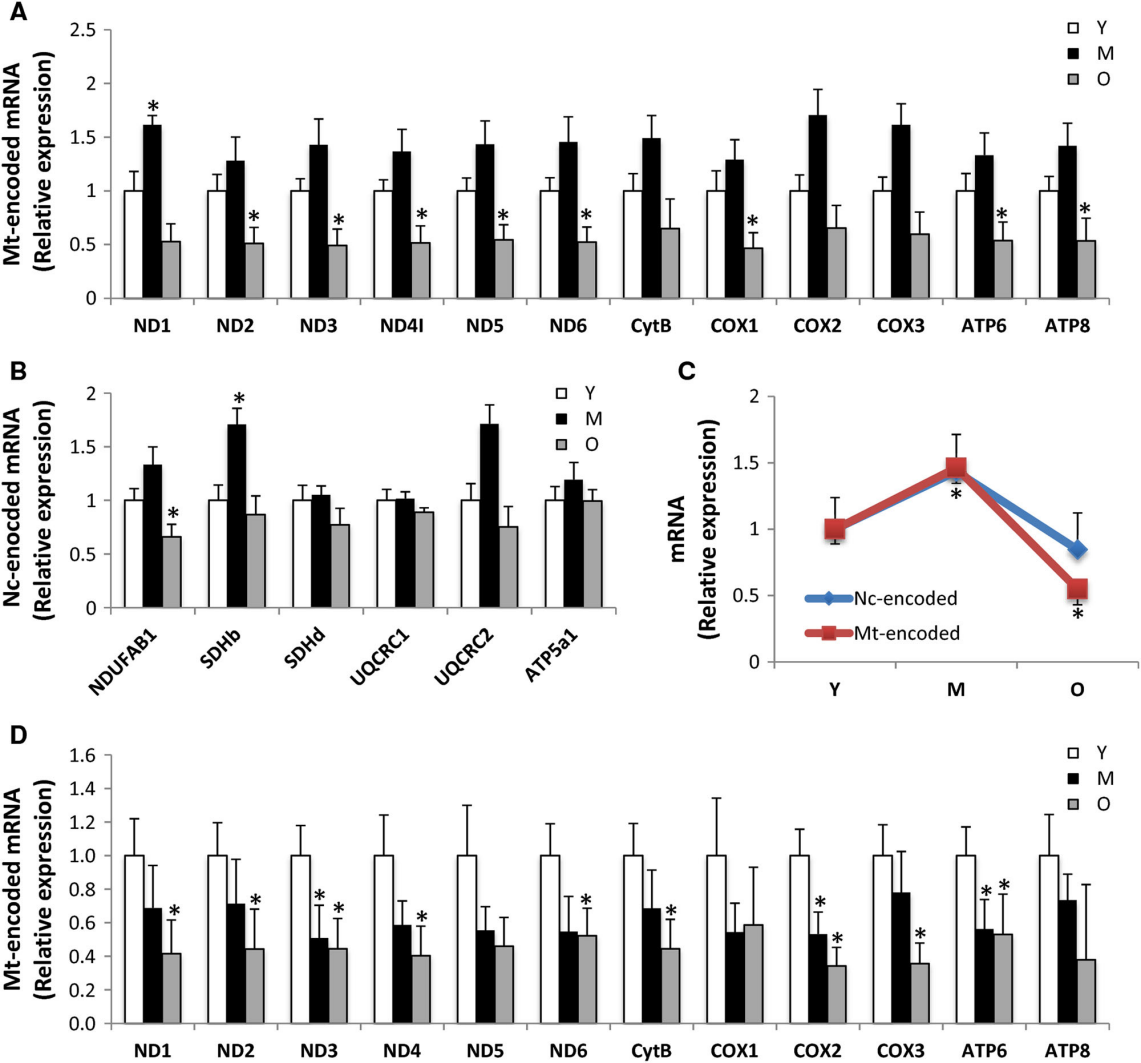

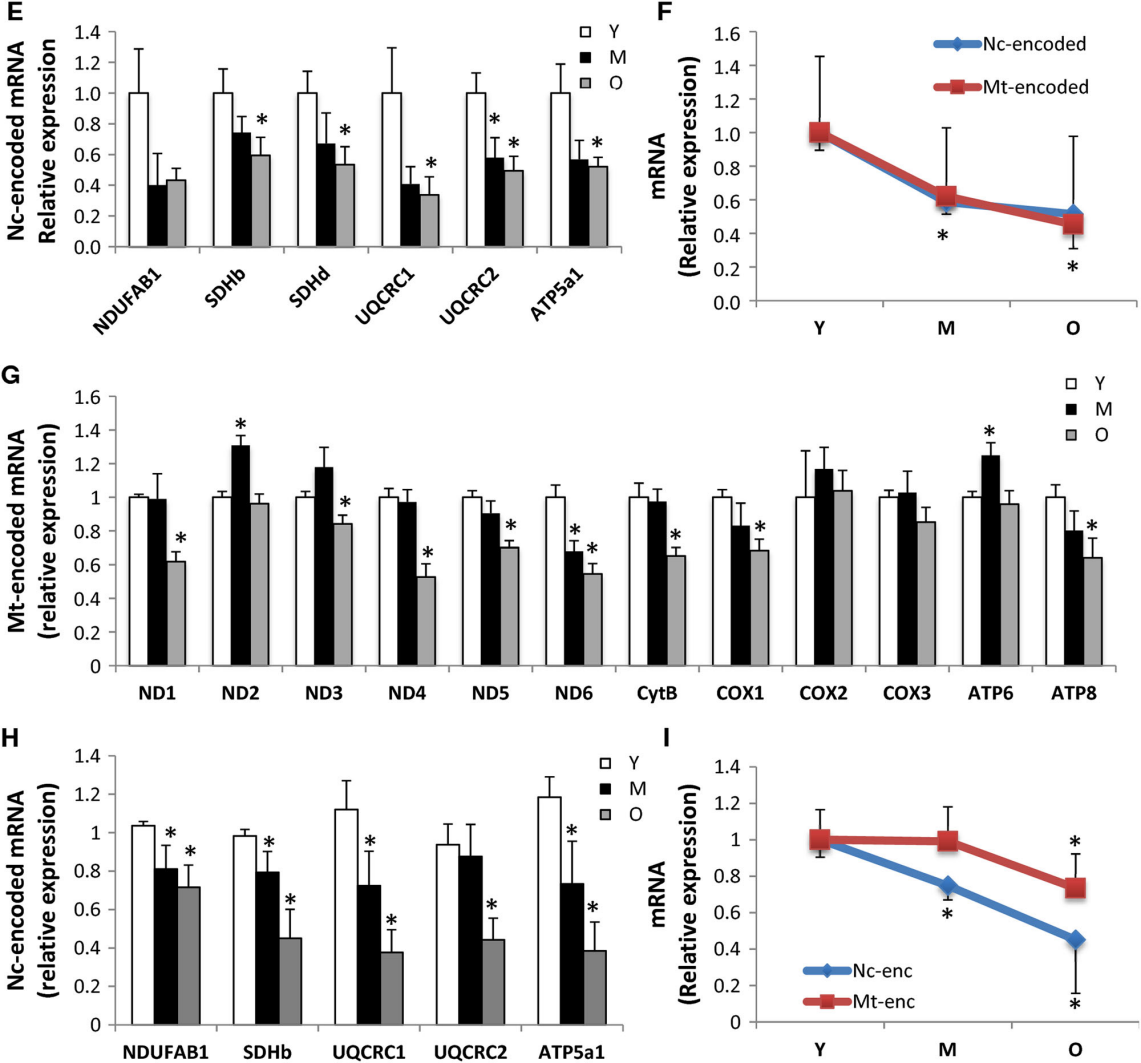

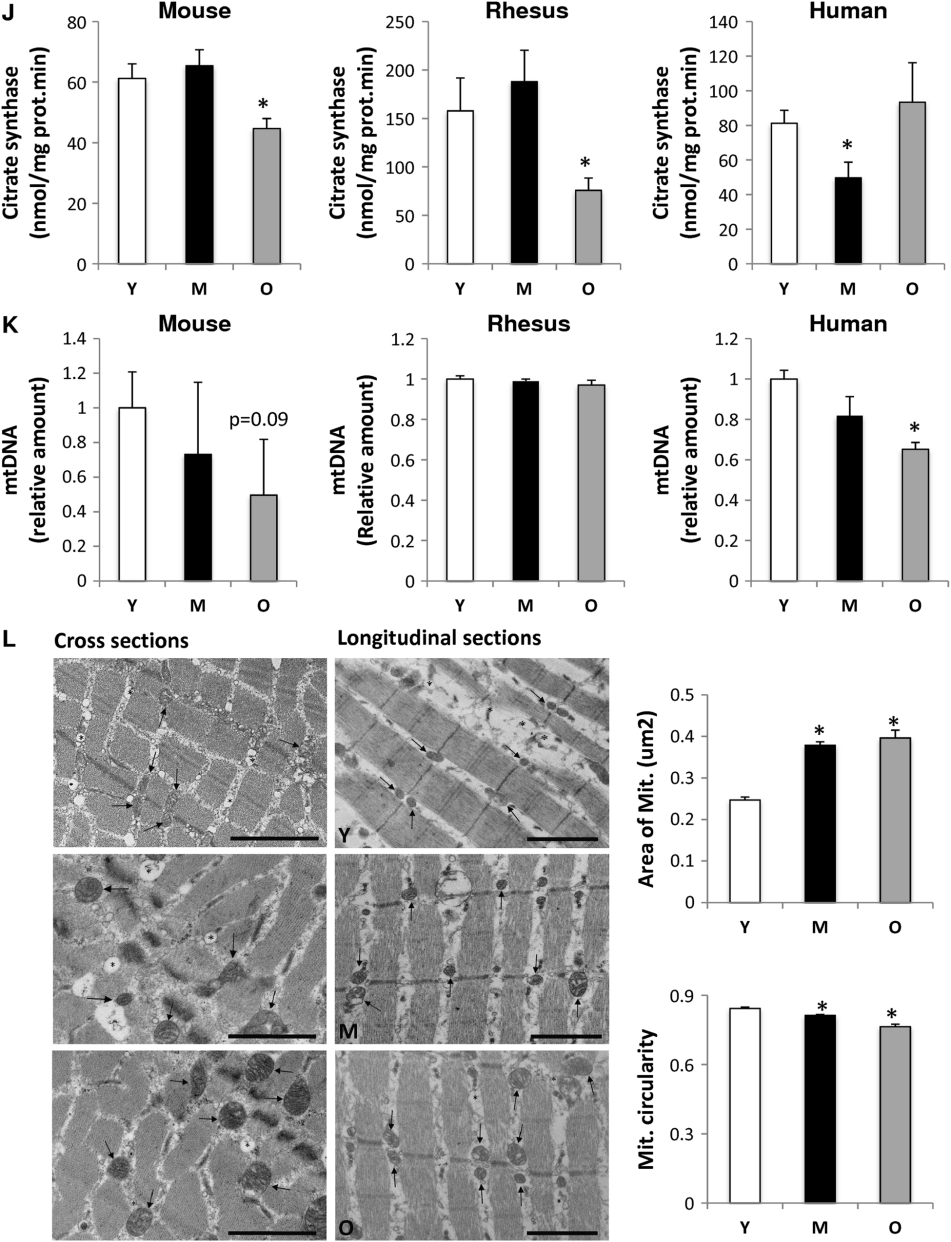

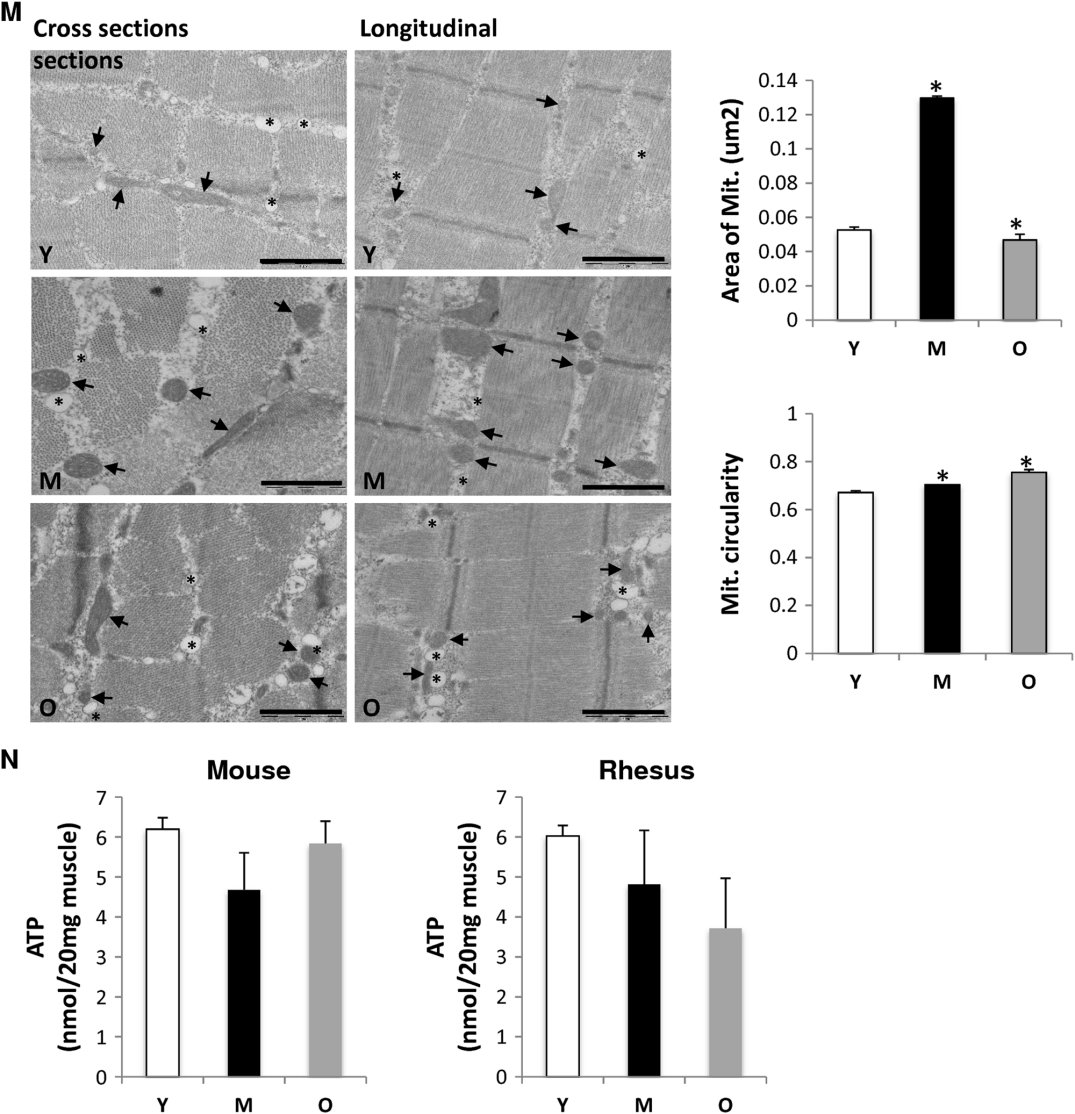
Mitochondrial composition is altered with age. Mitochondrial-encoded (ND1-6, CytB, COX1-3, ATP6, and ATP8) and nuclear-encoded (NDUFAB1, SDHb, SDHd, UQCRC1-2, ATP5a1) mRNA analyzed by quantitative RT-PCR in skeletal muscle of young (Y), middle-aged (M), and old (O) mice (M) (**a**–**c**), rhesus monkeys (Rh) (**d**–**f**) and humans (H) (**g**–**i**). Relative expression values were normalized to young within each species. **j** Citrate synthase activity measured in skeletal muscle of young (Y), middle-aged (M), and old (O) species. **k** Mitochondrial DNA content analyzed by quantitative PCR in skeletal muscle of young (Y), middle-aged (M), and old (O) mice, rhesus monkeys and humans. Relative expression values were normalized to young. Electronic microscopy analysis of skeletal muscle of young (Y), middle-aged (M), and old (O) mice **(l)** and rhesus monkeys **(m)** and the respective mitochondrial area quantification and circularity. *Arrows* indicate intermyofibrillar mitochondria; *asterisks* indicate sarcoplasmic reticulum. Bars equal to 1 µm. **n** Cellular ATP content from skeletal muscle of young (Y), middle-aged (M), and old (O) mice and rhesus monkeys. *p < 0.05 vs. young. Values are expressed as mean ± SEM

To gain insights into the molecular events underlying the putative mitochondrial dysfunction, we examined the effects of aging on mitochondrial content in skeletal muscle. The protein complexes of the OXPHOS system are encoded by the mitochondrial and nuclear genomes, and a coordinated control of these two genomes is required to maintain mitochondrial integrity. Quantitative PCR of a number of nuclear-encoded and mitochondrial-encoded transcripts revealed that the former are largely unchanged between young, middle-aged, and old mouse samples (Fig. 2a–c). Conversely, analysis of the mtDNA encoded genes indicated a general trend of reduced expression in the old mouse samples, which was statistically significant in 8 of 12 genes examined (Fig. 2a–c). In rhesus monkeys, an overall downregulation of both nuclear and mitochondrial-encoded OXPHOS messenger RNAs (mRNAs) was observed with age (Fig. 2d–f). Notably, in humans, expression of mitochondrial and nuclear-encoded transcripts decreased with age (Figs. 2g–i), even if a major preservation of gene transcription level in mitochondria than nucleus was found at least until middle age. Importantly, this is one of the major differences comparing different species in terms of mitochondrial transcripts.

To specifically assess the influence of age on markers of mitochondrial content in skeletal muscle, three different approaches were taken. First, we measured citrate synthase activity, an enzyme found in the mitochondrial matrix, and observed that this activity was significantly lower in the skeletal muscle of aged mice and monkeys compared to their younger counterparts (Fig. 2j). In humans, lower levels of citrate synthase activity were observed in middle-aged individuals as compared to young, yet no differences were observed between young and old participants (Fig. 2j). Second, we quantified mtDNA, which was found to be lower in the skeletal muscle of aged mice and humans, although this finding was not recapitulated in monkeys (Fig. 2k). Third, electron microscopy revealed that the mitochondrial size increased with age in the skeletal muscle of mice, in accordance with prior work,^33^ while in monkeys, mitochondrial size increased at middle-age, before decreasing in the old animals (Fig. 2l, m). Notably, during aging, a difference in the shape of mitochondria was observed in both aged mice and rhesus monkeys (Fig. 2l, m). Whereas the mitochondria were less rounded in old muscle of mice, a significant proportion of the organelles were more rounded in shape in the muscle of old monkeys (Fig. 2l, m). Although electron microscopy analysis was unable to be performed in humans due to limited material, previous studies have revealed that a significant proportion of mitochondria are abnormally enlarged and more rounded in shape.^34, 35^ Thus, despite the instances of variability, the overall picture indicates an altered expression or organization of mitochondrial components with age. Consistent with this conclusion, and in agreement with a previous study that showed a reduced mitochondrial ATP production in skeletal muscle of old healthy individuals,^36^ cellular ATP levels tended to be decreased in the skeletal muscle of old monkeys compared to their younger counterparts, whereas this was not seen in old mice (Fig. 2n). Thus, the results collectively indicate a conserved evolutionary age-dependent decrease in mitochondrial content and function.

### Aged skeletal muscle displays a decrease in OXPHOS complexes in primates

**Fig. 3:**
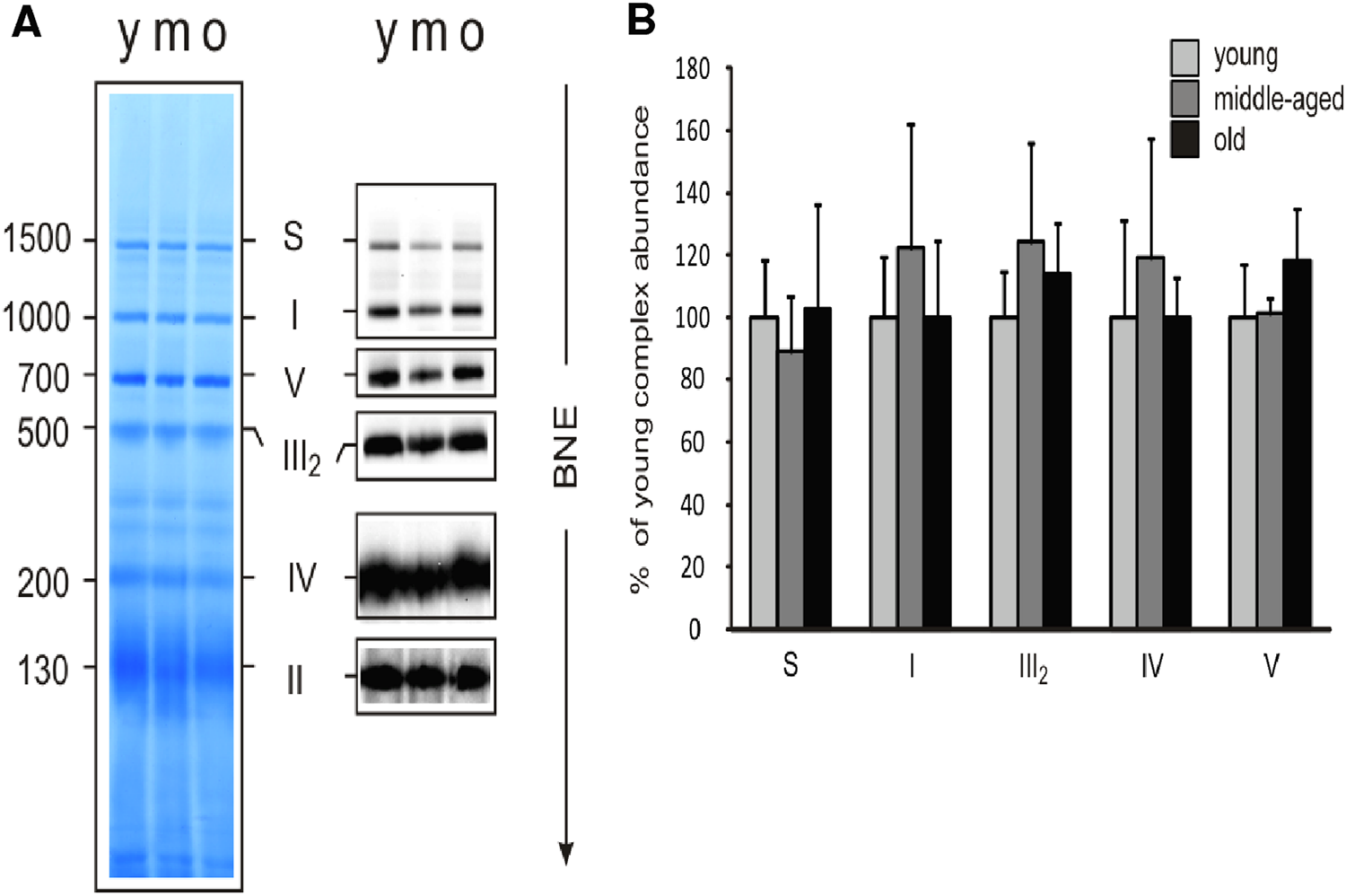
OXPHOS complexes remain unchanged in rat skeletal muscle with age. **a** Protein complexes from young (Y), middle-aged (M), and old (O) rat skeletal muscle tissue were isolated by blue-native electrophoresis and stained with Coomassie (*left* panel) or detected on a 1D BN western blot (*right* panel). Panels show a representative set of young, middle-aged and old tissue out of 15 samples (five individuals in each group). **b** Densitometric quantification of 1D BNE western blots. Signals of complexes and supercomplexes containing mitochondrial encoded subunits were normalized to complex II not containing mitochondrial encoded subunits (*n* = 5). Assignment of complexes: S, supercomplexes containing complex I, III, and IV with the stoichiometry of I_1_III_2_IV_0–4_; III_2_, dimer of complex III or cytochrome c reductase; IV, complex IV or cytochrome c oxidase; V, complex V, or ATP synthase

**Fig. 4:**
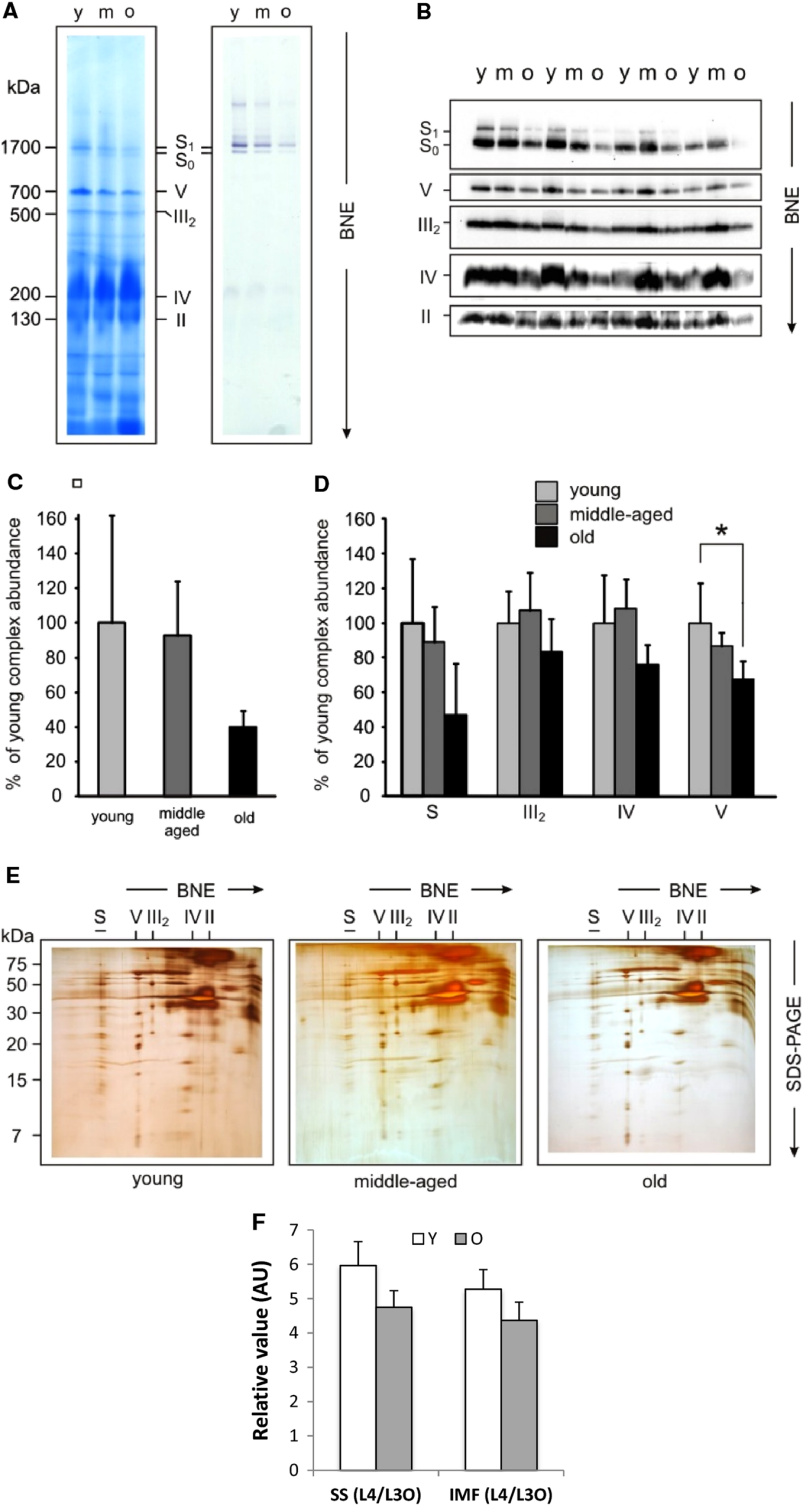
Aged skeletal muscle displays a decrease in OXPHOS complexes in primates. **a** Protein complexes from young (Y), middle-aged (M), and old (O) skeletal muscle from rhesus monkeys were isolated by blue-native electrophoresis (BNE) and stained with Coomassie (*left* panel) or *in-gel* complex I activity stain (*right* panel). Panels show a representative set of young, middle-aged, and old tissue out of 12 samples (four individuals in each age group). **b** Western blot detection of OXPHOS complexes and supercomplexes in 1D BN-blots using specific antibodies against complex I, II, III, IV, and V. **c** Densitometric quantification of in-gel activity stain (A, *right* panel) expressed as % of young complex I containing supercomplexes in four individuals each group. **d** Densitometric quantification of 1D BNE western blots. Signals of complexes and supercomplexes containing mitochondrial-encoded subunits were normalized to complex II not containing mitochondrial-encoded subunits (*n* = 4). **e** Protein complexes and subunit composition by 2D BN/SDS PAGE shown as silver stain from young (*left* panel), middle-aged (*middle* panel), and old (*right* panel). Assignment of complexes: S_0_, supercomplexes containing complex I and III with the stoichiometry of I_1_III_2_; S_1_, supercomplexes containing complex I, III, and IV with the stoichiometry of I_1_III_2_IV_1_; S, supercomplexes containing complex I, III, and IV with the stoichiometry of I_1_III_2_IV_0–4_; III_2_, dimer of complex III or cytochrome c reductase; IV, complex IV or cytochrome c oxidase; V, complex V or ATP synthase. **f** Skeletal muscle cardiolipin content (L4/L3O ratio) in subsarcolemmal (SS) and inter-myofibrillar (IMF) mitochondrial fraction from young (Y), middle-aged (M) and old rhesus monkeys. L4: tetra linoleoyl-cardiolipin, L3O: trilinoleoyl-oleoyl-cardiolipin. *p < 0.05 vs. young. Values are expressed as mean ± SEM

**Fig. 5:**
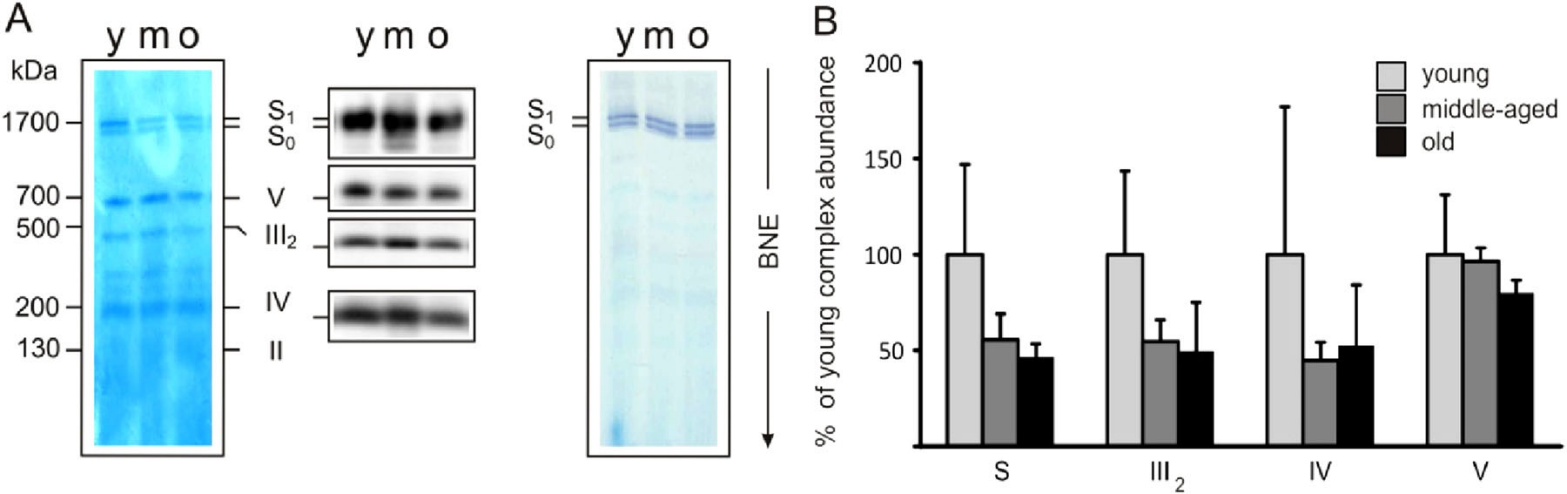
Patterns of changes in OXPHOS complexes in human skeletal muscle with age. **a** Protein complexes from young (Y), middle-aged (M), and old (O) human skeletal muscle tissue were isolated by blue-native electrophoresis and stained with Coomassie (*left* panel), detected on a 1D BN western blot (*middle* panel) and *in-gel* complex I activity stain (*right* panel). Panels show a representative set of young, middle-aged, and old tissue out of 9 samples (three individuals in each group). **b** Densitometric quantification of 1D BNE western blot (*n* = 3). Assignment of complexes: S, supercomplexes containing complex I, III and IV with the stoichiometry of I_1_III_2_IV_0–4_; III_2_, dimer of complex III or cytochrome c reductase; IV, complex IV or cytochrome c oxidase; V, complex V or ATP synthase

In mammalian mitochondria, the respiratory chain supercomplexes form ‘‘respirasomes’’ structures, which mainly comprise complexes I assembled to complex III and IV.^14, 37^ To elucidate whether aging alters structural organization of the respiratory chain, the composition of the different OXPHOS complexes in skeletal muscle lysates from rats, rhesus monkeys and humans was analyzed by blue native gel electrophoresis (BNE) in combination with in-gel activity using digitonin for solubilization, as this retains inner mitochondrial membrane supercomplexes (Figs. 3–5).^14^ Figure 3a shows the five OXPHOS complexes as well as the supercomplex S in the skeletal muscle of rats. No differences in the content of ETC complexes were observed in rat skeletal muscle upon age (Fig. 3b and Fig. S5A–F). However, unlike rats, coomassie- or silver-staining and subsequent densitometric analysis showed a combined age-related OXPHOS alteration in skeletal muscle of monkeys, with significantly lower amounts of complex V (Fig. 4a left panel,  + b, d–e and Fig. S6A–F). Densitometric analysis also revealed a tendency towards reduced levels of the supercomplex S in the old monkeys, indicative of less effective electron transport with age (Fig. 4b + d–e and Fig. S7).^38^ Additionally, an in-gel activity assay suggested a downregulation of complex I with age in muscle lysates of monkeys (Fig. 4a right panel + c, and Fig. S7), in line with the tendency for reduced supercomplex formation, as the presence of respirasomes is necessary for the stability and function of complex I. Cardiolipin, a phospholipid specific to the inner mitochondrial membrane, can also integrate into the structure of the respiratory complexes and aids in binding the complexes together into a supercomplex.^39^ A preliminary method was carried out to determine the ratio of tetra linoleoyl cardiolipin to tri-linoleoyl-mono-oleoyl cardiolipin (L4/L3O), the two most abundant cardiolipin species in muscle tissue, in subsarcolemmal and intermyofibrillar mitochondria in young and old monkeys. While, the data is not quantitative, the preliminary results suggest that the L4/L3O ratio trends lower in aged skeletal muscle (Fig. 4f). Although no significance was reached for the different complexes (supercomplex S, III, IV, V) in the skeletal muscle of humans due to the high degree of sample variability, the amount of all OXPHOS complexes was reduced with age (Fig. 5a, b and Fig. S8A–E). The above data show a commonality between rhesus monkeys and humans, indicating a reduction in OXPHOS complexes with age, an observation that is consistent with the IPA (Fig. S2A–D).

### Aging underlies altered mitochondrial dynamics and mitophagy

**Fig. 6:**
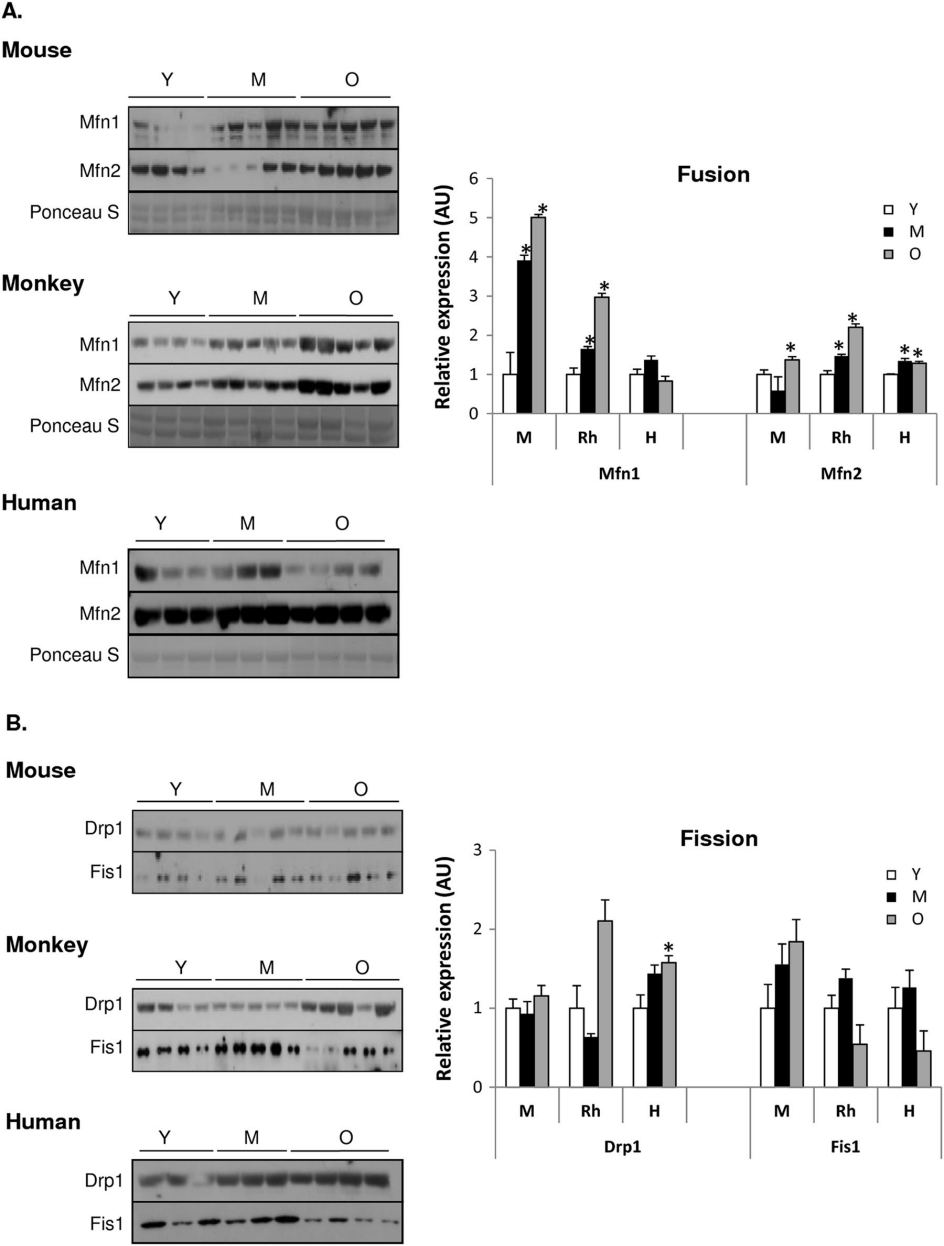

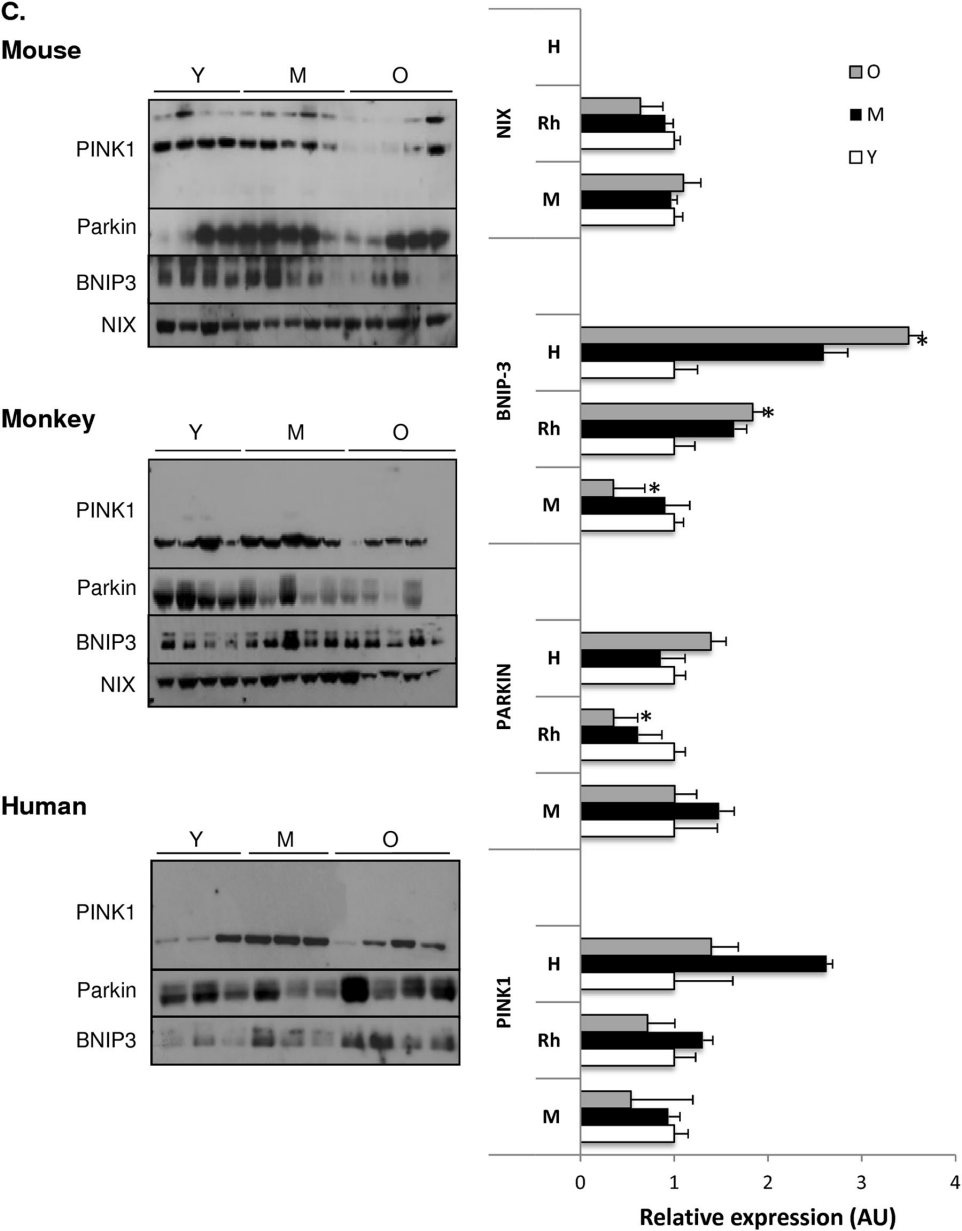
Mitochondrial dynamics and mitophagy are altered in an age-dependent manner. Immunblot of **a** fusion (Mfn1 and Mfn2), **b** fission (Fis1 and Drp1), and **c** mitophagy (PINK1, Parkin, NIX, and BNIP3) proteins in skeletal muscle of young (Y), middle-aged (M), and old (O) mice, rhesus monkeys and humans. Values are expressed as mean ± SEM. *p < 0.05 vs. young

Mitochondria are highly dynamic organelles, constantly undergoing cycles of fusion, fission and mitophagy in order to meet the bioenergetic requirements of the cell. Perturbations in mitochondrial dynamics precede cellular dysfunction and have been implicated in the progression of age-related diseases such as sarcopenia.^40^ To gain insight into the effects of aging on mitochondrial dynamics in skeletal muscle among species, we assessed key proteins involved in fusion ((mitofusins1 and 2 (Mfn1 and Mfn2)) and fission (fission 1 protein (Fis1) and dynamin-related protein (Drp1)). Mfn1 levels were significantly upregulated with age in both mice and rhesus monkeys, with the effect being most pronounced in the oldest animals, whereas no differences were detected within the human group (Fig. 6a). Notably, an age-dependent increase was observed for Mfn2 among all species (Fig. 6a), implying that fusion is upregulated in skeletal muscle during aging and that this effect is evolutionarily conserved. These data are in line with the altered mitochondrial morphology revealed by transmission electron microscopy (TEM), as illustrated by the appearance of enlarged mitochondria in muscle of aged mice and abnormally rounded mitochondria in the muscle of aged rhesus monkeys (Fig. 2m). While no age-related changes were observed in fission proteins Drp1 and Fis1 among mice and rhesus monkeys, a significant increase in Drp1 protein was detected in elderly humans compared to young (Fig. 6b). Collectively, our data suggest that a fusion–fission imbalance toward mitochondrial fusion occurs in aged skeletal muscle.

We next looked at two programs of mitophagy: selective mitophagy (PINK1/Parkin) and programmed mitophagy (NIX/BNIP3). The mitochondrially targeted serine/threonine kinase PTEN-induced putative kinase 1 (PINK1) and the E3 ubiquitin ligase Parkin are known to selectively promote the degradation of damaged mitochondria.^41^ Aside from the downregulated Parkin protein levels in old rhesus monkeys compared to young, no significant age-related changes were observed for the PINK1/Parkin protein levels across species (Fig. 6c). In addition, NIX and its related family member, Bcl2/adenovirus E1B interacting protein 3 (BNIP3), are unique mitochondria-localized BH3-only proteins and appear to be able to compensate when the PINK1/Parkin pathway is deficient. Whereas no age-related alterations were observed for NIX protein levels across species, BNIP3 protein levels were reduced in old mice compared to their younger counterparts (Fig. 6c). In contrast, old rhesus monkeys and humans exhibited increased BNIP3 protein levels compared to young (Fig. 6c). Nonetheless, mitophagy seemed not to be significantly altered overall with age across species, suggesting that damaged mitochondria are too large, due to increased fusion, to be engulfed by the autophagosome, favoring the accumulation of aberrant proteins and damaged organelles. This outcome might contribute to the reduced age-associated mitochondrial function observed among species.

### Aging provokes increased oxidative stress and inflammation among species

**Fig. 7:**
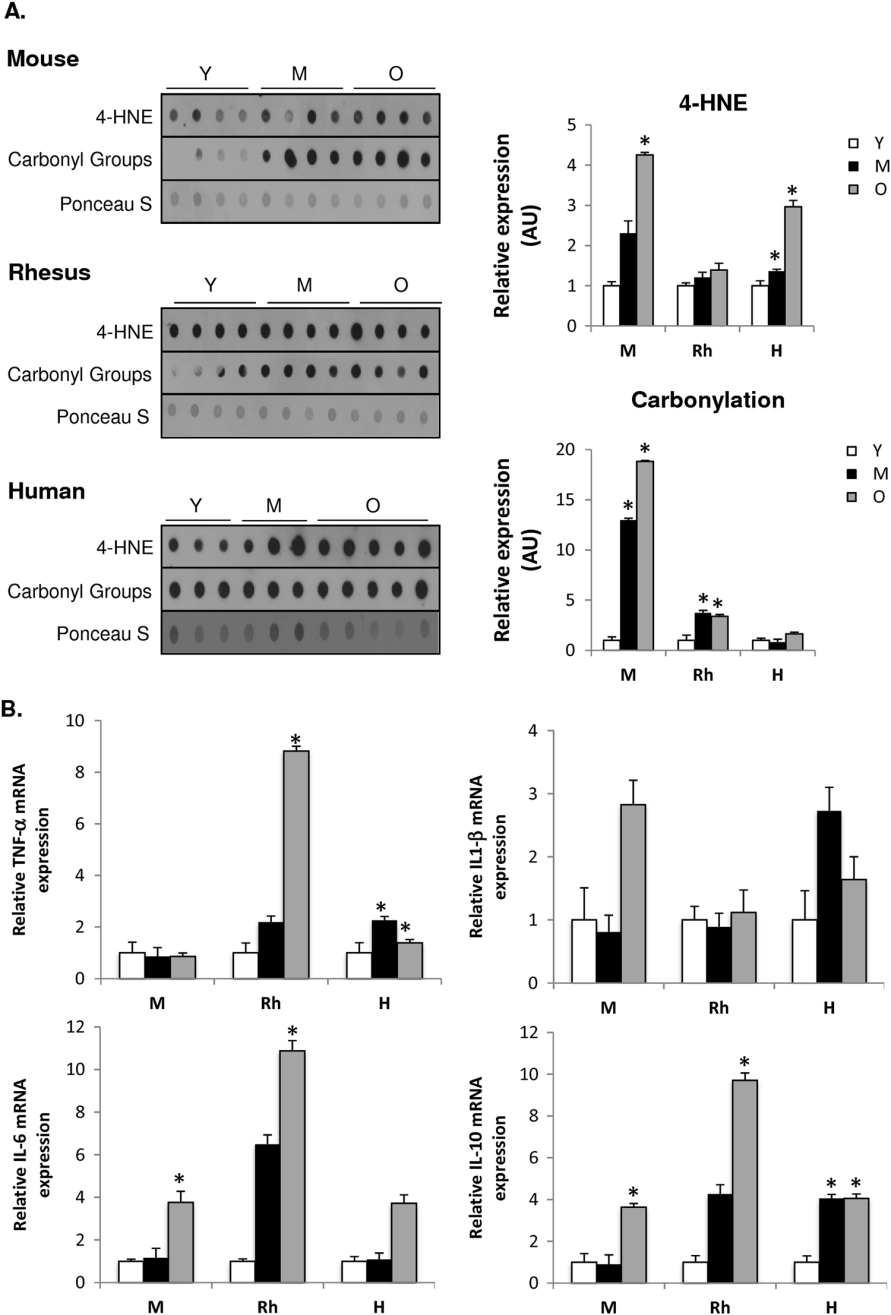

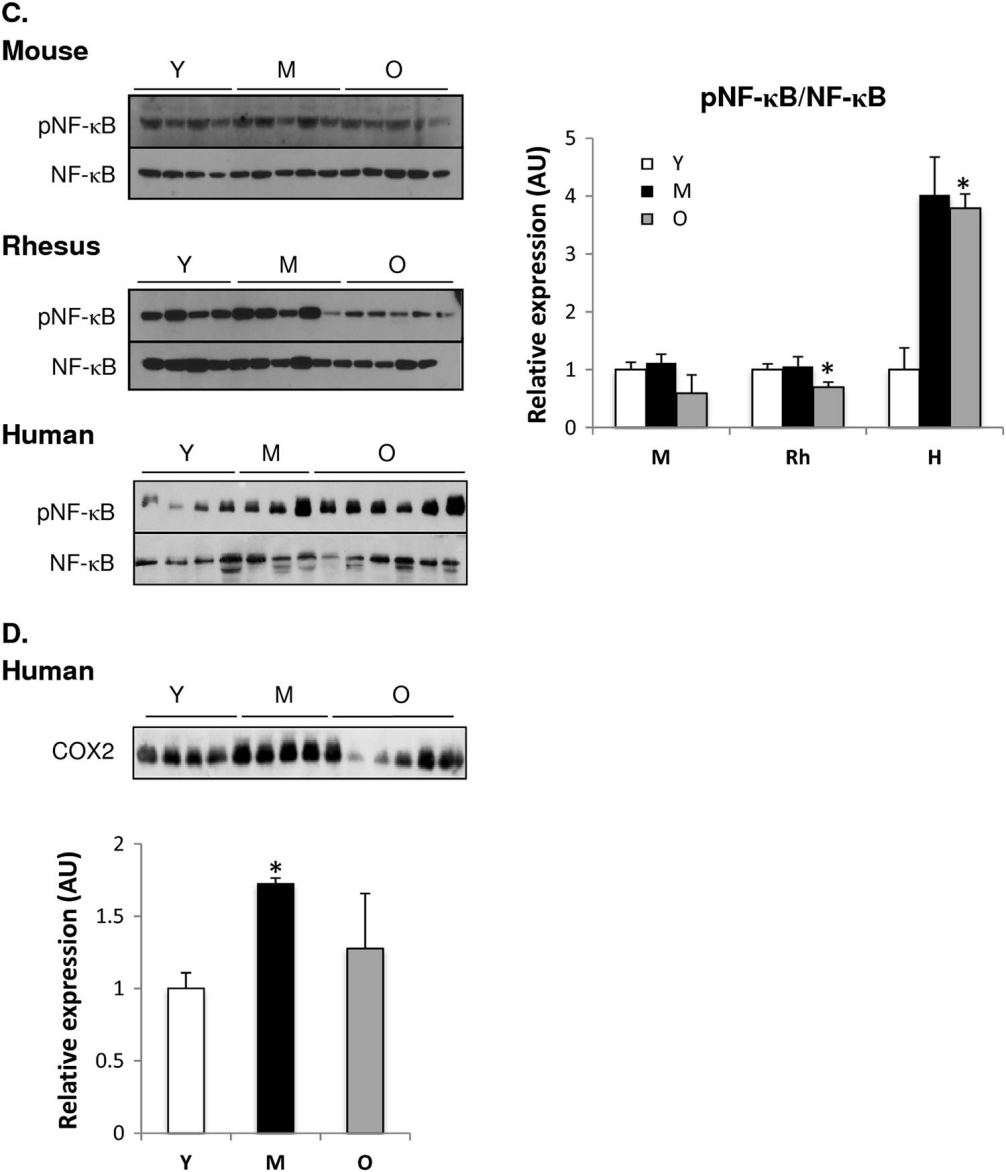
Increased oxidative stress and inflammation are observed with age among species. **a** Oxidative stress markers (4-HNE and protein carbonylation) in skeletal muscle of mice (M), rhesus monkeys (Rh), and humans (H). **b** Inflammatory mRNA markers (TNF-α, IL-1β, IL-6, and IL-10) analyzed by quantitative RT-PCR in skeletal muscle of mice (M), rhesus monkeys (Rh), and humans (H). Relative expression values were normalized to young within each species. **c** Immunoblot of pNF-κB and NF-κB in skeletal muscle of young (Y), middle-aged (M), and old (O) mice, rhesus monkeys and humans. **d** Immunoblot of COX2 in human skeletal muscle of young (Y), middle-aged (M), and old (O) individuals. *p < 0.05 vs. young. Values are expressed as mean ± SEM. *p < 0.05 vs. young

As mitochondrial oxidative stress is considered a hallmark of cellular aging, we evaluated age-related effects on oxidative stress markers in skeletal muscle of mice, rhesus monkeys and humans. 4-hydroxynonenal adducts, a marker of lipid peroxidation, were increased in skeletal muscle of old mice and humans compared to young, whereas no age-related effect was detected in the muscle of monkeys (Fig. 7a). Moreover, an age-related increase was observed for protein carbonylation in mice and rhesus monkeys, with the effect being most pronounced in the muscle of older animals (Fig. 7a). These data collectively indicate that oxidative stress markers are more prominent with age in mice than in higher mammals, yet can be observed in some manner in all species suggesting a possibly conserved phenomenon.

Human aging is characterized by a state of chronic low-grade inflammation, designated as ‘‘inflamm-aging’’.^24, 42^ An increased transcript level in the pro-inflammatory cytokine interleukin 6 (IL-6) was observed in skeletal muscle of old mice (Fig. 7b). In the skeletal muscle of rhesus monkeys, an age-related upregulation of tumor necrosis factor-α (TNF-α) and IL-6 expression was observed with age; similar effects were observed in skeletal muscle of humans (Fig. 7b). Among all species, skeletal muscle exhibited elevated expression levels of anti-inflammatory IL-10 with age (Fig. 7b). In addition, we looked at the NF-κB transcription factor, as it is a master regulator of inflammation through its ability to induce transcription of pro-inflammatory genes.^43^ The ratio of phospho-active to total NF-κB was not affected with age in skeletal muscle of mice, while a decrease in active NF-κB levels was observed in muscle of old monkeys compared to young (Fig. 7c). In line with the upregulation of pro-inflammatory cytokines with age in human skeletal muscle, an age-induced up-regulation of active NF-κB protein levels was observed (Fig. 7c). The increased inflammatory response in aged human skeletal muscle is further evidenced by the increased cyclooxygenase-2 (COX2) expression with age^44^ (Fig. 7d). These observations suggest that the skeletal inflammatory profile differs with age between species, but appears to be most pronounced in humans.

### Deregulated nutrient-sensing signaling pathway across species

**Fig. 8:**
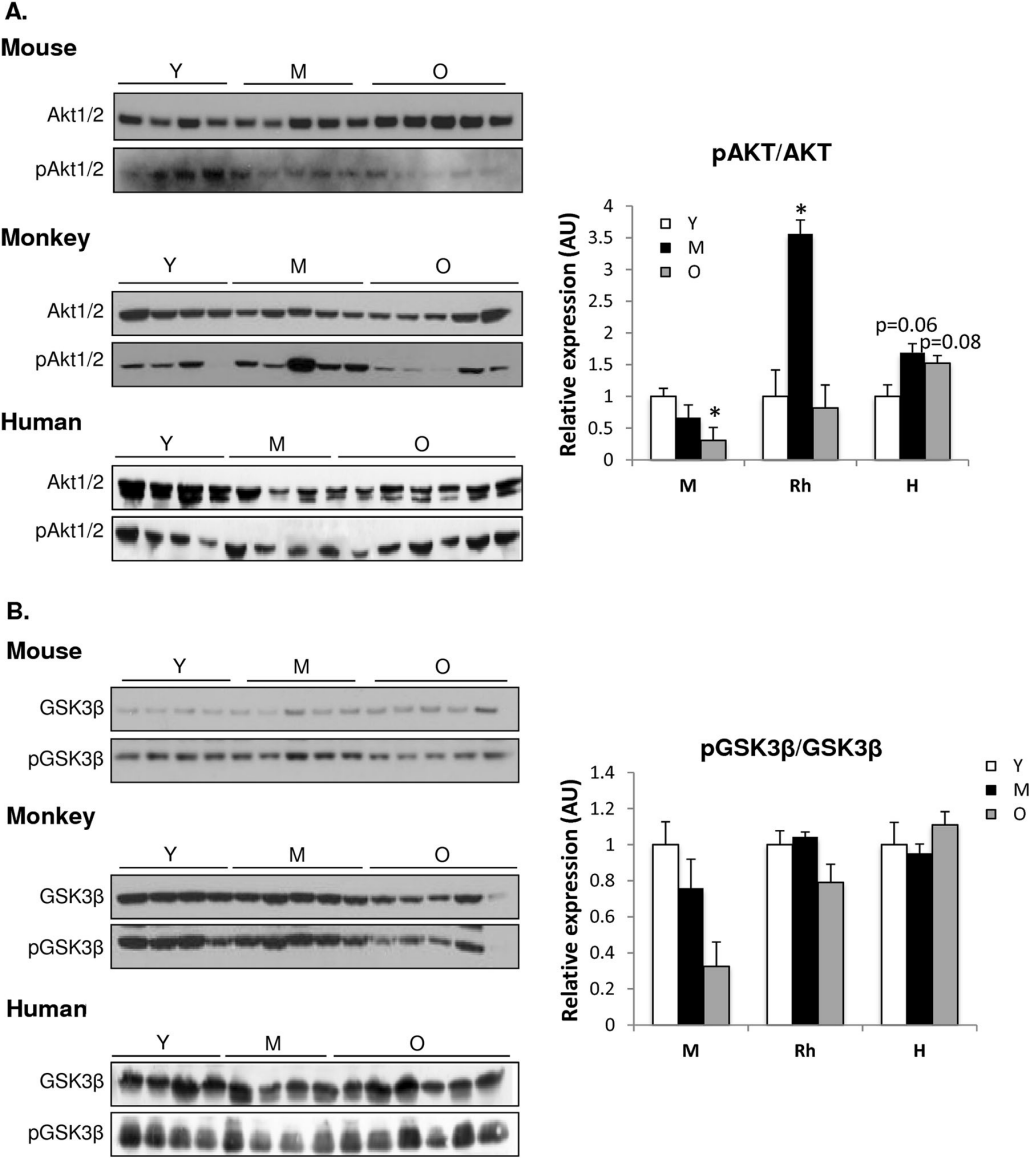

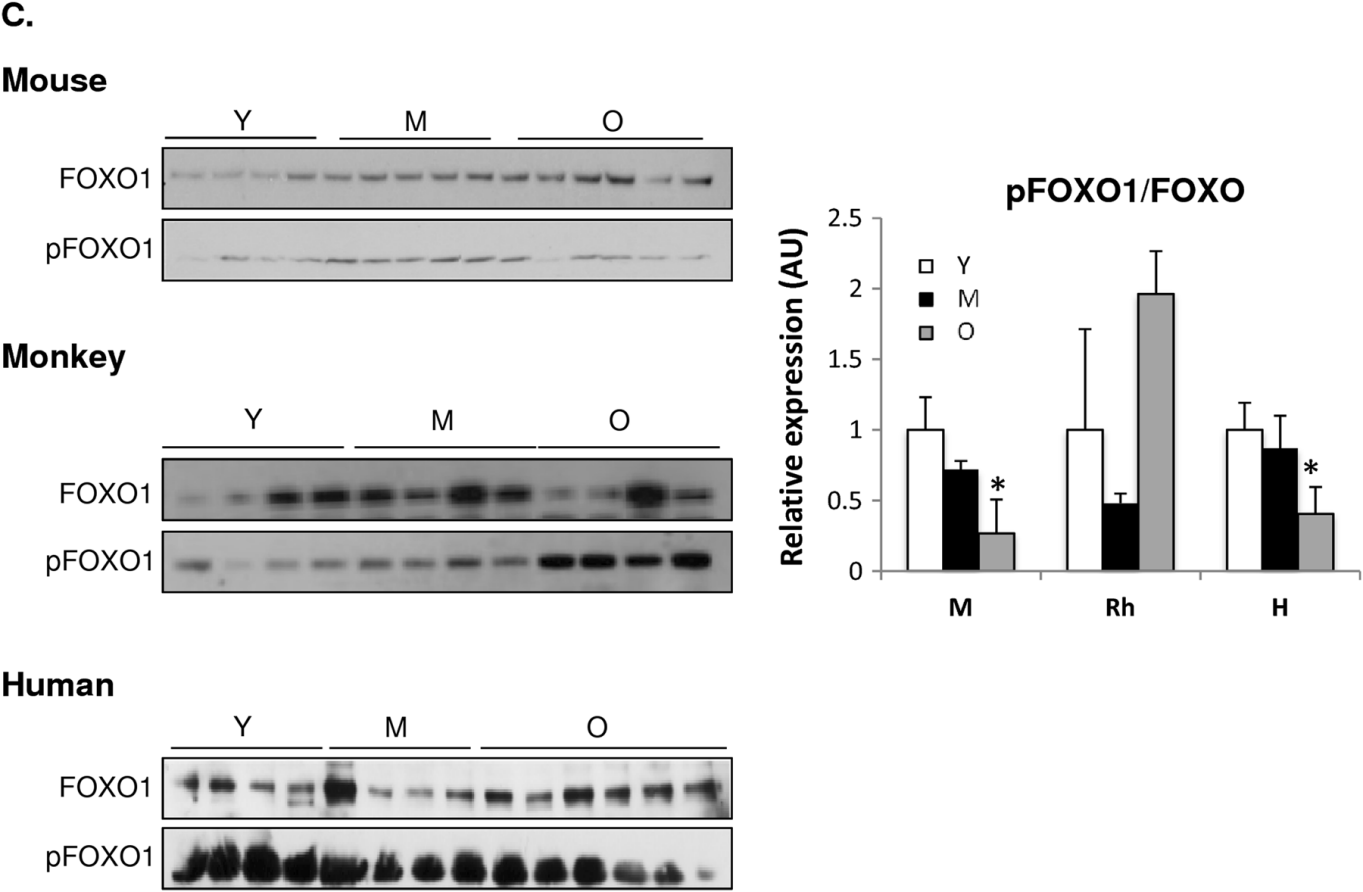
Nutrient-sensing signaling pathway is deregulated across species with age. Immunoblot of **a** pAKT1/2 and AKT1/2 **b** pGSK3β and GSK3 **c** pFOXO1 and FOXO in skeletal muscle of young (Y), middle-aged (M), and old (O) mice, rhesus monkeys, and humans. *p < 0.05 vs. young. Values are expressed as mean ± SEM. *p < 0.05 vs. young

The IGF-AKT-mTOR axis is a well-known conserved signaling pathway in aging that is involved in protein synthesis.^1, 45^ As a read out of protein turnover, we measured AKT and its main up-stream and down-stream effectors in skeletal muscle. We observed that AKT activation was downregulated in skeletal muscle of old mice, as lower phosphorylated AKT levels were detected by western blot analysis (Fig. 8a). Interestingly, phosphorylated AKT was increased in middle-aged monkeys, whereas in humans, a tendency toward increased AKT activation was seen with age (Fig. 8a). Among the downstream effectors of AKT, pGSK3ß, and pFOXO1 protein levels were lower in skeletal muscle of old mice, suggesting increased protein degradation with age (Fig. 8b, c). In skeletal muscle of monkeys and humans, pGSK3ß levels were unaltered with age, whereas similar to mice, reduced pFOXO levels were observed in the older human cohort (Fig. 8b, c). These data are supportive of the IPA, showing that the mTORC1 complex is upregulated in monkeys and humans, yet downregulated in mice with age (Fig. S3). Of note, the mTORC2 complex, known to regulate cytoskeletal organization and cell survival/metabolism, was upregulated with age in all species (Fig. S3).^26^ Two key downstream targets of the mTORC1 complex, S6 kinase (S6K) and the eukaryotic translation initiation factor 4E–binding protein (4E-BP), which both play important roles in the initiation of mRNA translation, were unchanged with age in all species (Fig. S3).^46^ However, many of the translation components, such as the eIF4A-binding protein, were increased in the old group in all species (Fig. S3). Upstream effectors of AKT, such as IRS1/2, were downregulated in all species with age, whereas IGF-1 is downregulated in mice and humans, but is unchanged in monkeys (Fig. S4). Unlike the other molecular denominators of aging described above, within the nutrient sensing pathways, there are important similarities, but also significant differences among species with age.

## Discussion

In the studies presented herein, we compared a collection of molecular and biological end-points among multiple mammalian species, i.e., mice, rats, rhesus monkeys, and humans, at comparable ages along the trajectory of their respective lifespans. Our analysis has uncovered several species-unique and generally conserved features of mammalian aging. Of particular note, we observed the involvement of four of the nine ‘‘Hallmarks of Aging’’^1^ in each of the mammalian species studied herein: mitochondrial dysfunction, oxidative stress, inflammation, and nutrient-sensing pathways.

In terms of body composition, we observed significant changes with age. In particular, we saw an age-related increase in bodyweight in all species. However, the reason behind the increase was different among the species. Specifically, uniquely in mice, bodyweight increased due to an increase in lean mass, while fat mass was lower in the older animals. Conversely, in rats, monkeys and humans, the increase in bodyweight stemmed from an increase or no change in fat mass, while lean mass decreased with advancing age (results herein and).^30, 47, 48^ The unique feature of mice raises caution about the use of this model organism in understanding many age-related diseases, but particularly those involving metabolic processes.

Notably, our array studies identified only two “pathways” that were downregulated in the species studied herein: the mitochondrial ETC and OXPHOS. Previous transcription profiles of aging in the skeletal muscle of worms, flies, mouse, rats, and humans also indicate that genes encoding mitochondrial components decrease with age.^31, 49, 50^ Thus, research over the last decade has commonly found an association between mitochondrial dysfunction and major phenotypes associated with aging, including muscle decline. Importantly, mitochondrial transcript levels of humans appeared the most preserved, at least until middle age, likely suggesting an evolution-driven fitness more robust than other species. That said, the observed species-specific alterations in gene expression emphasize the importance of studying aging in humans in order to understand the mechanisms that modulate life- and health-span in people.

Coordination of expression of the nuclear and mitochondrial genomes is crucial for optimal organism function, and studies have found that this coordination is disturbed with age.^51, 52^ In general agreement with prior work,^51^ our analysis revealed discoordination between the regulation of the mitochondrial and nuclear genomes. Specifically, we observed downregulation of the mitochondrial-encoded genes in all species with age, although the timing, middle-age or old, was different among species. Overall, the results regarding the expression patterns of the nuclear and mitochondrial genomes at oldest ages are consistent with impaired compartment coordination and likely reflect the observed mitochondrial dysfunction.

OXPHOS component analysis uncovered that muscle extracts from old rhesus monkeys and humans contained an aberrant composition of several of the mitochondrial membrane respiratory complexes, including a tendency towards a decrease in the supercomplexes with age (although rats did not exhibit this phenotype). In addition, despite the fact that there was species-specific variability in some of the individual parameters, our studies revealed an age-related reduction in mitochondrial content in all species, as assessed by citrate synthase (CS), mtDNA, and TEM experiments, implying an underlying role for mitochondrial dysfunction in the aging process. These findings agree well with the recent work of Ikeda et al.^15^, who reported that in mice mitochondrial activity is impaired and ATP content decreased due to a lack of supercomplex formation. Notably, a previous study using primary skin fibroblasts from long-lived individuals (centenarians) found that supercomplexes were increased, possibly as a compensatory mechanism to facilitate efficient respiratory chain activity.^53^ It is likely that this apparent discrepancy represents adaptation changes that are not seen in the old of the cohort studied herein, but that are unique to centenarians.

Mitochondrial morphology is altered during aging in a process governed by the fission and fusion machinery. In our studies, we observed that fusion is increased in all species with age (as evidenced by the higher protein levels of Mfn1/Mfn2 relative to younger controls), whereas fission shows variable and limited differences. An imbalance in mitochondrial dynamics towards fusion is associated with the appearance of abnormal mitochondria, including those that are enlarged or more rounded in shape, as seen by TEM in mouse and monkeys, respectively. This hyperfusion phenotype may stem from an attempt to cope with an increase in mtDNA mutations by dilution,^54^ creating giant mitochondria in the process that are unable to be efficiently removed by the autophagosome. Our observation that there are no consistent changes in mitophagy with age in any of the species suggests that while this system may remain intact, it is incapable of dealing with the hyperfusion network, ultimately resulting in mitochondrial dysfunction. We note that prior evidence has revealed a decline in the autophagic process during normal aging in invertebrates and higher organisms.^55^

Additionally, we measured a gradual increase in oxidative stress markers over the lifespan of all species. This finding is by-and-large consistent with prior studies that have indicated an increase in oxidative macromolecular damage with age, presumably resulting from aberrant mitochondrial activity.^56, 57^ Thus, our collective studies support that with age, mitochondrial damage progressively increases, leading to altered mitochondrial composition and morphology, which further exacerbates the mitochondrial dysfunction and promotes increased fusion as a compensatory mechanism for the consequent ATP demands.

Another notable feature of our analysis was the increase in variable inflammatory markers with age in all species. Inflamm-aging was most pronounced in humans, involving a unique age-related upregulation of the transcription factor NF-κB as previously shown.^58^ It is tempting to speculate that the distinct inflammatory responses among the different species stems at least in part from the fact that humans exist in an environment accompanied by unique exposures and lifestyles, including a widespread contact with antigenic elements. Our results, therefore, indicate that in addition to oxidative stress and mitochondrial dysfunction, inflammation plays a significant role in the aging process, as previously proposed.^59, 60^

An additional element that we observed during our analysis is the likely involvement of the nutrient sensing pathways in the aging phenomenon. Associations have been previously introduced between mitochondrial aging and the IGF-1 signaling and mTOR pathway.^61, 62^ One notable observation of our study was the deregulated AKT response with age. In mice, the AKT response, as measured by phosphorylated AKT, was found to be downregulated, as was mTORC1, indicating a favoring of protein degradation, consistent with the increased GSK3β and FOXO1. Conversely, in humans, the AKT response was generally upregulated, revealing species-specific alterations during aging. Based on our IPA, we surprisingly observed that mTORC1 was upregulated in humans and monkeys, indicating that protein synthesis may be promoted as a consequence of the aging process. It is possible that the age-related muscle atrophy in primates is accompanied by a compensatory response that attempts to retain muscle integrity, but fails due to impaired cellular function, as suggested by a previous study.^63^ Our data in total paints a picture in which age-dependent alterations in oxidative stress, inflammation, and the nutrient signaling pathways ultimately affect the quality and status of mitochondria.

Notably, the age-related mitochondria impairment also affect energy metabolism leading to the alteration of beta-oxidation with an increase of intra-fibers lipid accumulation that affects skeletal muscle quality and function, as previously shown both in human and denervation-induced atrophy in mice model.^64, 65^

The current work highlights the molecular denominators of aging process among different mammalian species, including humans, with different lifespan. Mitochondria and their status are at the core of commonalities tightly related to evolution constrains such as natural selection and fitness, but also effects of environments/life style and food intake. Not surprisingly, many mutations that extend lifespan in animal model affect signaling pathways regulating the metabolicrate and human longevity is associated with efficient respiratory chain activity and mitochondrial genetics.^53, 66^

An important challenge looking forward is the development of therapeutic strategies that improve mitochondrial function as a means to delay the onset of age-related diseases, keeping in mind the important species-specific and even individual-specific adaptations.

## Material and methods

### Study species

Young, middle-aged, and old male skeletal muscle samples from C57BL/6 mice, Fisher-344 rats, rhesus monkeys (*Macacamulatta*), and humans were used in this study. Study species characterizations can be found in Table 1. C57BL/6J mice were obtained from the Jackson Laboratory (Bar Harbor, ME, USA) and housed at the Gerontology Research Center (Baltimore, MD, USA). Mice were housed in cages of four with ad libitum access to diet and tap water. Mice were fed house chow (HarlanTeklad Global 18% Protein Rodent Diet, Harlan Teklad, Madison, WI). Rats were fed a NIH-31 standard chow (Harlan Teklad, Indianapolis, IN, USA) and were singly housed in an environmentally controlled vivarium with unlimited access to water. Animal rooms were maintained at 20–22 °C with 30–70% relative humidity and a 12-hour light/dark cycle according to animal protocols and NIH guidelines. Gastrocnemius and vastus lateralis muscles were harvested from mice and rats, respectively. Rhesus monkeys were housed continuously at the NIH Animal Center, Poolesville, MD. Monkeys were housed individually in standard nonhuman primate caging on a 12 h light/12 h dark cycle, room temperature 25.5 ± 0.5 °C, humidity at 60 ± 20%. Monkeys were maintained on standard monkey chow (TestDiet® #5038 Purina Mills, Richmond, IN). They received two meals per day at estimated ad libitum levels throughout the study and water was always available. Monkeys were monitored minimally three times daily by trained animal care staff. Biopsies of the *vastus lateralis* were obtained from the monkeys from ongoing NIA studies. All animal procedures for this study were reviewed and approved by the Animal Care and Use Committee (ACUC) at the Biomedical Research Center (NIA/NIH).Humans who underwent surgery for hip dysplasia were included in this study. During surgery, biopsies from the *vastus lateralis* muscle were obtained. Age (>20 years) and ability to provide informed consent cooperating with study personnel were inclusion criteria, while exclusion criteria were presence of diseases, i.e., chronic kidney and/or liver diseases, bleeding disorders, severe type 2 diabetes, rheumatic diseases, osteoarthritis, neuromuscular disorders, malignancies and systemic infections, other than chronic steroid use, major psychological problems or history of alcohol or drug abuse, evidence of prior surgery in the involved hip. Written informed consent was obtained from each patient before the kick off study. A thorough medical history, including smoking habits and alcohol consumption, occupation and level of physical activity was taken from each patient and reported in questionnaire forms. Written informed consent was obtained from all participants, and the study was approved by the ethics committees of Istituto Ortopedico Rizzoli and Sant’Orsola-Malpighi University Hospital (Bologna–Italy). With the exception of tissues processed for electron microscopy, muscle samples for all study species were flash frozen in liquid nitrogen and stored at −80 °C until assayed. Gastrocnemius (mice) and *vastus lateralis* (rat, monkey, human) muscles were used for all biochemical assays and to generate the micro-array data.

### Body composition

#### Mice and rats

Measurements of lean, fat, and fluid mass in live mice and rats were acquired by nuclear magnetic resonance using the Minispec LF90 (Bruker Optics, Billerica, MA, USA). In addition, a different set of mice were euthanized and lower leg muscles (soleus, plantaris, gastrocnemius, tibialis, and extensor digitorum longus muscles) were collected with standardized dissection methods, cleaned of excess fat and tendon/connective tissue, and pair weighed on an analytical balance. *Rhesus monkeys*. Monkeys were anesthetized after an overnight fast using either Ketamine (7–10 mg/kg, IM) or Telazol (3–5 mg/kg, IM). Total body scans were acquired with the monkey in a supine position using dual-energy X-ray absorptiometry (DEXA) Lunar Prodigy X-Ray Bone Densitometer (Madison, WI). Data analysis was done with GE encore Software v.12.3. *Humans*. Standing height and weight were measured for each participant. Muscle thickness of the *vastus lateralis* was measured by a portable ultrasound instrument (Mylab25, Esaote, Genova, Italy). Thickness measure was obtained as vertical distance between muscle superficial and deep aponeuroses at an equidistant point from right and left borders of the sagittal image.

### Real Time -quantitative PCR and mtDNA analysis

Total RNA was isolated from frozen muscle samples by Trizol (Invitrogen, NY, USA) and first strand complementary DNA (cDNA) was synthesized using the High Capacity cDNA Reverse Transcription Kit (Applied Biosystems, Austin, TX) following the manufacturer’s instructions. Quantitative real-time PCRs were performed with SYBR® Green PCR master mix to observe expression of desired genes on an ABI Prism 7300 sequence detection kit (Applied Biosystems). Quantification was performed using the ΔΔCT method after normalization to the housekeeper gene glyceraldehyde 3-phosphate dehydrogenase (Gapdh) or β-actin. Each analyzed sample was performed in three replicates. Fidelity of the PCR was determined by melting temperature analysis. Total DNA was extracted with DNeasy blood and tissue kit (QIAGEN, Valencia, CA). In mice, mtDNA was amplified using primers specific for the mitochondrial cytochrome c oxidase subunit 2 (COX2) gene and normalized to genomic DNA by amplification of the ribosomal protein s18 (rps18) nuclear gene. In addition, mtDNA was amplified using primers specific for mitochondrial NADH subunit 1 (rhesus monkeys) and ND1 (humans) gene and normalized to genomic DNA by amplification of β-actin nuclear gene. Oligonucleotides used for real-time RT-quantitative PCR are listed in Table S1.

### Microarray data analysis

RNA was isolated from skeletal muscle of mice, rats, rhesus monkeys, and humans at different ages using Trizol Reagent (Invitrogen, Carlsbad, CA) following the manufacturer’s instructions. RNA was then hybridized to: Mouse, IlluminaMouseRef-8; Rat, Illumina Rat Ref-12; Monkey, Sentrix Human HT-12; Human, Illumina Human HT-12 Expression BeadChips (Illumina, San Diego, CA) following manufacturers protocols. Raw data were subjected to Z normalization to ensure compatibility using the formula: z(raw data) = [ln (raw data)—avg(ln(raw data))]/[stddev(ln (raw data))], where ln is natural logarithm, avg is the average over all genes of an array, stddev is the standard deviation over all genes of an array.^67^ The Z ratio (between treatment A and B) is given by z(A)−z(B)/std dev. Individual genes with Z ratio > 1.5, *p-*value < 0.05, and avg intensity > 0 were considered significantly changed. For PAGE, a list of pathways was obtained from http://www.broad.mit.edu/gsea/msigdb/msigdb_index.html (C2 collection). Our expression data was tested for gene set enrichment using the PAGE method as previously described.^32^ Briefly, for each pathway a Z score was computed as Z (pathway) = (sm mu)*pow(m,0.5)/delta, where mu = mean Z score of all gene symbols on the microarray, delta = standard deviation of Z scores of all gene symbols on the microarray, sm = mean Z score of gene symbols comprising one pathway present on the microarray, and *m* = no of gene symbols in a pathway present on the microarray. For each Z (pathway) a *p-*value was also computed in JMP 6.0 to test for the significance of the Z score obtained. These tools are part of DIANE 6.0 and are described at http://www.grc.nia.nih.gov/branches/rrb/dna/diane_software.pdf. In addition to pathway PAGE analysis described above, statistically significant gene results were analyzed using IPA (www.analysis.ingenuity.com; Ingenuity Systems, Redwood City, CA, USA) to identify the top comparative aging network functions. Gene expression data was deposited in NCBI Gene Expression Omnibus (http://www.ncbi.nlm.nih.gov/geo/) under the accession numberGEO87109according to MIAME compliance.

### Western blotting

Tissues were lysed in radio immunoprecipitation buffer supplemented with ethylenediaminetetraacetic acid and ethylene glycol tetraacetic acid (Boston BioProducts, Ashland, MA) and protease and phosphatase inhibitors (Sigma Aldrich, St Louis, MO). Following centrifugation (14,000 rpm, 30 min at 4 °C) protein concentration was quantified using the Bradford assay method (Bio-Rad, Hercules, CA). Proteins were separated by sodium dodecyl sulfate polyacrylamide gel electrophoresis under reducing conditions and then transferred to nitrocellulose membranes. Western blots were performed according to standard methods. Membranes were blocked in 5% nonfat milk, incubated with the antibody of interest followed by incubation in a secondary antibody conjugated with horseradish peroxidase. Immunoreactive bands were visualized using chemiluminescent (ECL) Plus western blotting detection system (GE Healthcare, Pascataway, NJ), Immobilon Western Chemiluminescent HRP Substrate (Millipore, Billerica, MA), or SuperSignal West Femto Maximum Sensitivity Substrate (Thermo Scientific, Waltham, MA). The quantification was performed by volume densitometry using ImageJ software and normalized to staining with Ponceau S solution or anti-β-tubulin (both from Sigma-Aldrich, St. Louis, MO), which served to verify equal protein loading. The primary antibodies used in this study were directed against Mfn1, Mfn2, Drp1, and Fis1 (Santa Cruz Biotechnology, Inc., Santa Cruz, CA), PINK1 (Novus Biologicals, Littleton, CO), Parkin, pNF-κB, AKT, pAKT (Ser473), FOXO, pFOXO, GSK-3β, and pGSK-3β (Cell Signaling Technology, Inc., Danvers, MA), BNIP-3 (Sigma Aldrich), NIX, COX2 (Abcam Inc., Cambridge, MA), 4HNE (EMD Calbiochem, La Jolla, CA) and NF-κB (Epitomics, Inc., Burlingame, CA), which were generally used at the manufacturer’s recommended dilution. For detection of protein NF-κB levels in humans, rabbit monoclonal C22B4 anti-NF-κB (Cell Signaling) was used. Protein carbonylation was measured using an oxyblot protein oxidation kit (Millipore, Billerica, MA) according to the manufacturer’s instructions.

### Sample preparation and BNE

Preparation of crude mitochondrial membranes and solubilization of mitochondrial membrane protein complexes were performed as previously described.^68^ Briefly, skeletal muscle samples from rats, rhesus monkeys and humans were weighed and finely minced with scissors. The minced tissue was homogenized in 250 mM Sucrose, 10 mM Tris-Cl pH 7.5, 2 mM 6-aminohexanoic acid using a pre-cooled motor-driven, tightly fitting glass/Teflon Potter-Elvehjem homogenizer. Aliquots corresponding to 30 mg wet weight were centrifuged 10 min at 10,000×*g*. Pellets were frozen in liquid nitrogen and stored at −80 °C. In order to solubilize protein complexes, aliquots were resuspended in 50 mM NaCl, 50 mM imidazole pH 7, 2 mM 6-aminohexanoic acid and solubilized by adding 10 µl digitonin (20% stock in water (w/v)). Samples were centrifuged for 20 min at 22,000×*g* and the protein content of the supernatant was measured. Equal amounts of protein/lane (50 µg/sample for Complex I (CI) in-gel activity stain and native blot; 75 µg for Coomassie stain) were mixed with a 5% Coomassie blue G-250 dye stock suspension to obtain a detergent/dye ratio of 8/1. Blue native electrophoresis was performed as described.^68^ Due to the limited sample size of the human samples BNE was performed in mini format as previously described.^69^ Equal amounts of protein/lane (10 µg/sample for CI in-gel activity stain and native blot; 15 µg for Coomassie stain) were loaded.

### Two-dimensional (2D) BN/SDS page

Following separation of mitochondrial OXPHOS complexes by BNE, subunit composition was studied using 2D BN/sodium dodecyl sulfate-polyacryl amide gel electrophoresis as described in ref. 68. Briefly, one-dimensional (1D) BN-strips were soaked in 1% SDS for a few minutes and a SDS-gel was casted below. 1D strips were pushed on top of the 2D gel and SDS electrophoresis was performed at room temperature. Gels were silver-stained.^70^

### 1D BN western blotting

Native protein complexes were transferred to a Polyvinylidene difluoride membrane using an electroblotting buffer composed of 50 mM Tricine, 7.5 mM imidaziol for 3–4 h at room temperature, voltage set to 20 V and current limited to 0.5 mA/cm^2^ (ref. 68). Blots were sequentially decorated with a monoclonal mouse antibody against a complex I subunit NDUFB8 (Invitrogen) and polyclonal rabbit antiserum against subunits of OXPHOS complexes III, V, IV, and II (in that order). Immunoreactive bands were visualized by ECL chemiluminescence and detected by a ChemiDoc XRS device (Bio-Rad). Densitometric quantification of samples from rats and rhesus monkeys was performed by Quantity One software package (Bio-Rad). Densitometric quantification of human samples was performed by AIDA image analyzer software package (Raytest).

### Coomassie stain of 1D BN-gels

Gels were fixed in 50% methanol, 10% acetic acid and stained with 0.025% Coomassie G250 (Serva) in 10% acetic acid. Following destaining in 10% acetic acid gels were scanned by an office flatbed scanner (Epson perfection 2400 PHOTO) for documentation.

### Complex I *in-gel* catalytic activity stains

In gel activity stain was used to quantify complex I containing supercomplexes isolated by 1D BNE. The *in-gel* NADH: nitrotetrazolium blue (NTB) reductase assay followed essentially the protocols described by Zerbetto et al.^71^ with some modifications according to Wittig et al.^72^. Briefly, complex I *in-gel* activity stain was done in 1D BN-gels directly after electrophoresis. 1D BNE gels were incubated in buffer containing 5 mM Tris/HCl, pH 7.4, 0.1 mg/ml NADH and 2.5 mg/ml NTB until the purple colour was developed (approx. 30 min). Reaction was stopped with 50% methanol, 10% acetic acid fixing solution for several hours to remove Coomassie. Gels were placed into water and scanned for further densitometric analysis by the Quantity One Software (Bio-Rad).

### Citrate synthase activity

Citrate synthase activity was determined in approximately 20 µg of protein lysates following the method described by Bernier et al.^73^ Citrate synthase were determined by spectrophotometric methods. Results were expressed in nmol/mg protein/min.

### Cardiolipin

SS and IMF mitochondria were isolated as previously described.^74^ The isolated pellets were suspended in 100 ul of Reagent C from the Mitochondria Isolation Kit (Abcam Inc., Cambridge, MA) and frozen at −80 °C. Isolated mitochondria were extracted using a previously described method.^75^ Briefly, to 80 μl of the suspension, 320 μl of water was added and mixed. Ten microliter of 1 mg/ml of myristoyl-cardiolipin was added as internal standard. Two milliliter of a chloroform:methanol (2:1) mixture was added and the aqueous layer was removed. Four hundred microliter of water was added twice to the organic phase and removed. The samples were then subjected to silica gel chromatography and the lipid classes were eluted in solvents with increasing polarity: (95:1 (Isooctane: EtOAc); 20:1 (Isooctane:EtOAc); 75:25 Isooctaine:EtOAc; 75:25:2 (Isooctane:EtOAc:glacial acetic acid); 75:25:2 (Isooctane:EtOAc: glacial acetic acid). The phospholipids were eluted in the final step with 4 ml of methanol, stream dried, and then reconstituted again in 250 ml of methanol. MS/MS analysis was performed using a triple quadrupole mass spectrometer model API 4000 system from Applied Biosystems/MDS Sciex equipped with Turbo Ion Spray® (TIS) (Applied Biosystems, Foster City, CA, USA). The data was acquired and analyzed using Analyst version 1.4.2 (Applied Biosystems). Negative electrospray ionization data were acquired using multiple reaction monitoring (MRM). The TIS instrumental source settings for temperature, curtain gas, ion source gas 1 (nebulizer), ion source gas 2 (turbo ion spray), and ion spray voltage were 500 °C, 20 psi, 60 psi, 60 psi, and −4500 V, respectively. The TIS compound parameter settings for declustering potential, entrance potential, and collision cell exit potential were −110—10 V, and −6 V, respectively. The collision energy setting was −50 V for cardiolipin molecular species and −75 V for myristoylcardiolipin. The MRM transitions were (C18:2)4-CL (1448/695.0); (C18:2)3(C18:1)-CL(1450/695.0), and myristoyl-CL (1240/227.3). The chromatographic experiments were carried out on a Shimadzu Prominence HPLC system (Shimadzu, Columbia, MD, USA) following a previously described protocol, with slight modifications.^75^ Briefly, the samples were introduced to the analytical column using Shimadzu SIL-20A autosampler and maintained at 4 °C in the autosampler tray, and injections of 30 μl were made with a column temperature of 35 °C. The separation of (C18:2)4-CL, (C18:2)3(C18:1)-CL and (C18:2)2(C18:1)2-CL was accomplished using a Symmetry C18 column (150 × 3.9 mm ID, 5 μ). The mobile phase consisted of 90:10 acetonitrile:water containing 0.5% of trimethylamine and 0.5% of glacial acetic acid as Component A and 90:10 isopropanol:water containing 0.5% of trimethylamine and 0.5% of glacial acetic acid Component B. A linear gradient was run as follows:0 min 0% B; 5 min 0% B; 70 min 100% B; 80 min 100% B; 85 min 50% B at 0.4 ml/min. The total run time was 90 min per sample.

### ATP analysis

ATP content was measured with a commercial kit according to the manufacturer’s instructions (Abcam Inc., Cambridge, MA).

### Electron microscopy

For TEM analysis, gastrocnemius, and vastus lateralis samples from mice and rhesus monkey, respectively, were removed and placed directly into a fixative solution consisting of 2.5% glutaraldehyde and 3% paraformaldehyde in 0.1 M sodium cacodylate buffer (Electron Microscopy Sciences, Hatfield PA). The tissues were post fixed in 1% osmium tetroxide for 1 h at 4 °C in the same buffer, dehydrated and then embedded in Embed 812 resin (Electron Microscopy Sciences, Hatfield PA), after which tissue samples were transferred to pure resin for 24 h. Blocks were formed in fresh resin contained in silicon molds, and the resin was allowed to polymerize for 48 h at 65 °C. Ultrathin sections were obtained and stained with uranyl acetate and lead citrate, and then examined on a Philips CM-10 electron microscope (ServicioCentralizado de Apoyo a la Investigación, SCAI, University of Córdoba, Spain) at 25,000x magnification by a blinded investigator. Ten digital images per sample were analyzed using NIH Image J (NIH, USA) software for measurement of mitochondrial area and mitochondrial circularity.

### Statistics

Data are expressed as means ± standard error of the mean (SEM). Between-group comparisons were analyzed using the Student’s *t*-tests are Mann–Whitney *U-*test when normality assumption was not obtained. Analyses were performed using Excel 2010 (Microsoft Corp., Redmond, WA). A *p-*value of ≤0.05 was considered statistically significant.

## Electronic supplementary material

Supplementary Figure 1

Supplementary Figure 2

Supplemental Figure 3

Supplementary Figure 4

Supplementary Figure 5

Supplementary Figure 6

Supplemental Figure 7

Supplemental Figure 8

Supplemental Table 1

Supplemental Table 2

Supplemental Table 3

Supplemental Table 4

Supplemental Table 5

Supplementary file

## Electronic supplementary material

Supplementary Information accompanies the paper on the *npj Aging and Mechanisms of Disease* website (doi:10.1038/s41514-017-0009-8).
