# Supplementary figures and images for: Conserved and species-specific molecular denominators in mammalian skeletal muscle aging
Source: NPJ Aging Mech Dis. 2017 May 5;3:8. doi: 10.1038/s41514-017-0009-8 (PMC5460213; doi:10.1038/s41514-017-0009-8)

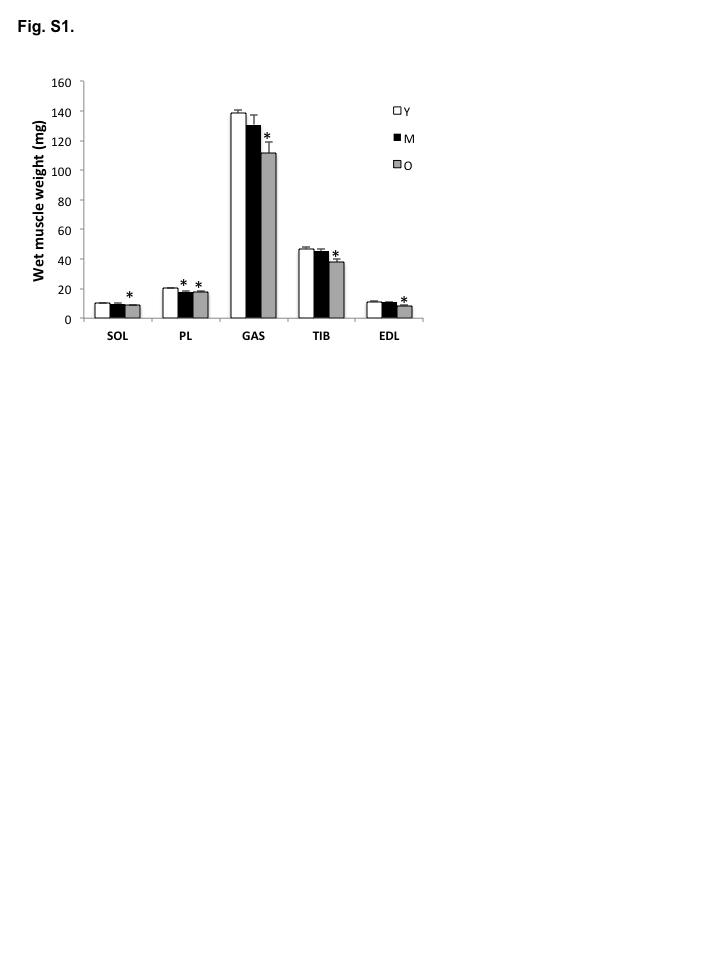

Supplement: Supplementary file 1 — Supplementary Figure 1 [file 41514_2017_9_MOESM1_ESM.tif]

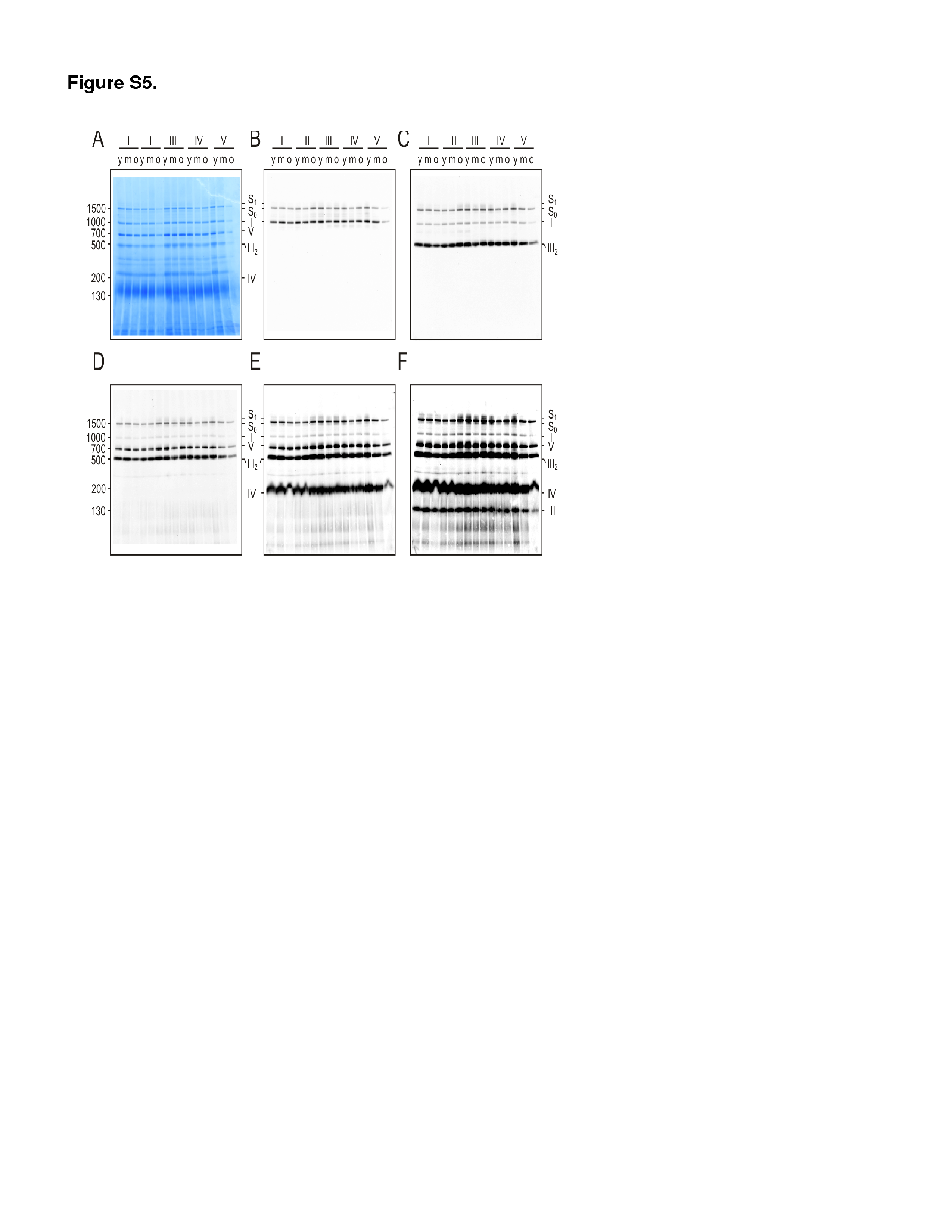

Supplement: Supplementary file 5 — Supplementary Figure 5 [file 41514_2017_9_MOESM5_ESM.tif]

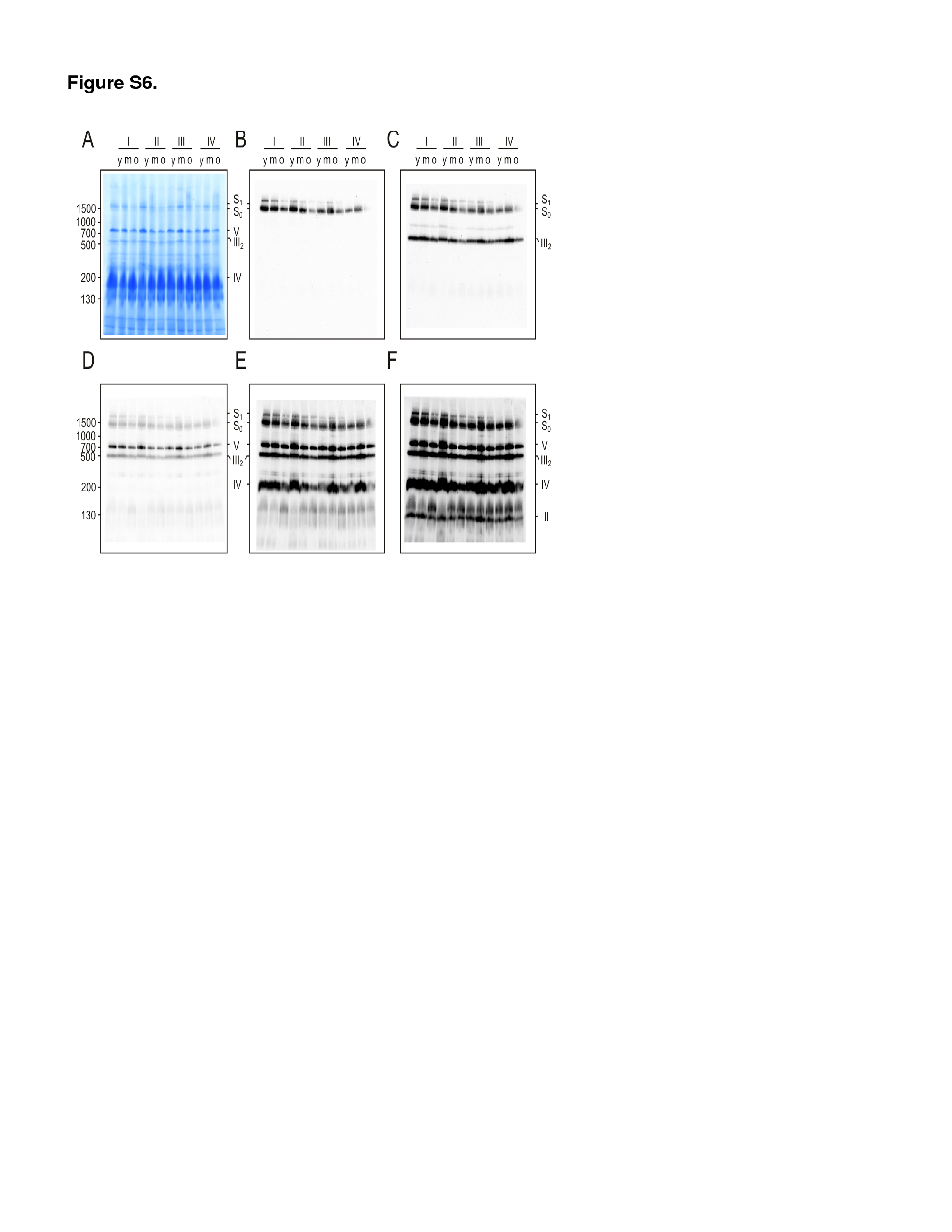

Supplement: Supplementary file 6 — Supplementary Figure 6 [file 41514_2017_9_MOESM6_ESM.tif]

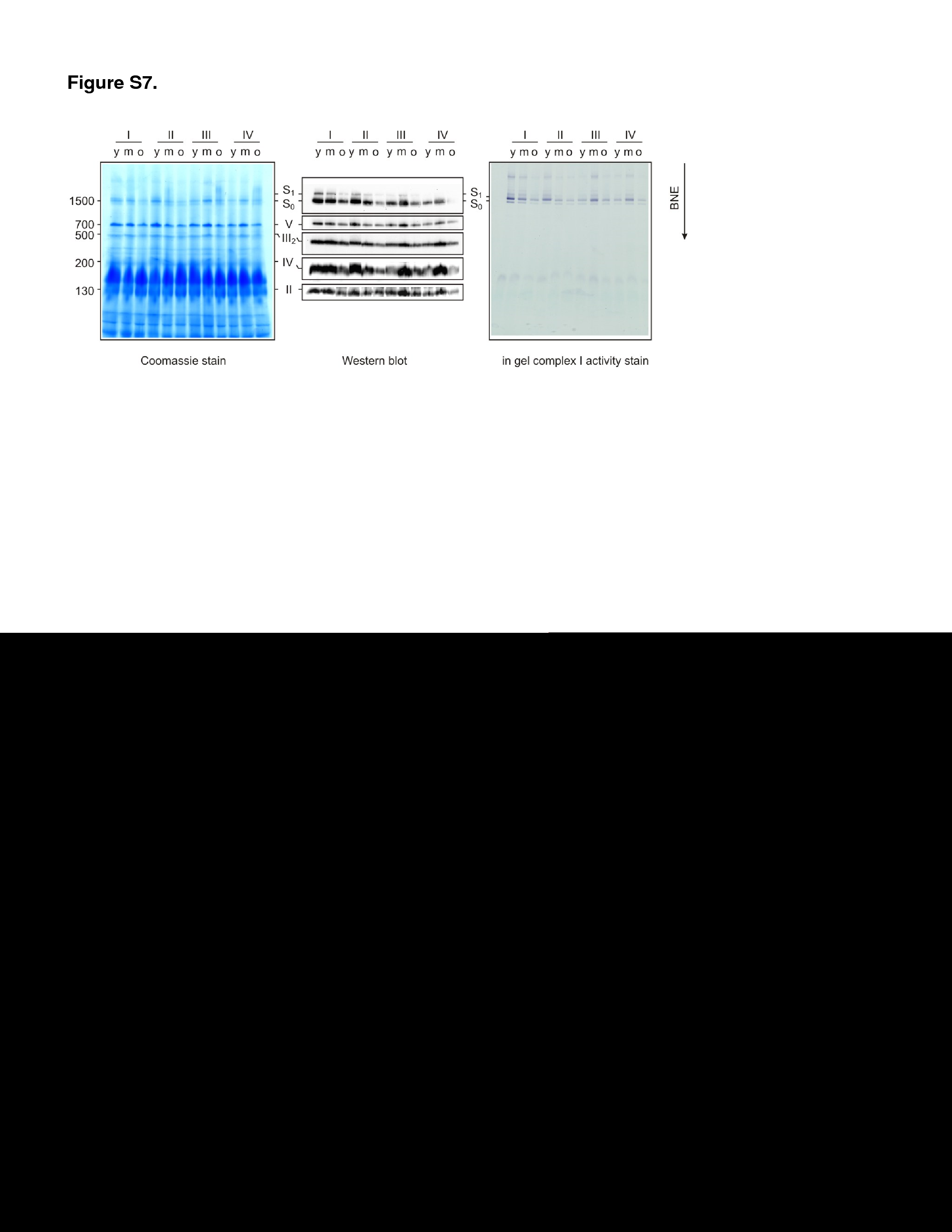

Supplement: Supplementary file 7 — Supplemental Figure 7 [file 41514_2017_9_MOESM7_ESM.tif]

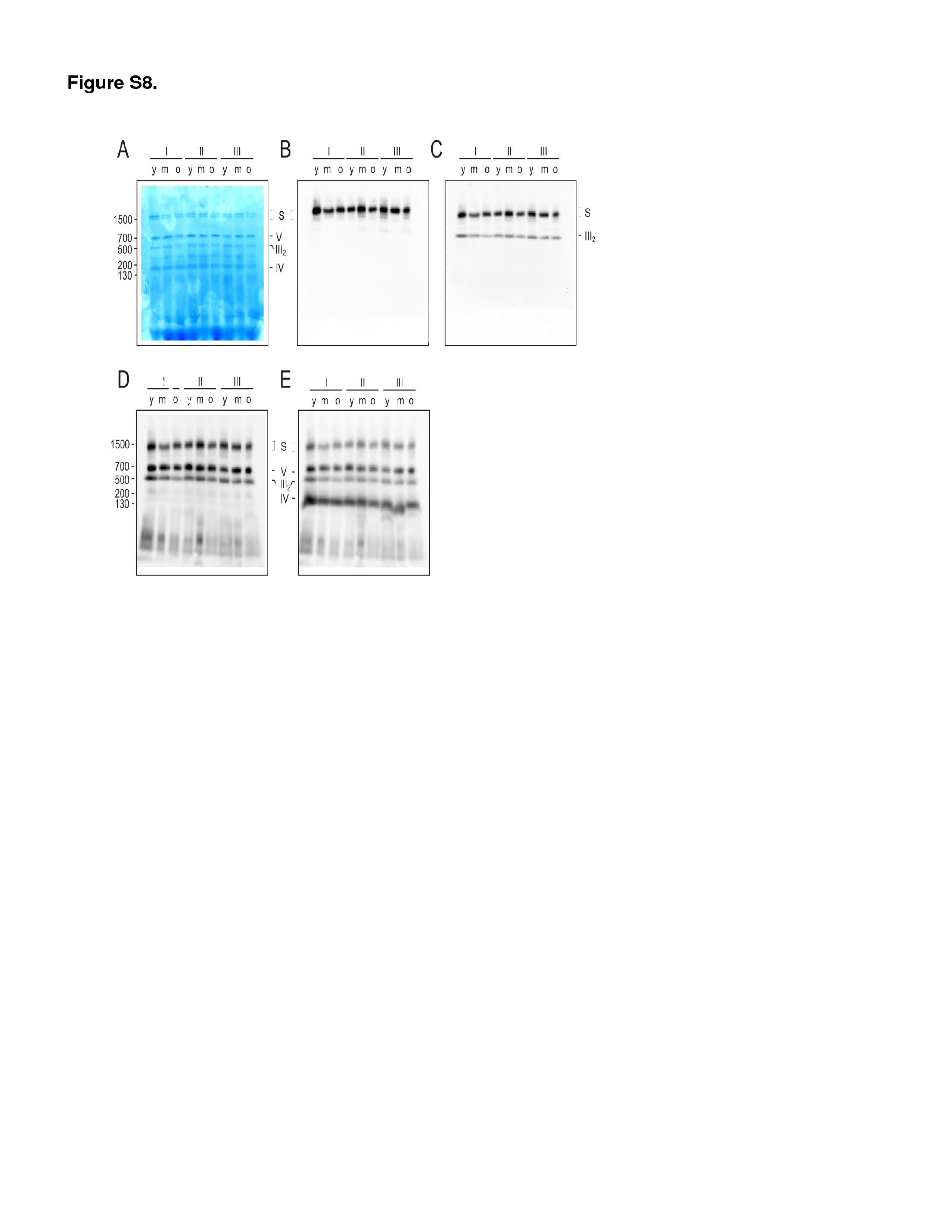

Supplement: Supplementary file 8 — Supplemental Figure 8 [file 41514_2017_9_MOESM8_ESM.tif]
