# Supplementary material for: Conserved and species-specific molecular denominators in mammalian skeletal muscle aging
Source: NPJ Aging Mech Dis. 2017 May 5;3:8. doi: 10.1038/s41514-017-0009-8 (PMC5460213; doi:10.1038/s41514-017-0009-8)
Supplement: Supplementary file 2 — Supplementary Figure 2 [file 41514_2017_9_MOESM2_ESM.pdf]

Mouse_ O-Y Mitochondrial Dysfunction

/sfunction

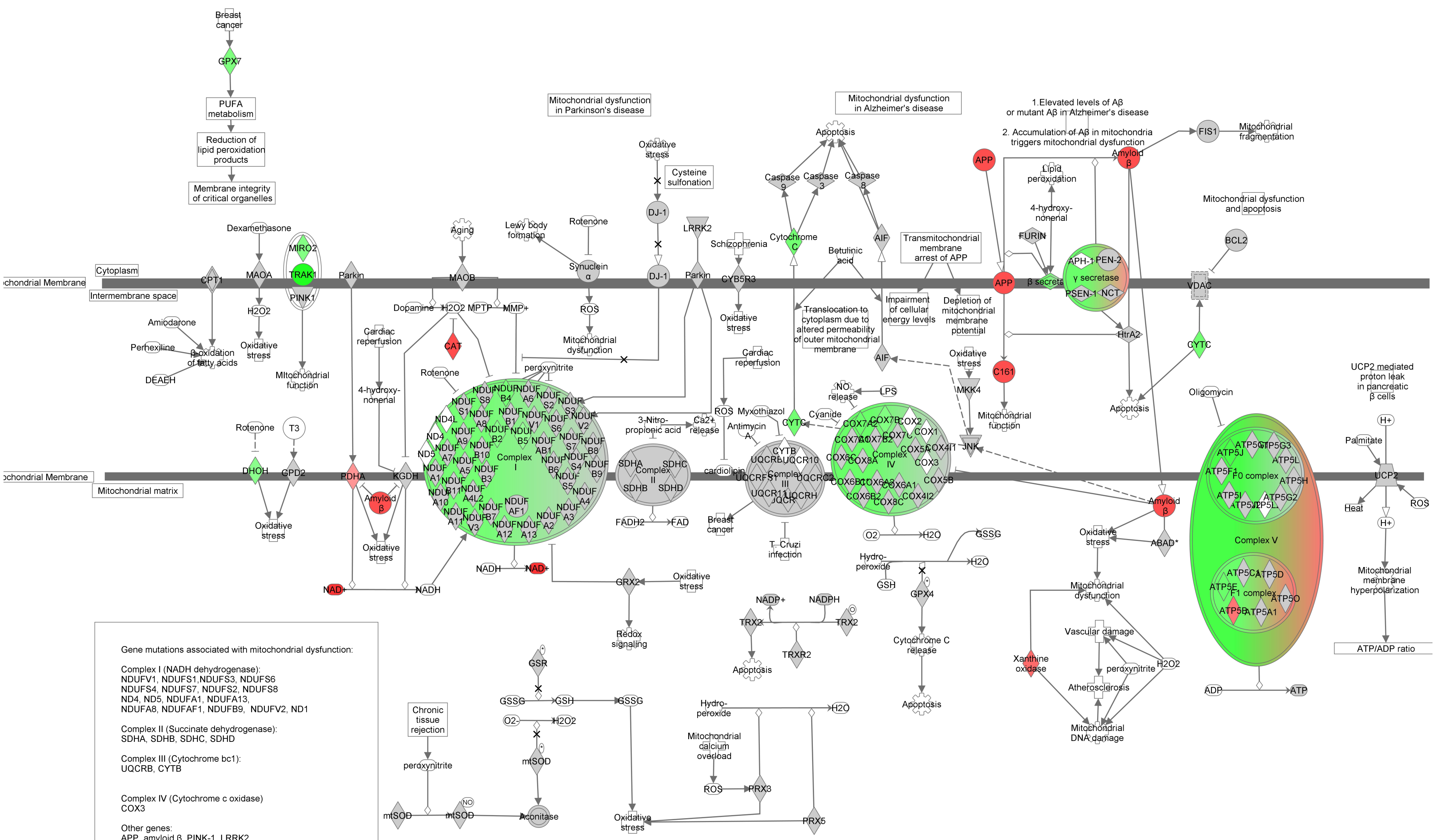

Gene mutations associated with mitochondrial dysfunction:

Complex I (NADH dehydrogenase):
NDUFV1, NDUFS1,NDUFS3, NDUFS6
NDUFS4, NDUFS7, NDUFS2, NDUFS8
ND4, ND5, NDUFA1, NDUFA13,
NDUFA8, NDUFAF1, NDUFB9, NDUFV2, ND1

Complex II (Succinate dehydrogenase):
SDHA, SDHB, SDHC, SDHD

Complex III (Cytochrome bc1):
UQCRB, CYTB

Complex IV (Cytochrome c oxidase)
COX3

Other genes:
APP, amyloid β, PINK-1, LRRK2
presenilin-1, α-synuclein, Parkin, DJ-1

N. All rights reserved.

unction

Mouse_ O-M Mitochondrial Dysfunction

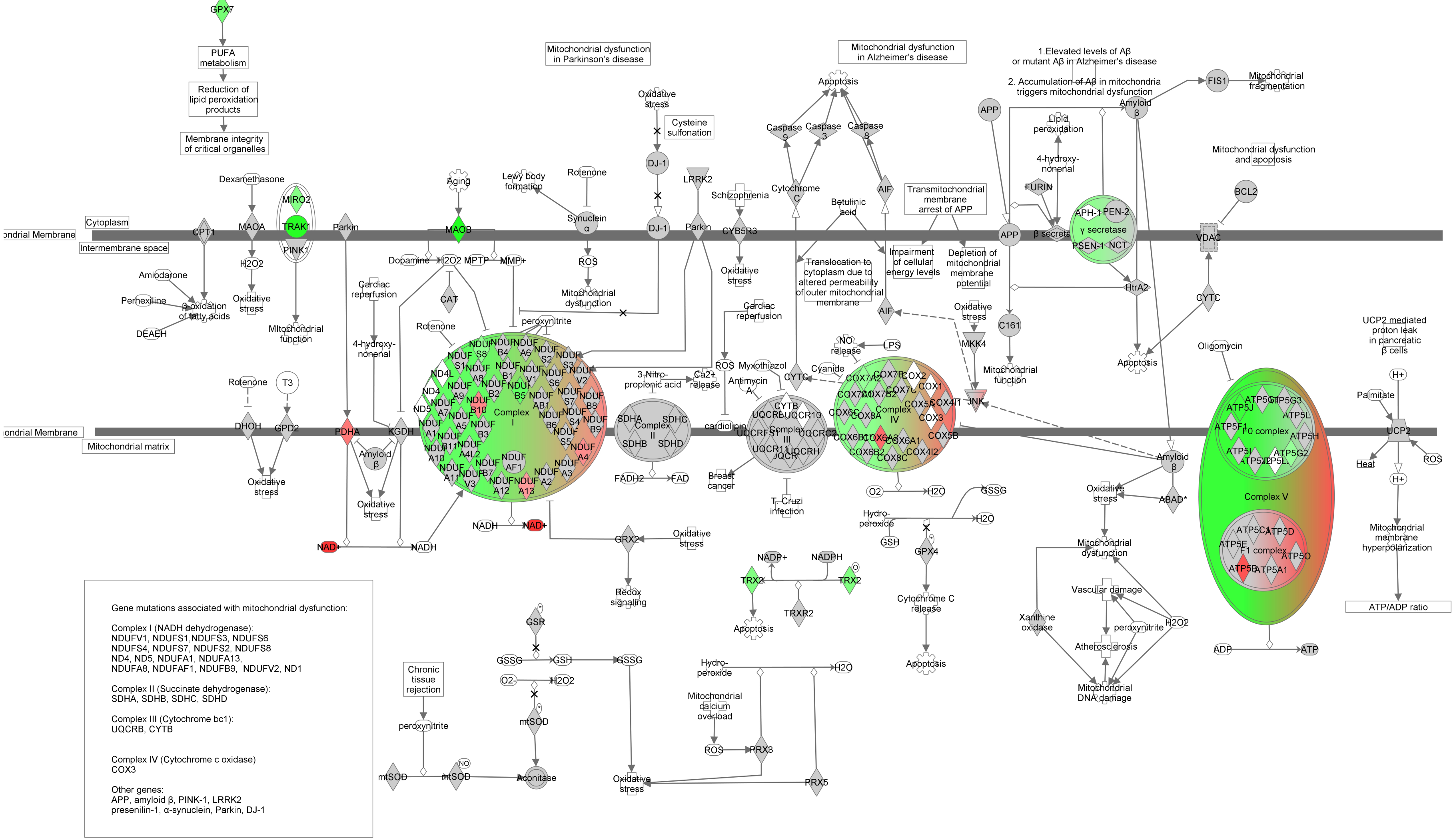

Gene mutations associated with mitochondrial dysfunction:

Complex I (NADH dehydrogenase):
NDUFV1, NDUFS1,NDUFS3, NDUFS6
NDUFS4, NDUFS7, NDUFS2, NDUFS8
ND4, ND5, NDUFA1, NDUFA13,
NDUFA8, NDUFAF1, NDUFB9, NDUFV2, ND1

Complex II (Succinate dehydrogenase):
SDHA, SDHB, SDHC, SDHD

Complex III (Cytochrome bc1):
UQCRB, CYTB

Complex IV (Cytochrome c oxidase)
COX3

Other genes:
APP, amyloid β, PINK-1, LRRK2
presenilin-1, α-synuclein, Parkin, DJ-1

ll rights reserved.

Mouse_ M-Y Mitochondrial Dysfunction

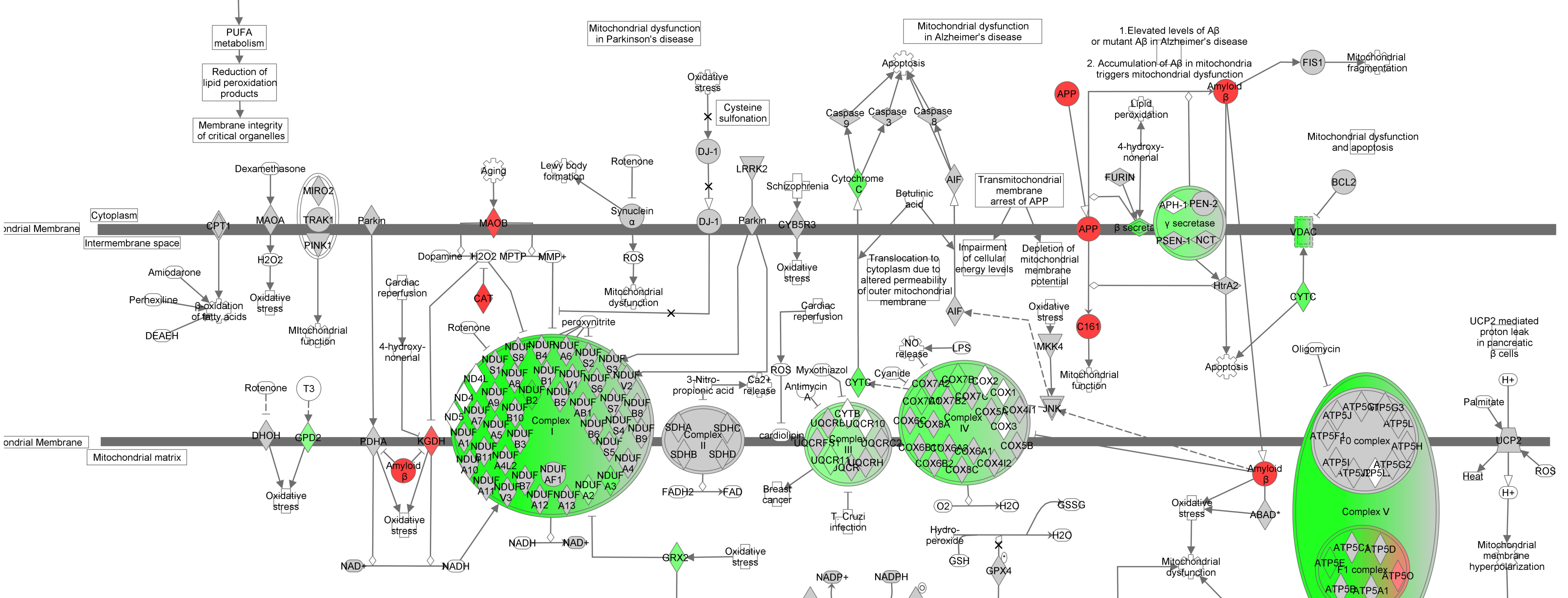

Gene mutations associated with mitochondrial dysfunction:

Complex I (NADH dehydrogenase):
NDUFV1, NDUFS1,NDUFS3, NDUFS6
NDUFS4, NDUFS7, NDUFS2, NDUFS8
ND4, ND5, NDUFA1, NDUFA13,
NDUFA8, NDUFAF1, NDUFB9, NDUFV2, ND1

Complex II (Succinate dehydrogenase):
SDHA, SDHB, SDHC, SDHD

Complex III (Cytochrome bc1):
UQCRB, CYTB

Complex IV (Cytochrome c oxidase)
COX3

Other genes:
APP, amyloid β, PINK-1, LRRK2
presenilin-1, α-synuclein, Parkin, DJ-1

l rights reserved.

Rat_ O-Y Mitochondrial Dysfunction

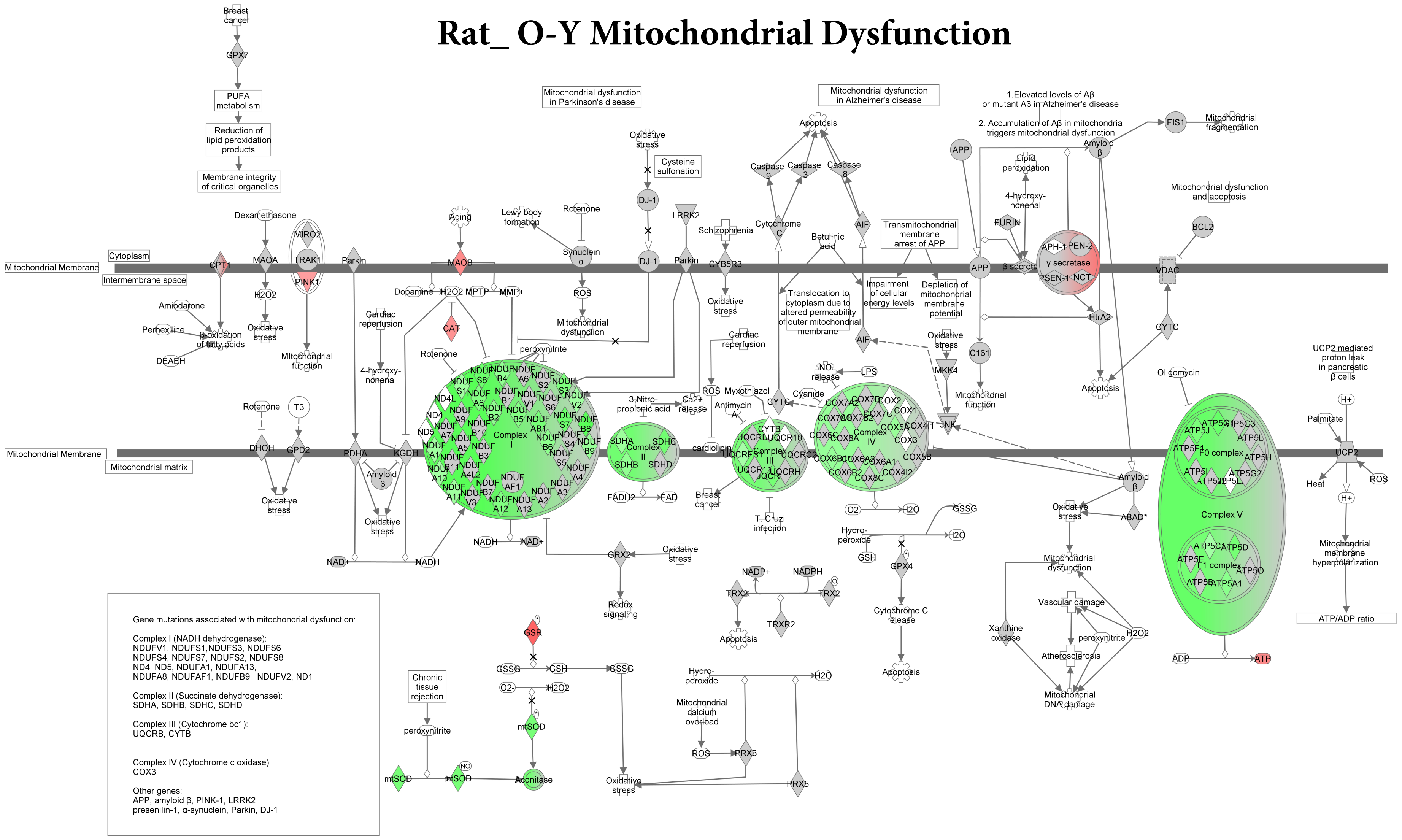

AGEN. All rights reserved.

Rat_ M-Y Mitochondrial Dysfunction

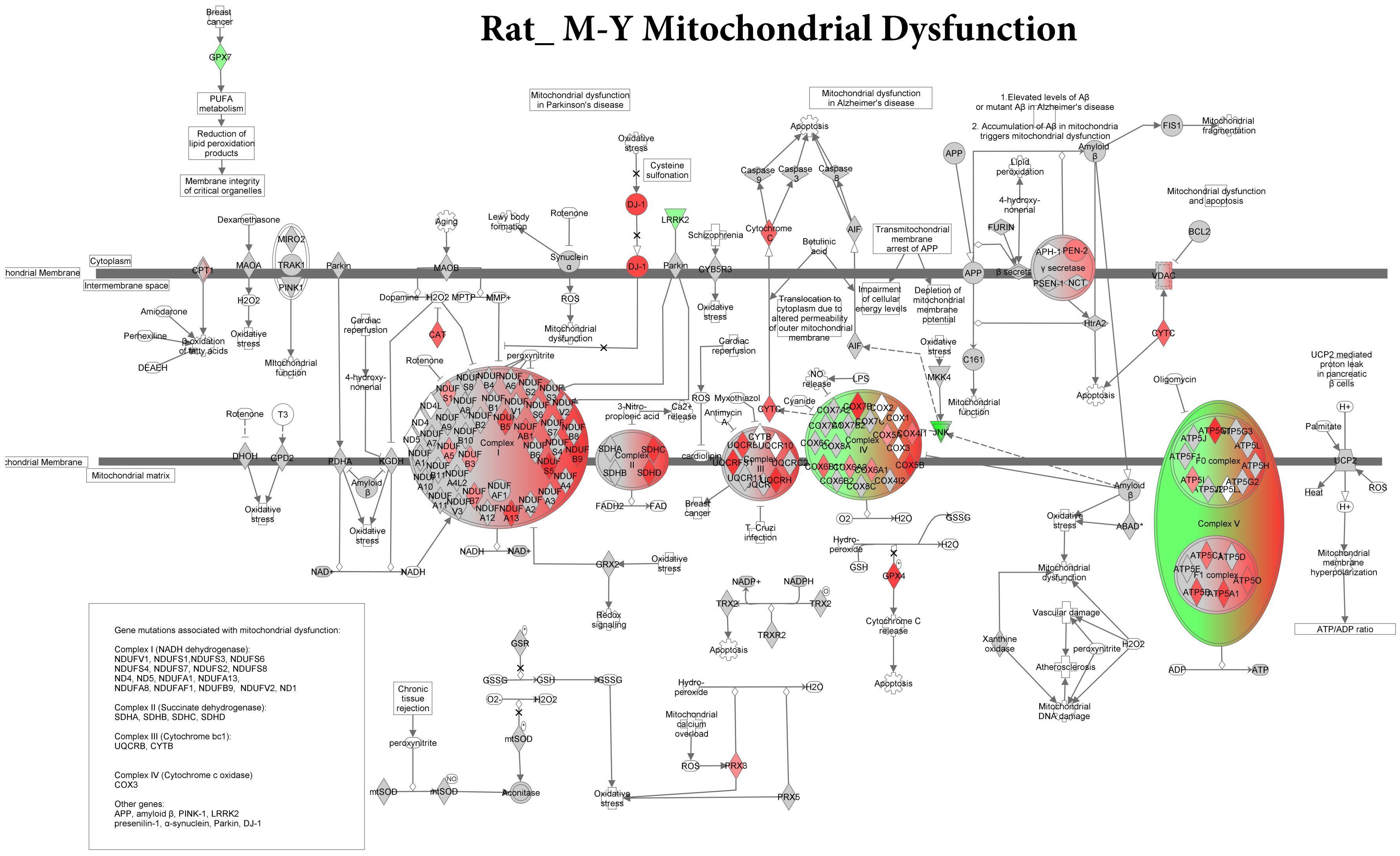

Gene mutations associated with mitochondrial dysfunction:

Complex I (NADH dehydrogenase):
NDUFV1, NDUFS1,NDUFS3, NDUFS6
NDUFS4, NDUFS7, NDUFS2, NDUFS8
ND4, ND5, NDUFA1, NDUFA13,
NDUFA8, NDUFAF1, NDUFB9, NDUFV2, ND1

Complex II (Succinate dehydrogenase):
SDHA, SDHB, SDHC, SDHD

Complex III (Cytochrome bc1):
UQCRB, CYTB

Complex IV (Cytochrome c oxidase)
COX3

Other genes:
APP, amyloid β, PINK-1, LRRK2
presenilin-1, α-synuclein, Parkin, DJ-1

All rights reserved.

ction

Rat_ O-M Mitochondrial Dysfunction

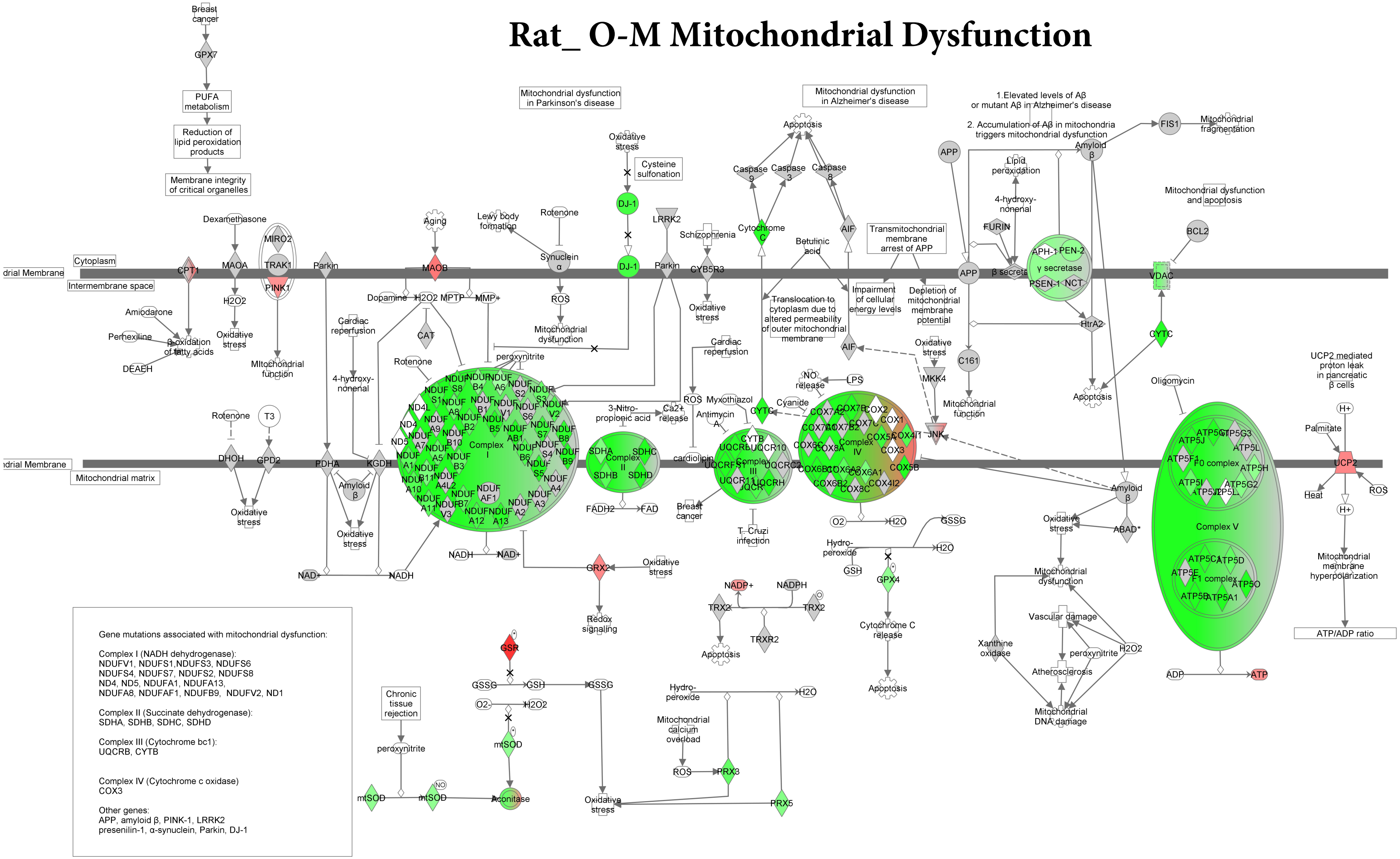

Gene mutations associated with mitochondrial dysfunction:

Complex I (NADH dehydrogenase):
NDUFV1, NDUFS1,NDUFS3, NDUFS6
NDUFS4, NDUFS7, NDUFS2, NDUFS8
ND4, ND5, NDUFA1, NDUFA13,
NDUFA8, NDUFAF1, NDUFB9, NDUFV2, ND1

Complex II (Succinate dehydrogenase):
SDHA, SDHB, SDHC, SDHD

Complex III (Cytochrome bc1):
UQCRB, CYTB

Complex IV (Cytochrome c oxidase)
COX3

Other genes:
APP, amyloid β, PINK-1, LRRK2
presenilin-1, α-synuclein, Parkin, DJ-1

ghts reserved.

Rhesus_ O-Y Mitochondrial Dysfunction

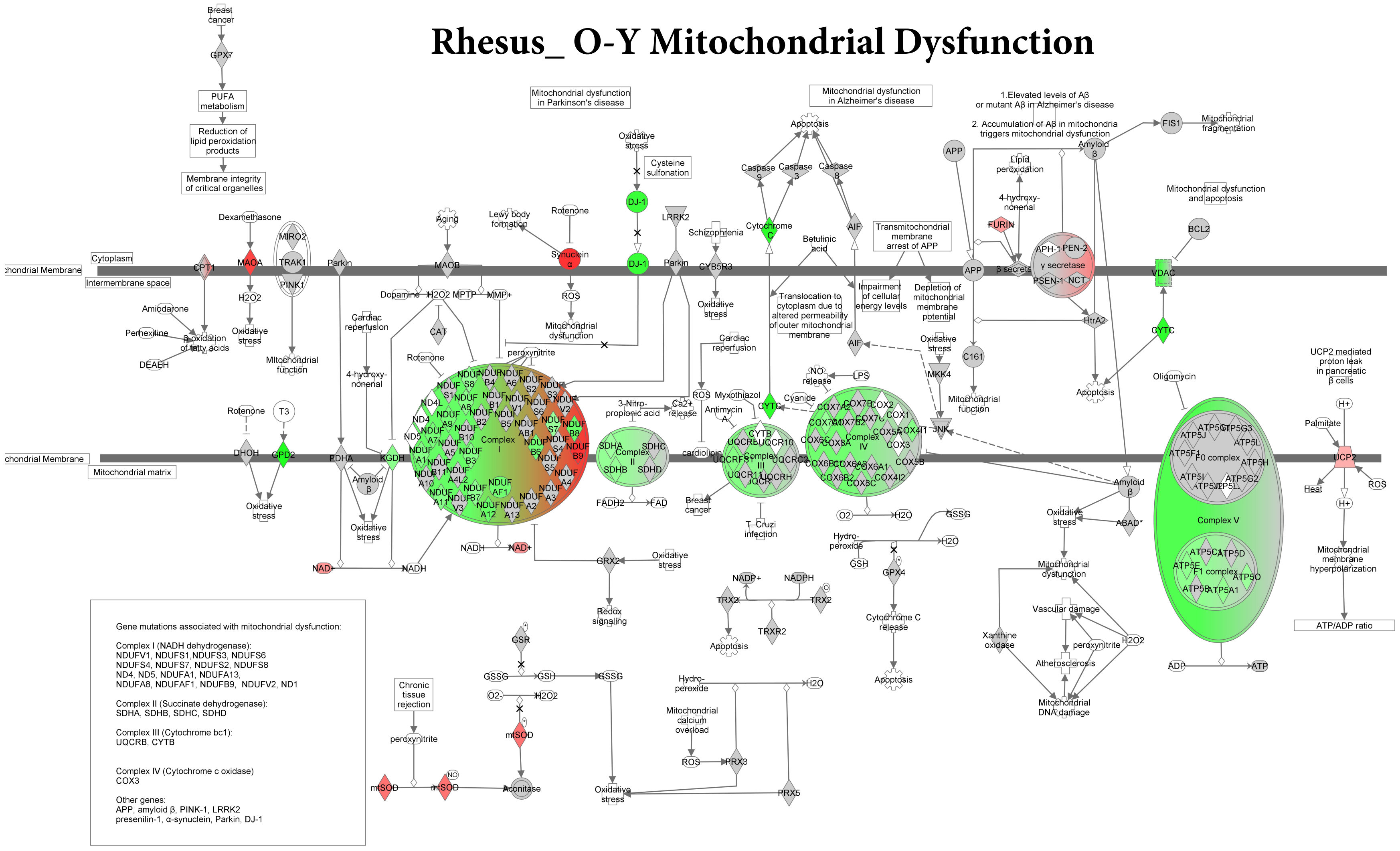

. All rights reserved.

Rhesus_ M-Y Mitochondrial Dysfunction

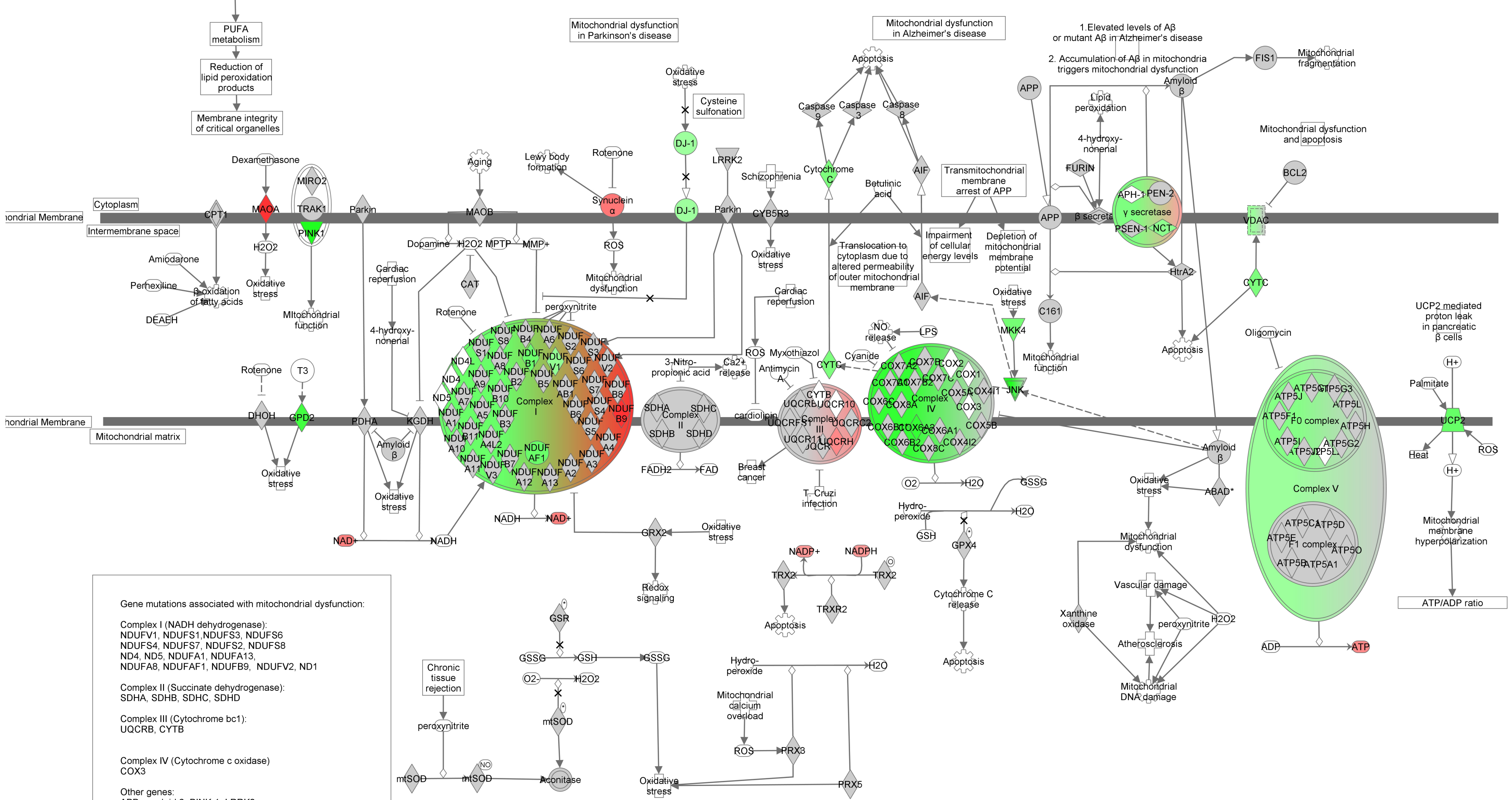

Gene mutations associated with mitochondrial dysfunction:

Complex I (NADH dehydrogenase):
NDUFV1, NDUFS1,NDUFS3, NDUFS6
NDUFS4, NDUFS7, NDUFS2, NDUFS8
ND4, ND5, NDUFA1, NDUFA13,
NDUFA8, NDUFAF1, NDUFB9, NDUFV2, ND1

Complex II (Succinate dehydrogenase):
SDHA, SDHB, SDHC, SDHD

Complex III (Cytochrome bc1):
UQCRB, CYTB

Complex IV (Cytochrome c oxidase)
COX3

Other genes:
APP, amyloid β, PINK-1, LRRK2
presenilin-1, α-synuclein, Parkin, DJ-1

All rights reserved.

ction

Rhesus_ O-M Mitochondrial Dysfunction

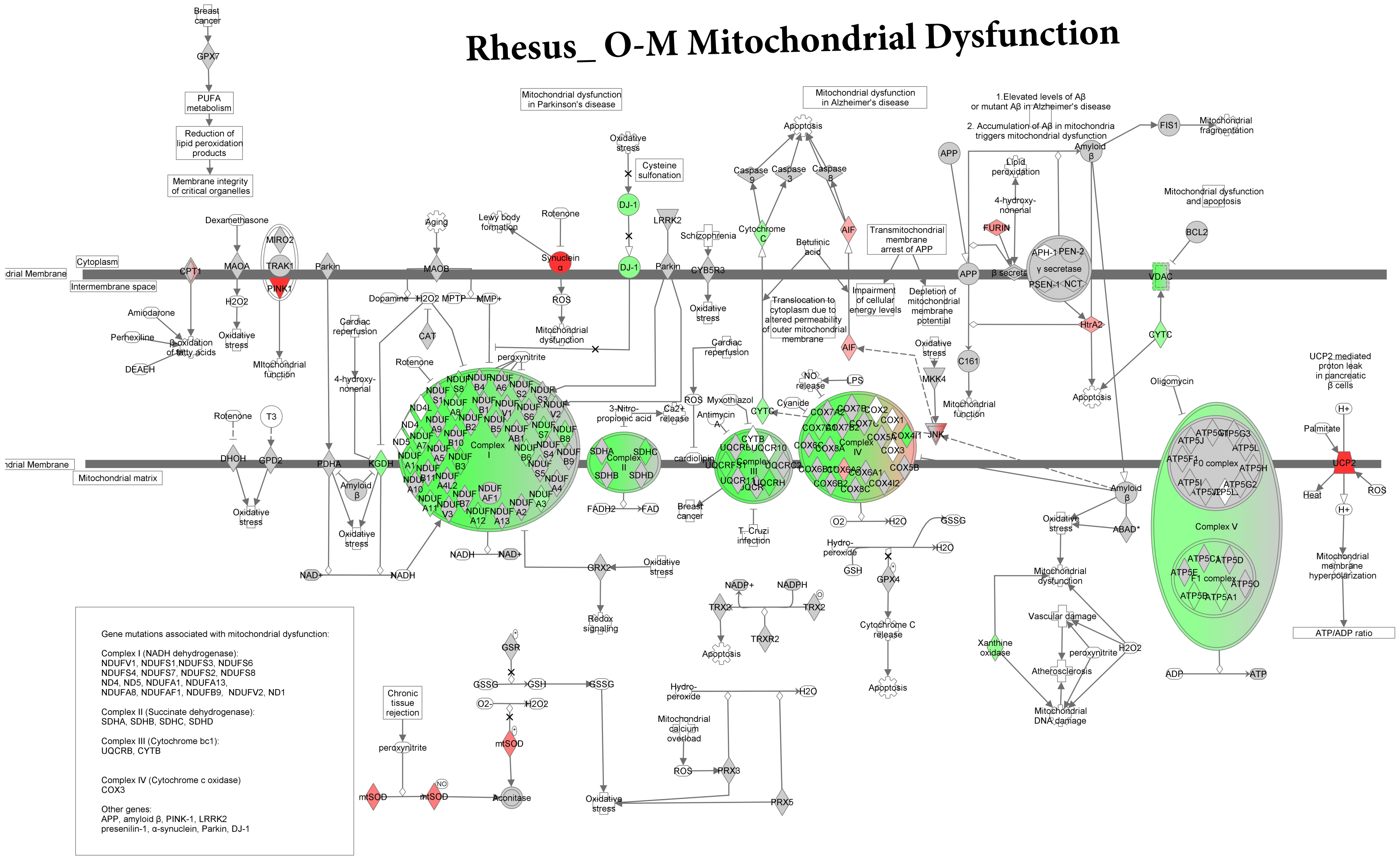

Gene mutations associated with mitochondrial dysfunction:

Complex I (NADH dehydrogenase):
NDUFV1, NDUFS1,NDUFS3, NDUFS6
NDUFS4, NDUFS7, NDUFS2, NDUFS8
ND4, ND5, NDUFA1, NDUFA13,
NDUFA8, NDUFAF1, NDUFB9, NDUFV2, ND1

Complex II (Succinate dehydrogenase):
SDHA, SDHB, SDHC, SDHD

Complex III (Cytochrome bc1):
UQCRB, CYTB

Complex IV (Cytochrome c oxidase)
COX3

Other genes:
APP, amyloid β, PINK-1, LRRK2
presenilin-1, α-synuclein, Parkin, DJ-1

ights reserved.

Human_ O-Y Mitochondrial Dysfunction

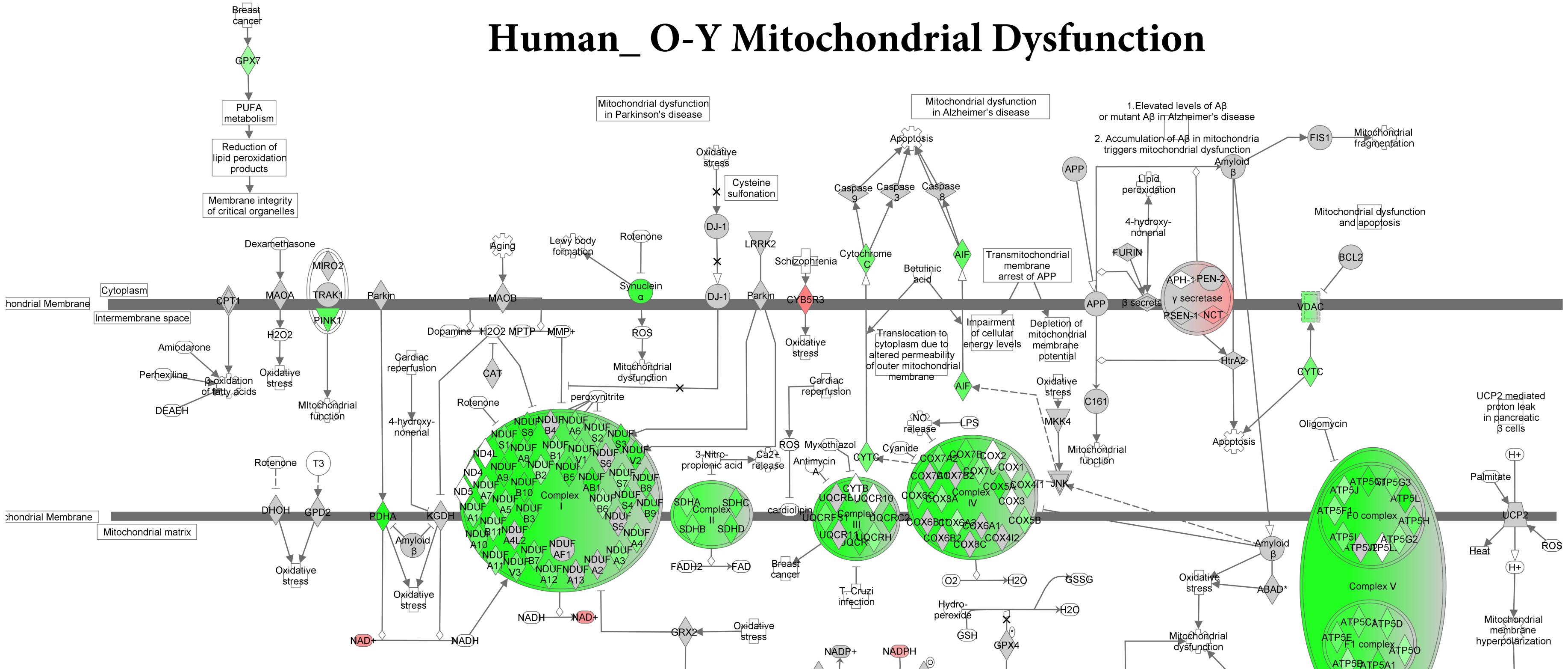

Gene mutations associated with mitochondrial dysfunction:

Complex I (NADH dehydrogenase):
NDUFV1, NDUFS1,NDUFS3, NDUFS6
NDUFS4, NDUFS7, NDUFS2, NDUFS8
ND4, ND5, NDUFA1, NDUFA13,
NDUFA8, NDUFAF1, NDUFB9, NDUFV2, ND1

Complex II (Succinate dehydrogenase):
SDHA, SDHB, SDHC, SDHD

Complex III (Cytochrome bc1):
UQCRB, CYTB

Complex IV (Cytochrome c oxidase)
COX3

Other genes:
APP, amyloid β, PINK-1, LRRK2
presenilin-1, α-synuclein, Parkin, DJ-1

All rights reserved.

Human_ M-Y Mitochondrial Dysfunction

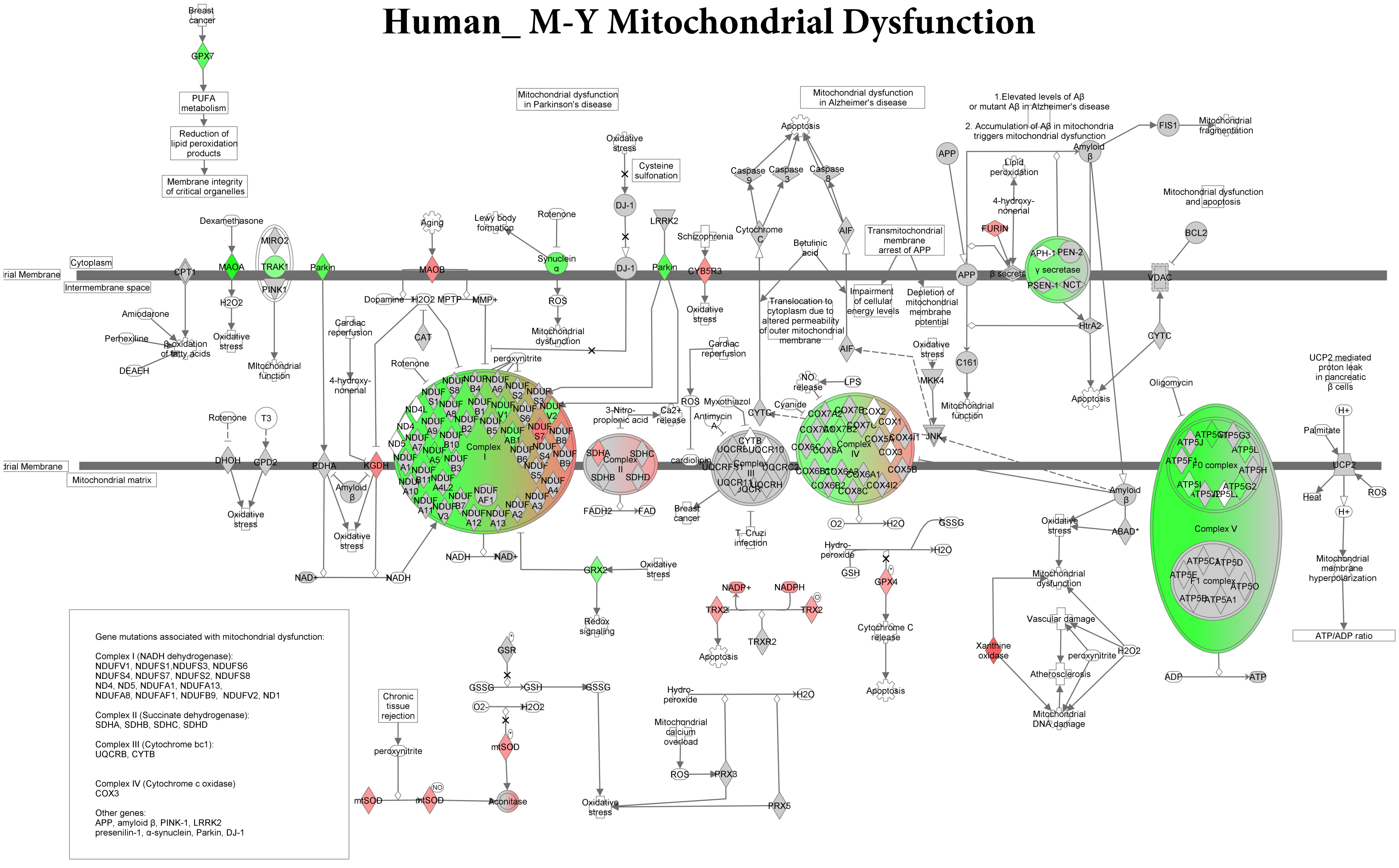

Gene mutations associated with mitochondrial dysfunction:

Complex I (NADH dehydrogenase):
NDUFV1, NDUFS1,NDUFS3, NDUFS6
NDUFS4, NDUFS7, NDUFS2, NDUFS8
ND4, ND5, NDUFA1, NDUFA13,
NDUFA8, NDUFAF1, NDUFB9, NDUFV2, ND1

Complex II (Succinate dehydrogenase):
SDHA, SDHB, SDHC, SDHD

Complex III (Cytochrome bc1):
UQCRB, CYTB

Complex IV (Cytochrome c oxidase)
COX3

Other genes:
APP, amyloid β, PINK-1, LRRK2
presenilin-1, α-synuclein, Parkin, DJ-1

hts reserved.

unction

Human_ O-M Mitochondrial Dysfunction

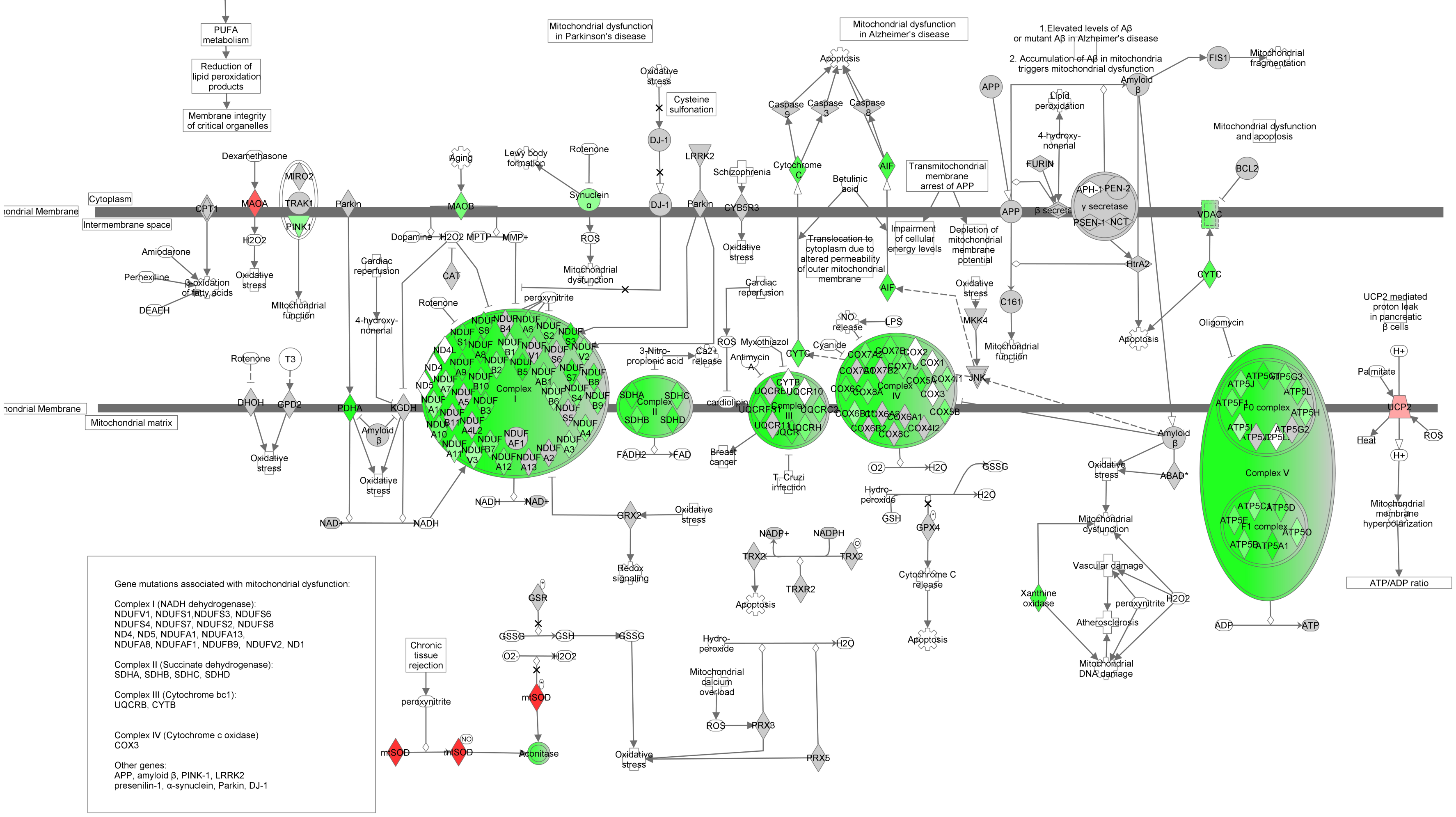

ll rights reserved.
