# Supplementary material for: Conserved and species-specific molecular denominators in mammalian skeletal muscle aging
Source: NPJ Aging Mech Dis. 2017 May 5;3:8. doi: 10.1038/s41514-017-0009-8 (PMC5460213; doi:10.1038/s41514-017-0009-8)
Supplement: Supplementary file 3 — Supplemental Figure 3 [file 41514_2017_9_MOESM3_ESM.pdf]

# Mouse_ O-Y mTOR Signalling Pathway

Path Designer mTOR Signaling

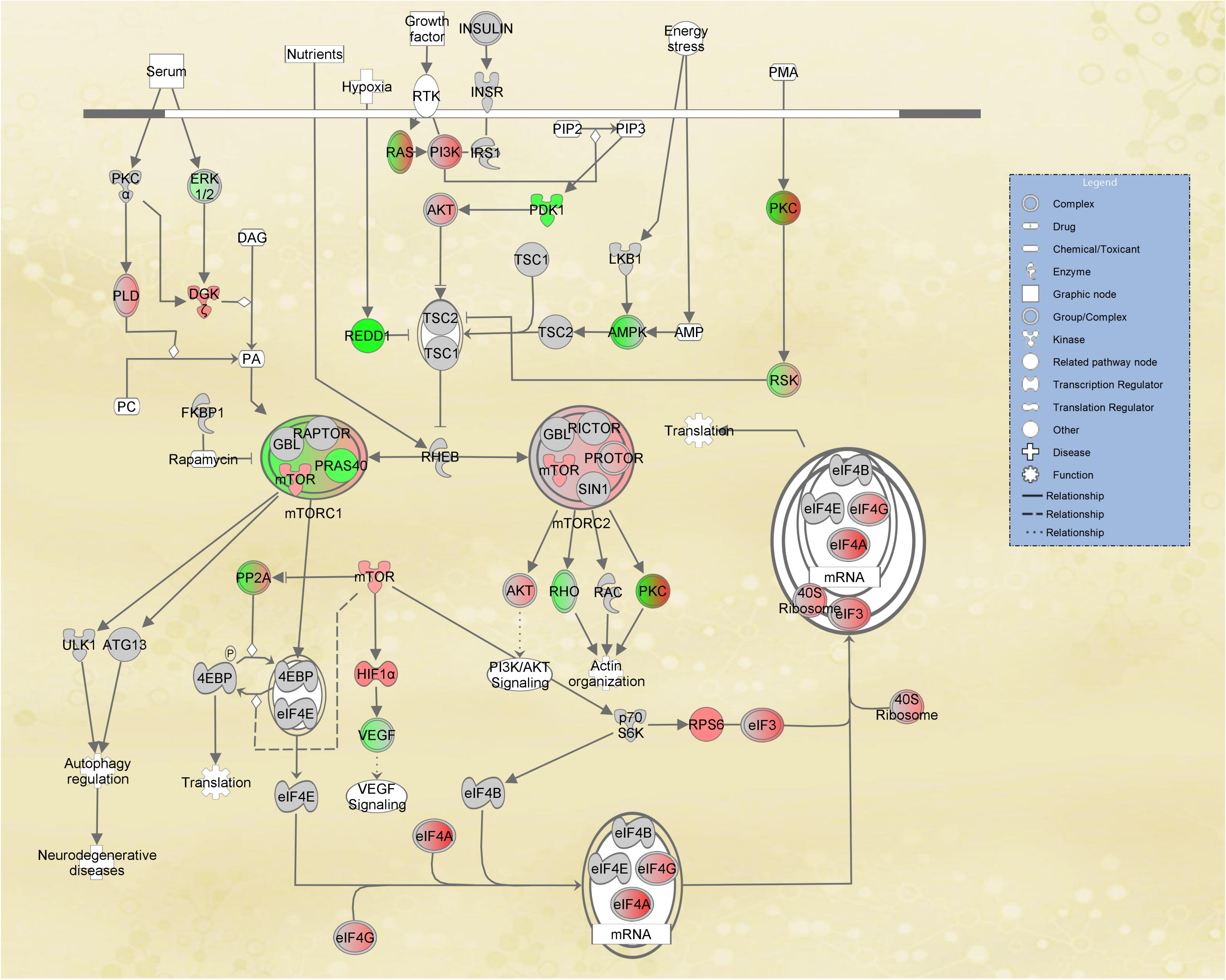


© 2000-2015 QIAGEN. All rights reserved.

# Mouse_ M-Y mTOR Signalling Pathway

Path Designer mTOR Signaling

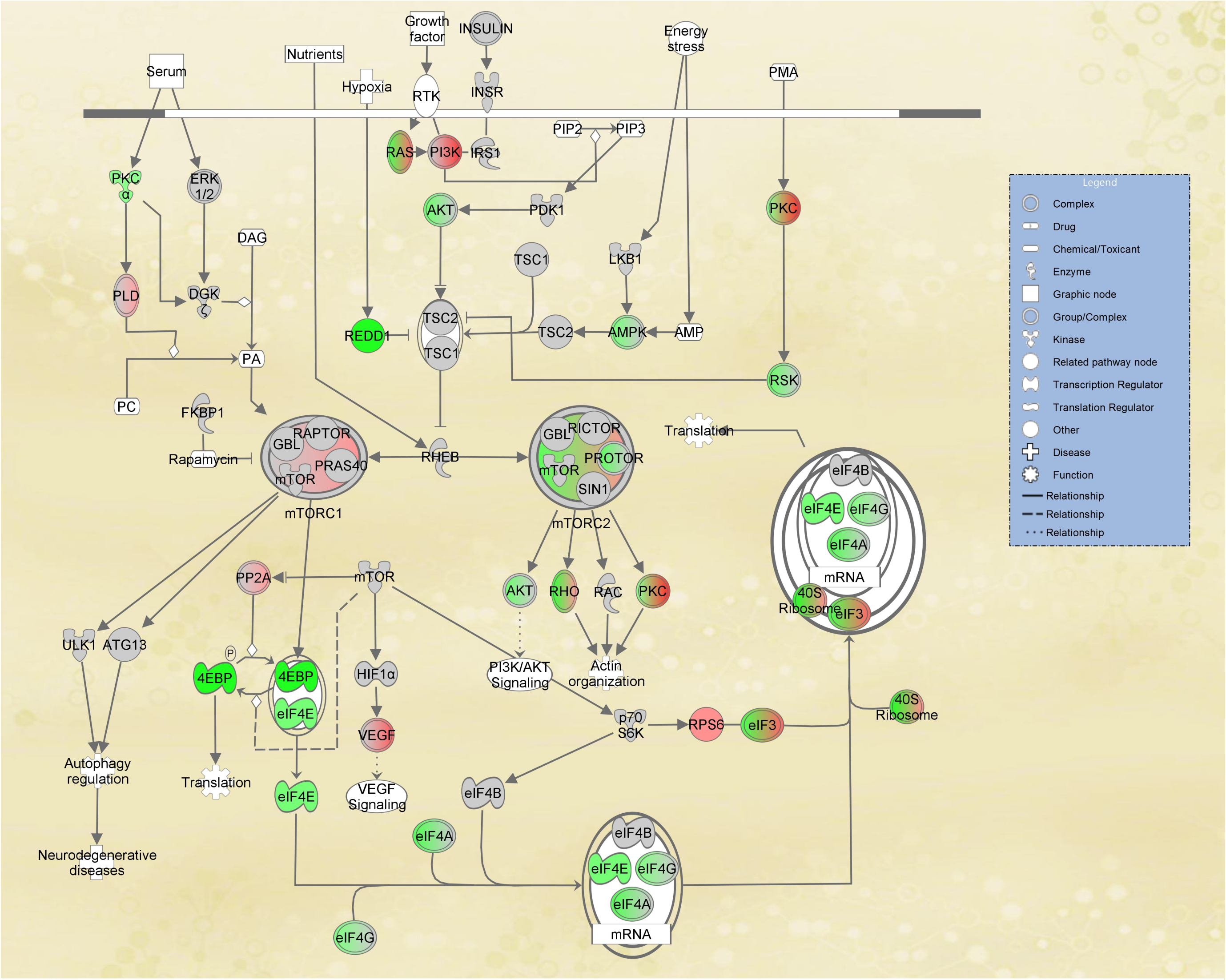


© 2000-2015 QIAGEN. All rights reserved.

# Mouse_ O-M mTOR Signalling Pathway

Path Designer mTOR Signaling

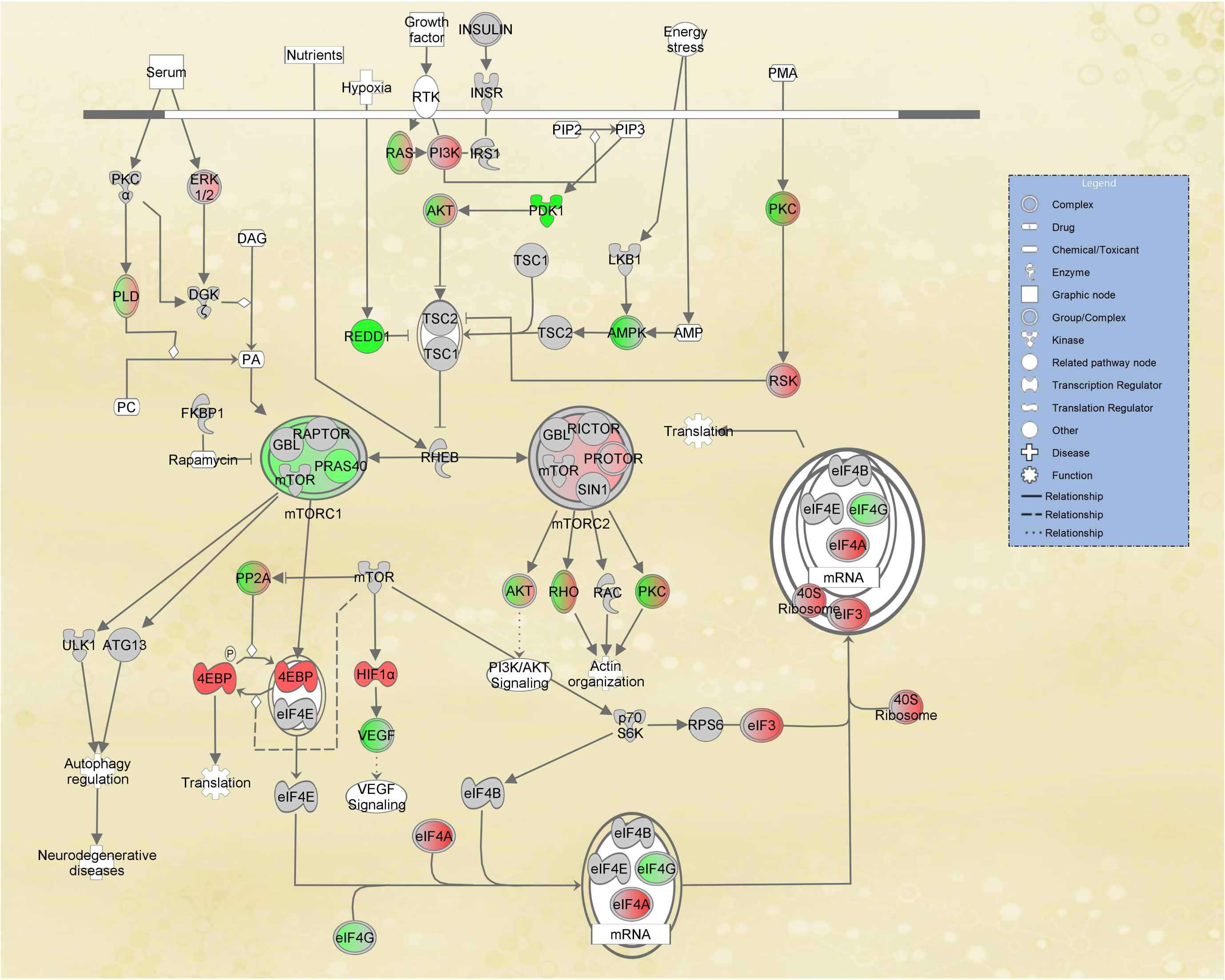


© 2000-2015 QIAGEN. All rights reserved.

# Rat_ O-Y mTOR Signalling Pathway

Path Designer mTOR Signaling

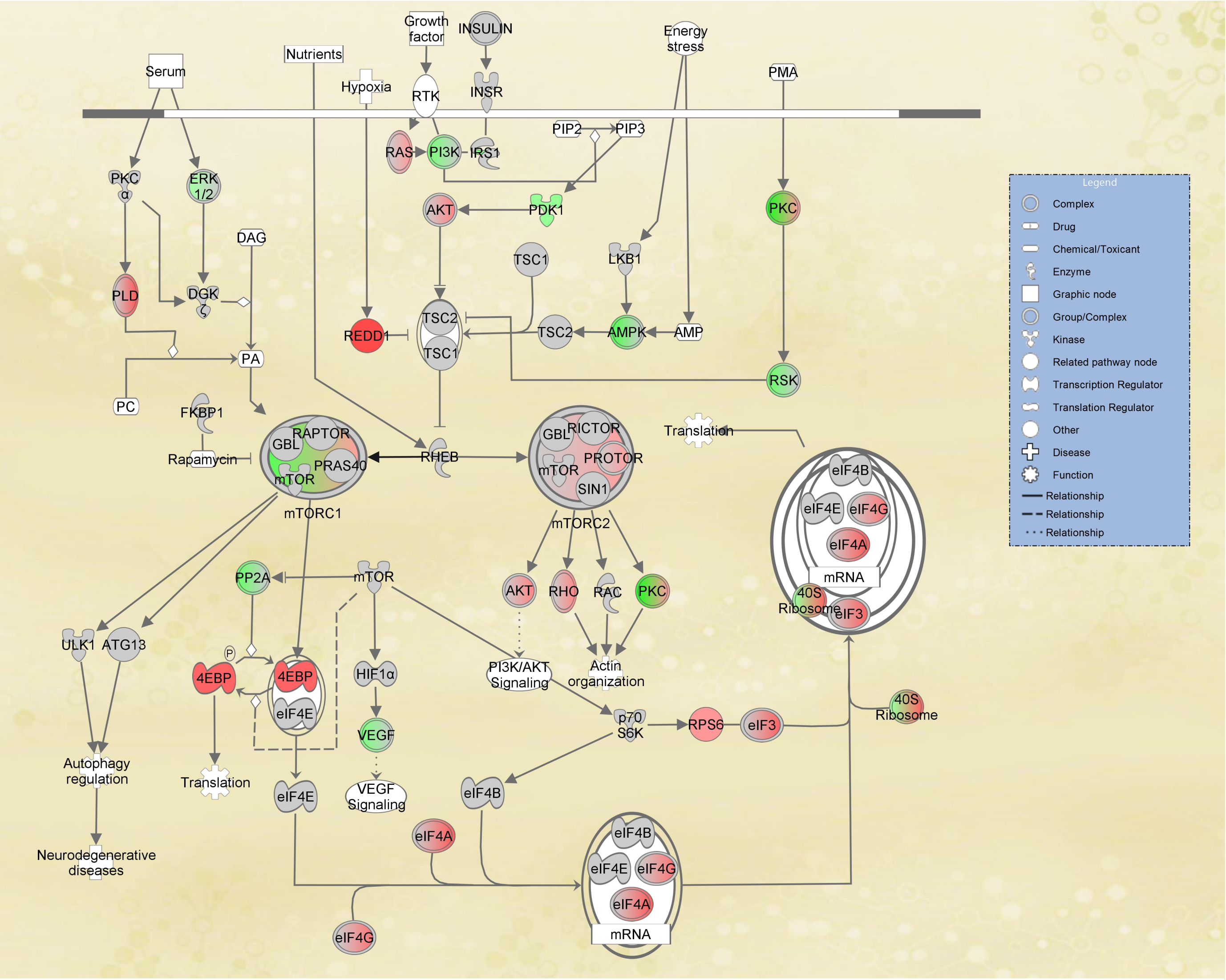


© 2000-2015 QIAGEN. All rights reserved.

Path Designer mTOR Signaling

# Rat_ M-Y mTOR Signalling Pathway

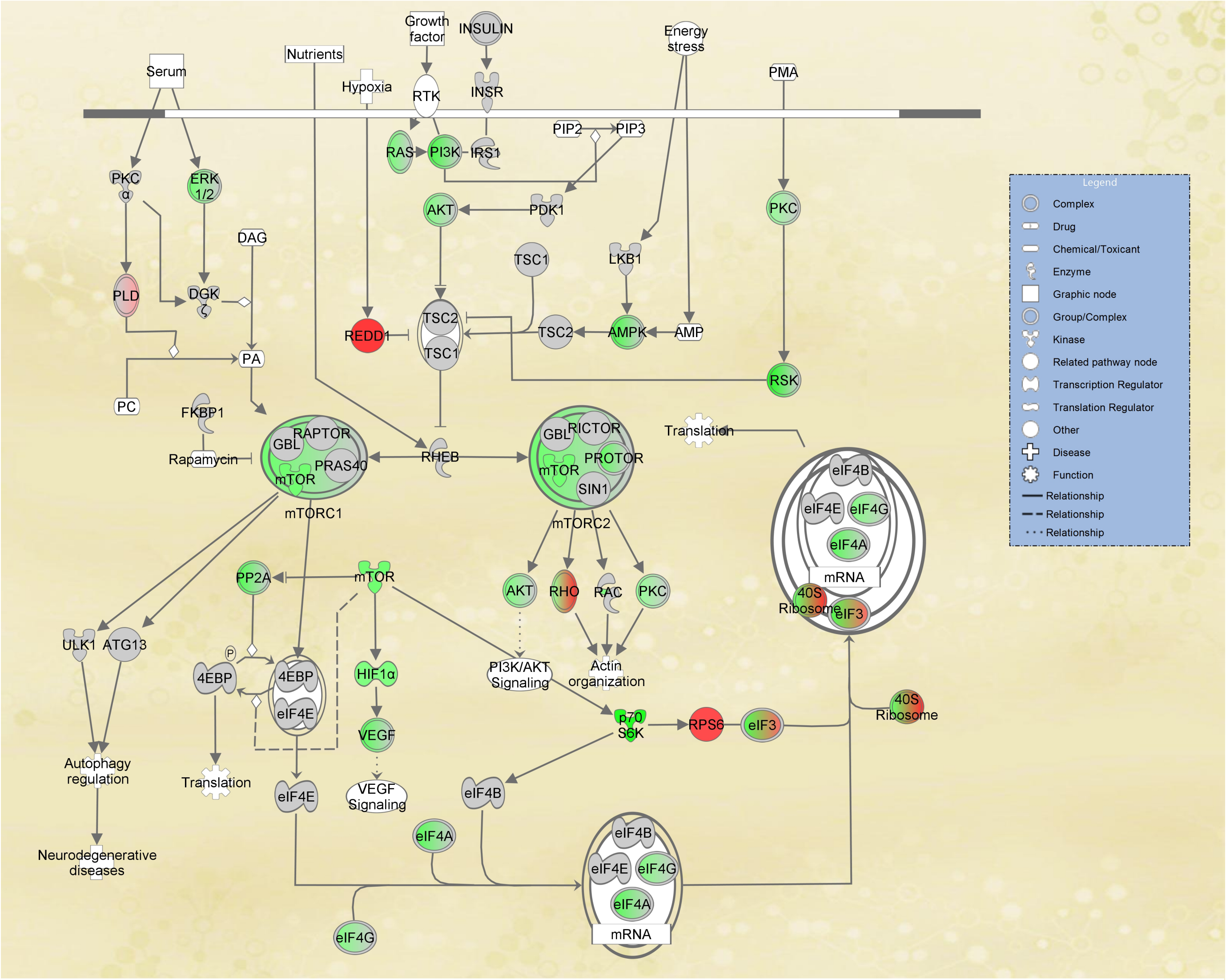


© 2000-2015 QIAGEN. All rights reserved.

# Rat_ O-M mTOR Signalling Pathway

Path Designer mTOR Signaling

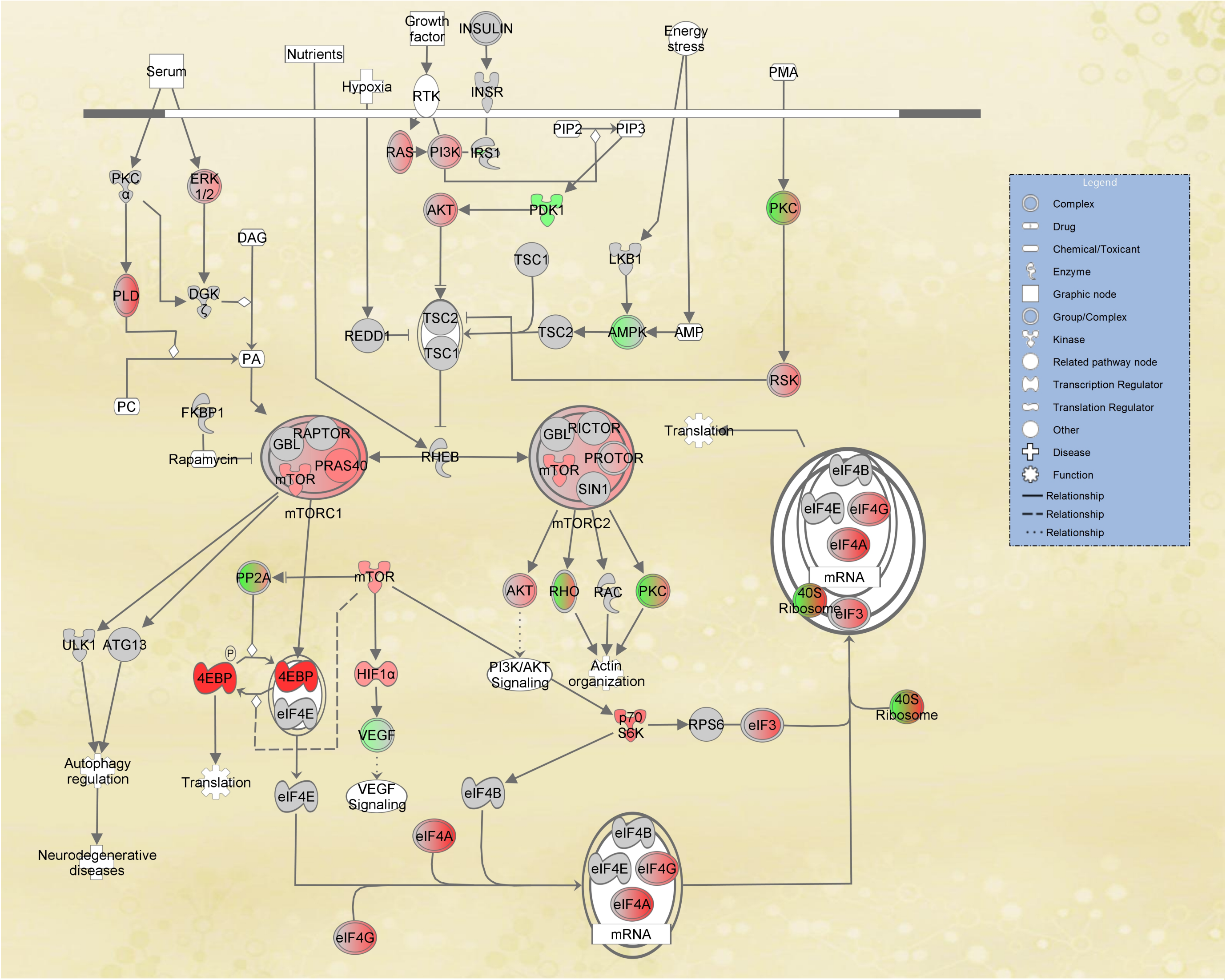


© 2000-2015 QIAGEN. All rights reserved.

# Rhesus_ O-Y mTOR Signalling Pathway

Path Designer mTOR Signaling

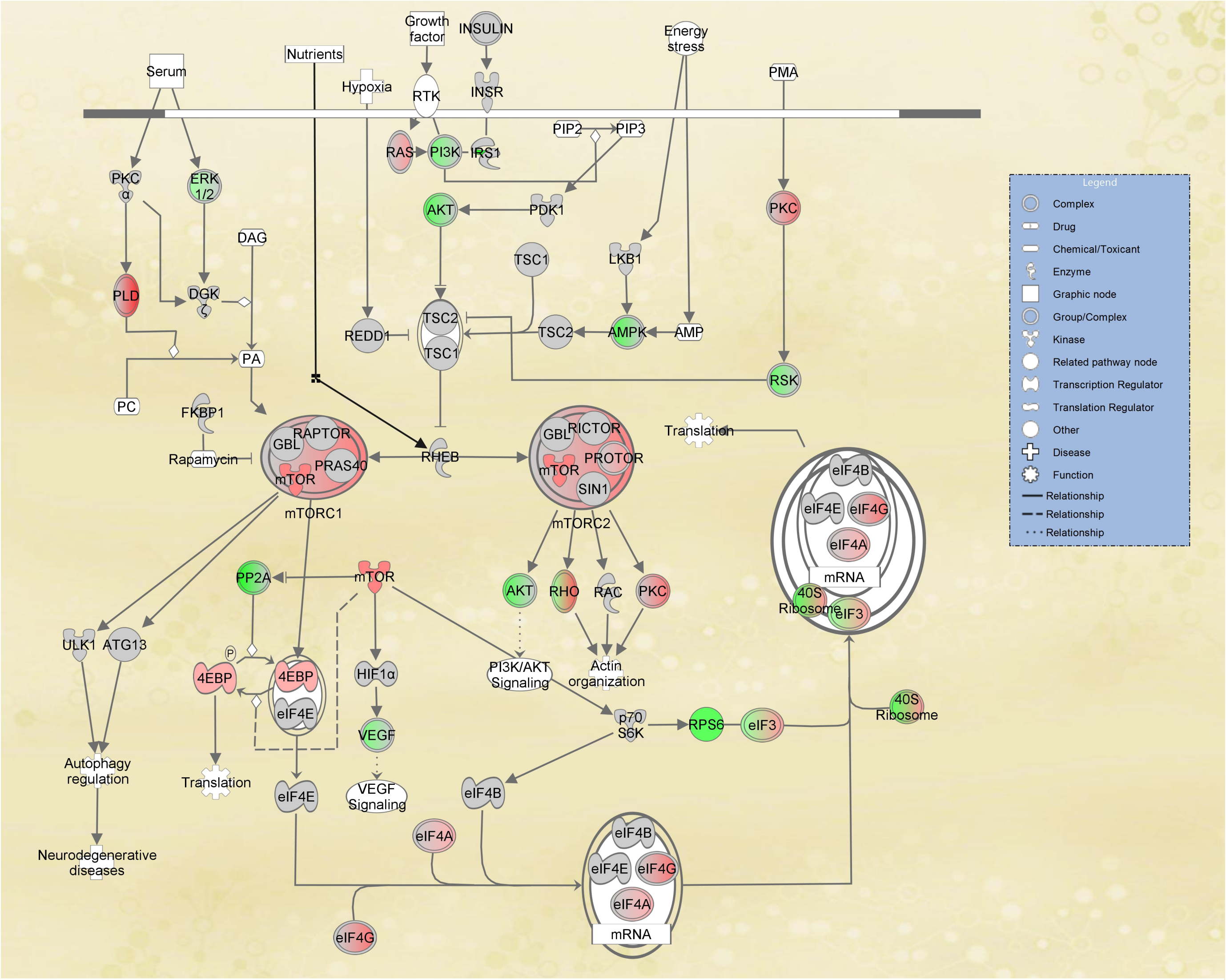


© 2000-2015 QIAGEN. All rights reserved.

# Rhesus_ M-Y mTOR Signalling Pathway

Path Designer mTOR Signaling

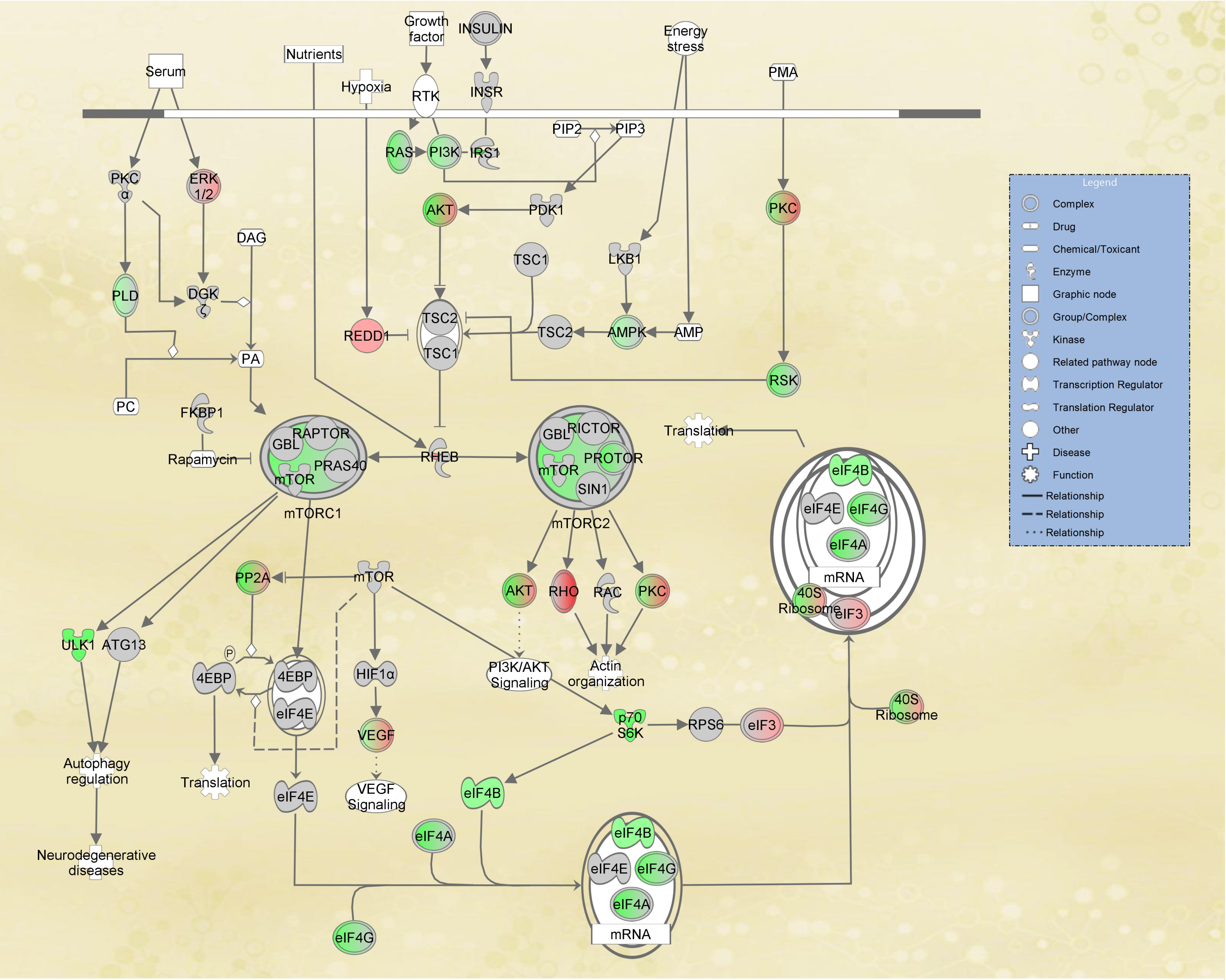


© 2000-2015 QIAGEN. All rights reserved.

# Rhesus_ O-M mTOR Signalling Pathway

Path Designer mTOR Signaling

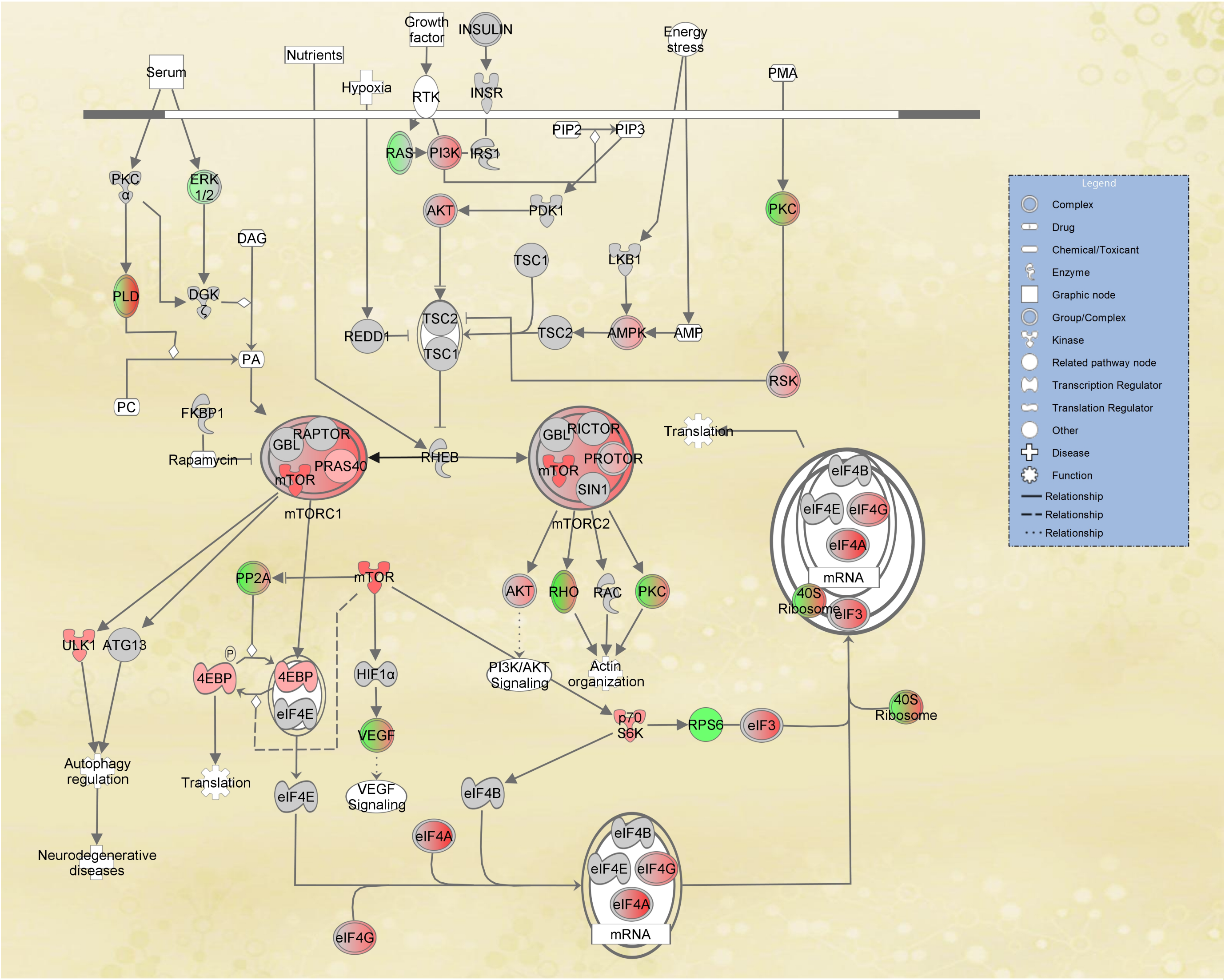


© 2000-2015 QIAGEN. All rights reserved.

# Human_ O-Y mTOR Signalling Pathway

Path Designer mTOR Signaling

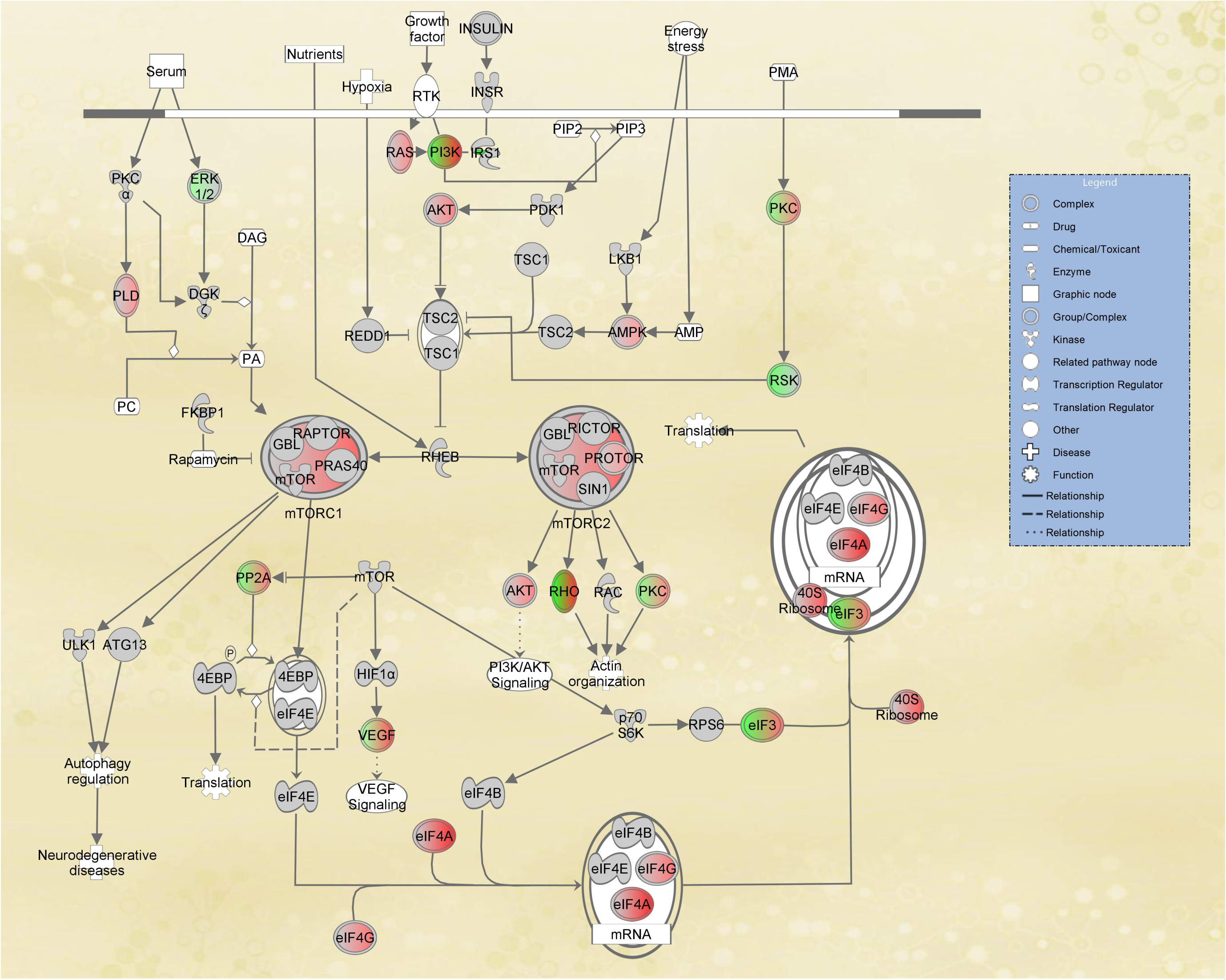


© 2000-2015 QIAGEN. All rights reserved.

# Human_ O-M mTOR Signalling Pathway

Path Designer mTOR Signaling

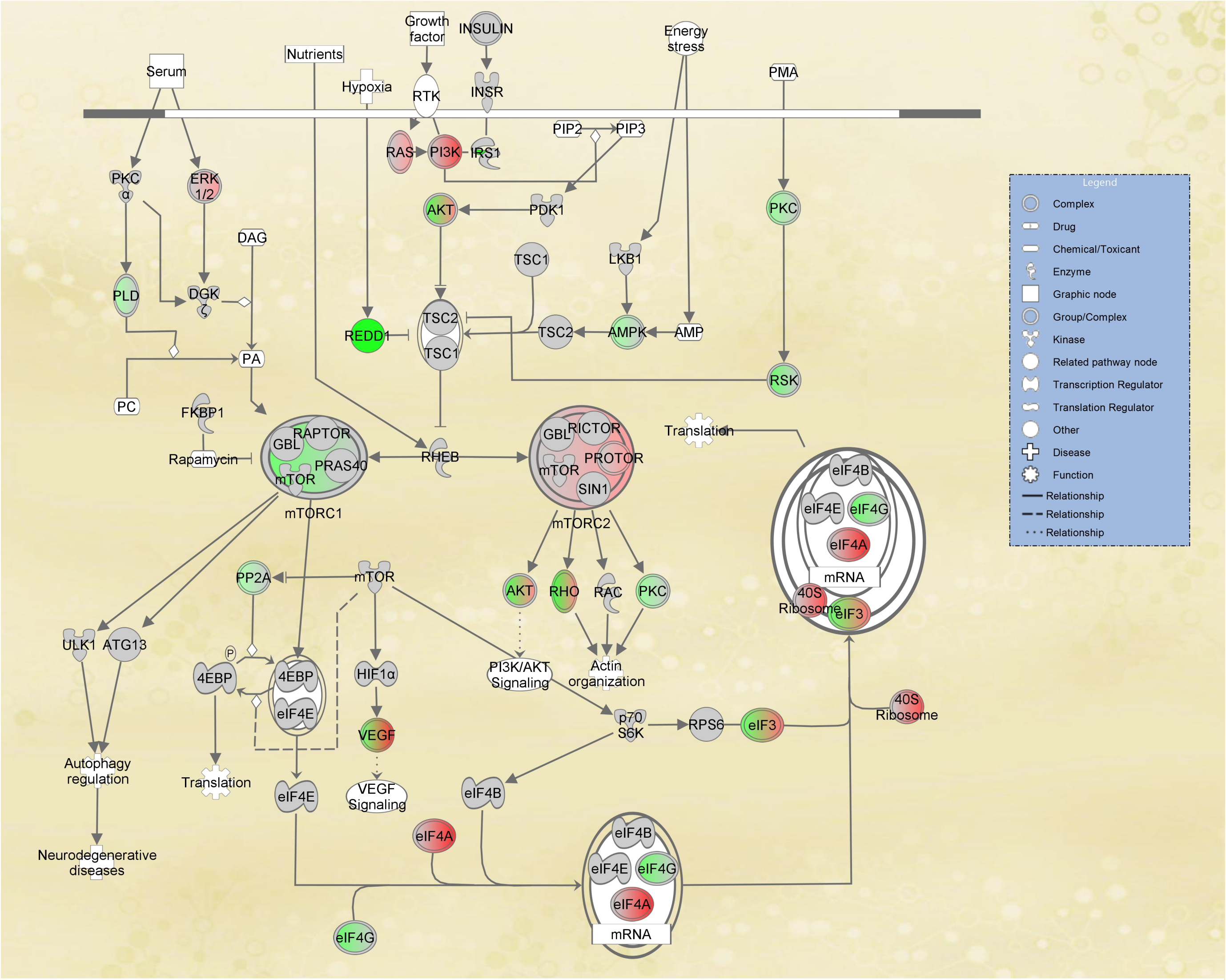


© 2000-2015 QIAGEN. All rights reserved.

# Human_ M-Y mTOR Signalling Pathway

Path Designer mTOR Signaling

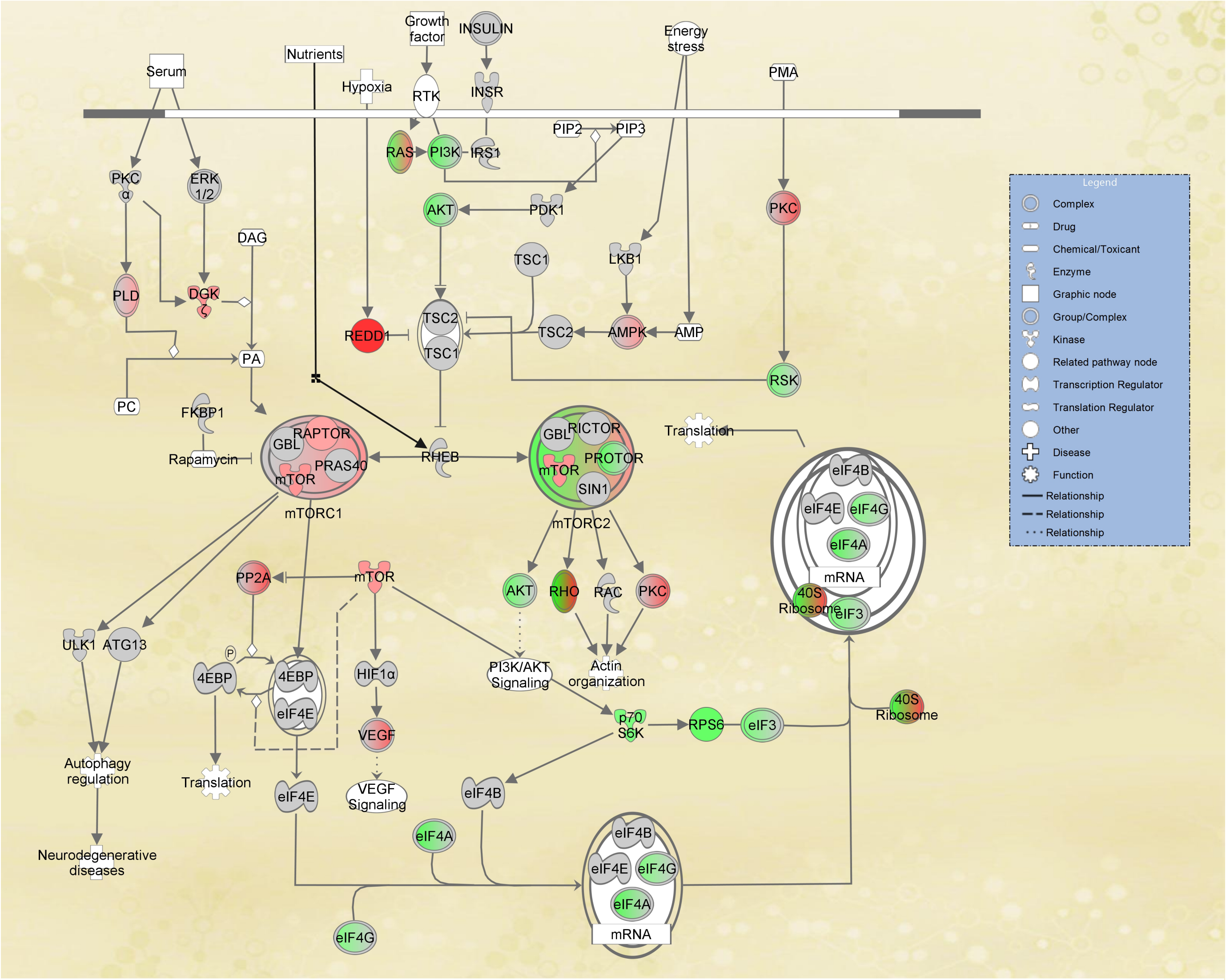


© 2000-2015 QIAGEN. All rights reserved.
