# Supplementary material for: Conserved and species-specific molecular denominators in mammalian skeletal muscle aging
Source: NPJ Aging Mech Dis. 2017 May 5;3:8. doi: 10.1038/s41514-017-0009-8 (PMC5460213; doi:10.1038/s41514-017-0009-8)
Supplement: Supplementary file 4 — Supplementary Figure 4 [file 41514_2017_9_MOESM4_ESM.pdf]

Human_ O-Y IGF-1 Signalling Pathway

Path Designer IGF-1 Signaling for Evi Cross species

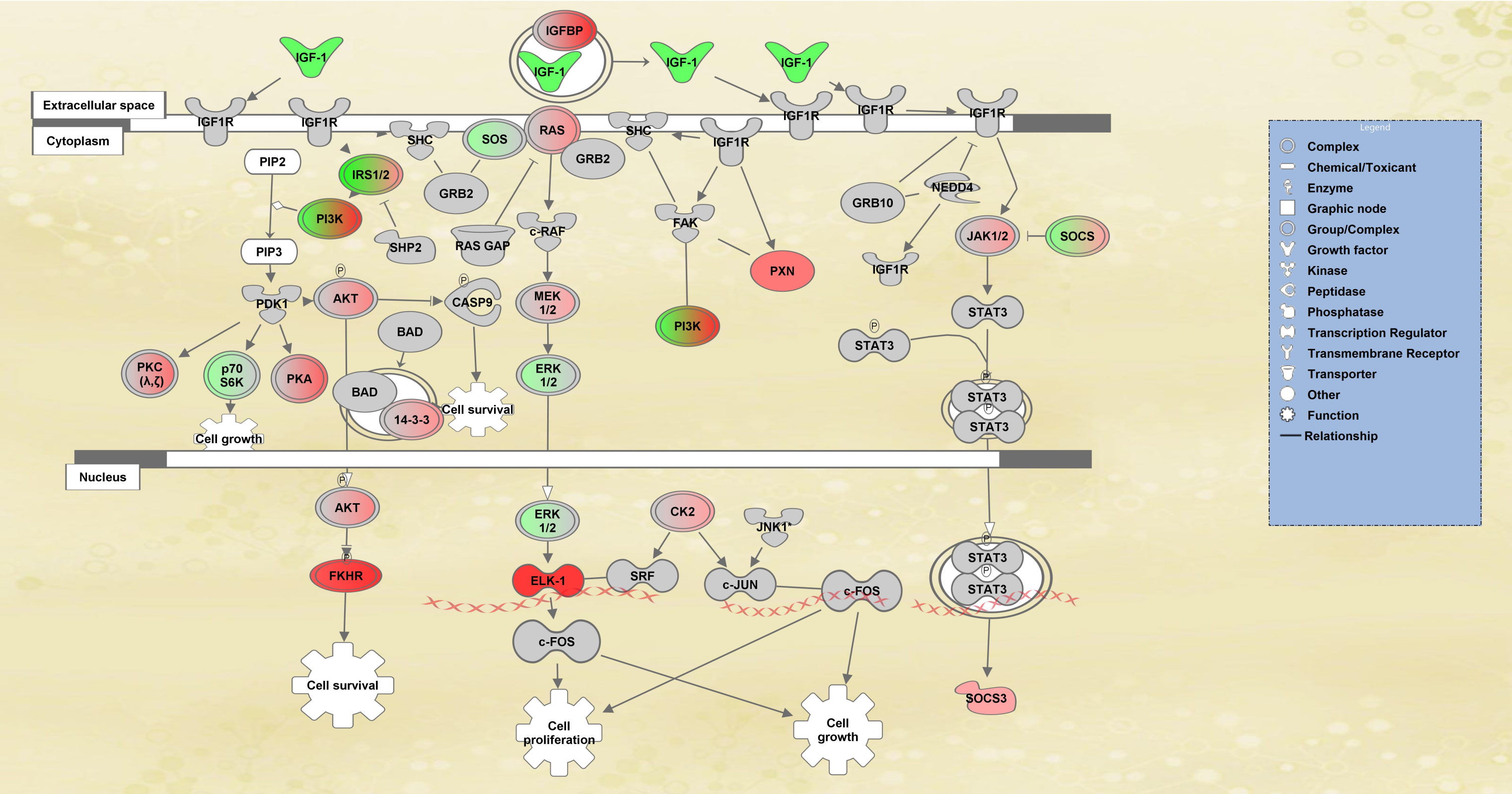

© 2000-2015 QIAGEN. All rights reserved.

Human_ O-M IGF-1 Signalling Pathway

Path Designer IGF-1 Signaling for Evi Cross species

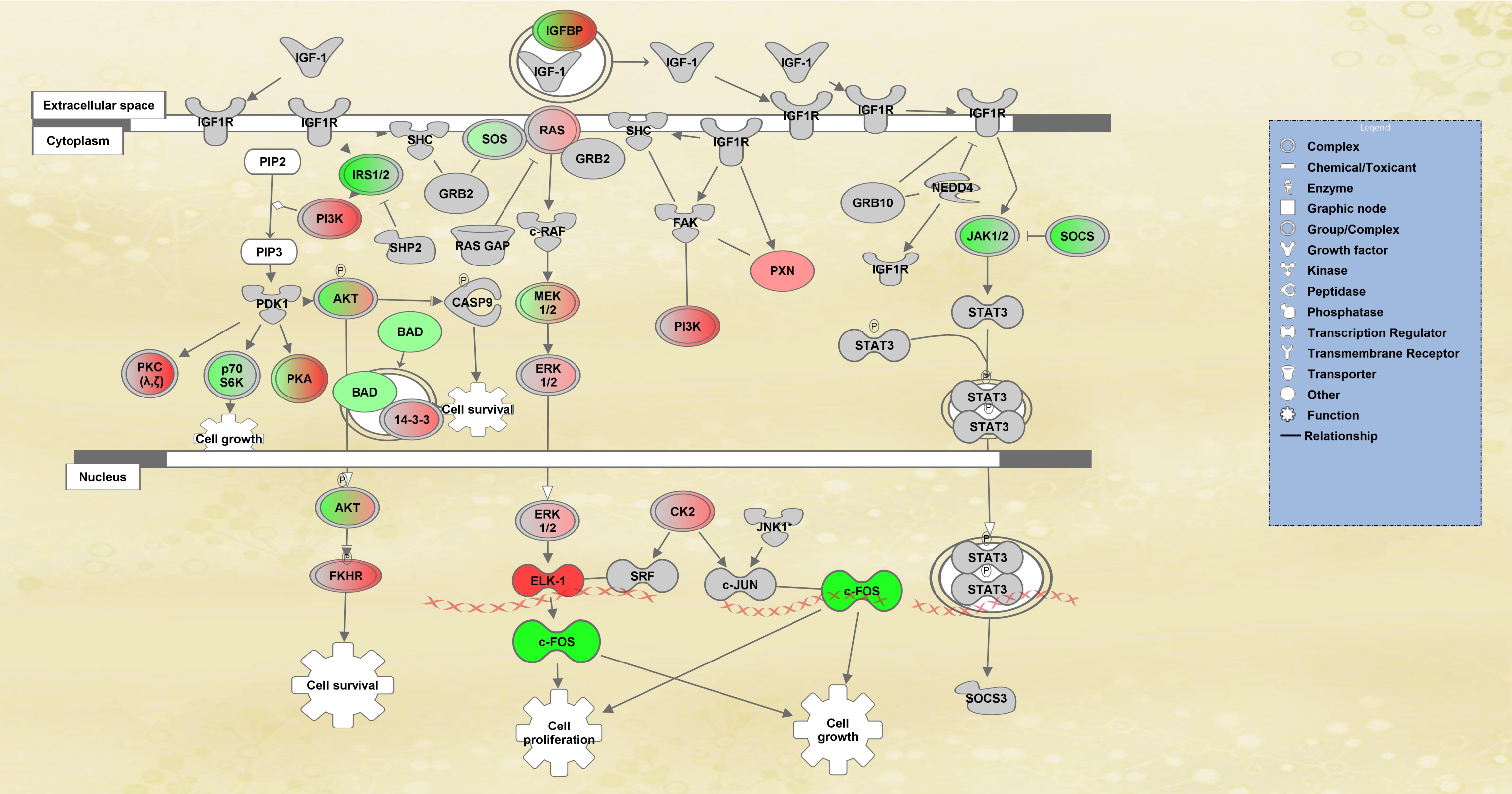

© 2000-2015 QIAGEN. All rights reserved.

Human_ M-Y IGF-1 Signalling Pathway

Path Designer IGF-1 Signaling for Evi Cross species

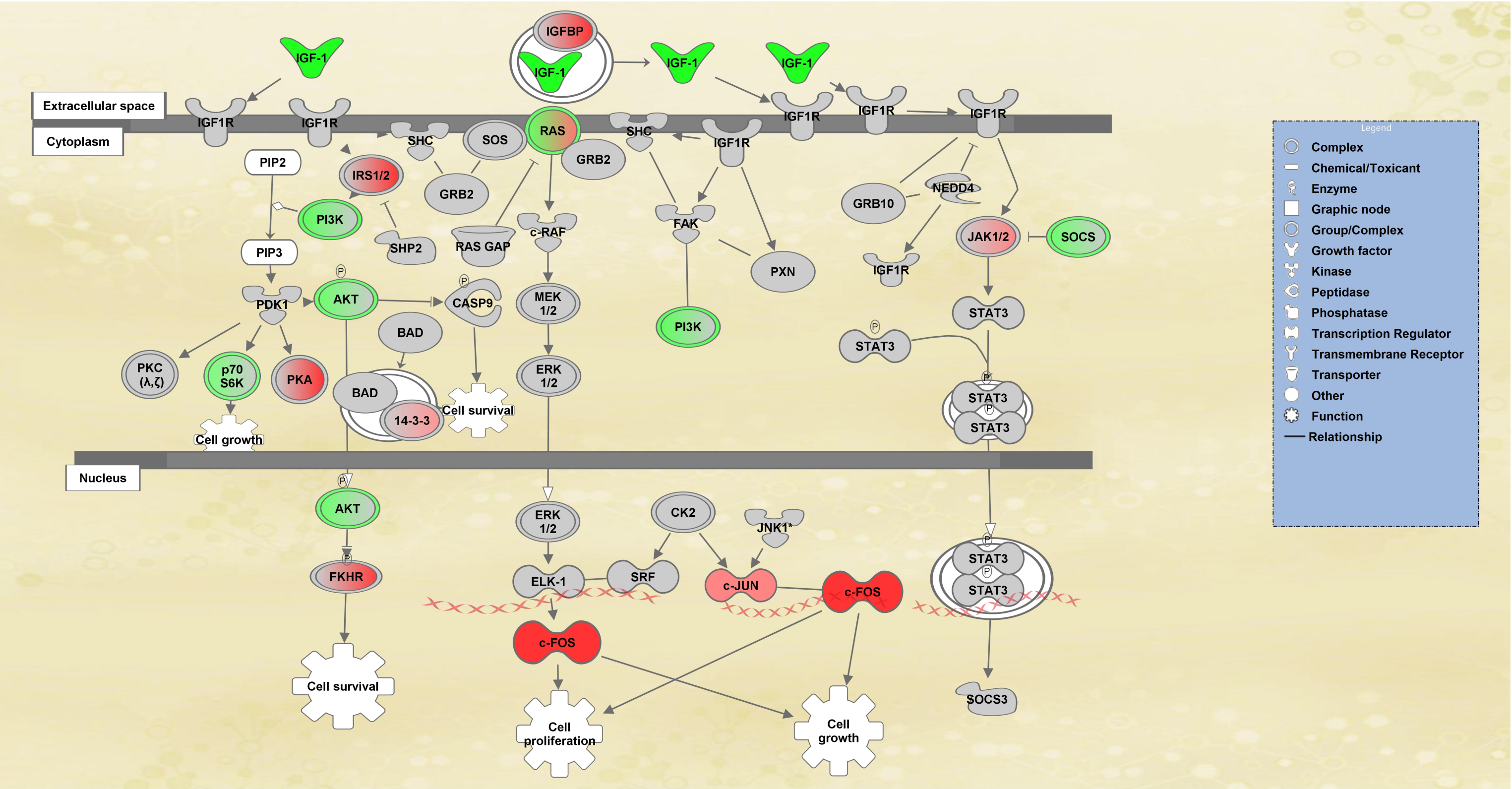

© 2000-2015 QIAGEN. All rights reserved.
