# Supplementary material for: Conserved and species-specific molecular denominators in mammalian skeletal muscle aging
Source: NPJ Aging Mech Dis. 2017 May 5;3:8. doi: 10.1038/s41514-017-0009-8 (PMC5460213; doi:10.1038/s41514-017-0009-8)
Supplement: Supplementary file 9 — Supplemental Table 1 [file 41514_2017_9_MOESM9_ESM.docx]

**Table S1.** Primer sequences used for quantitative PCR analysis

| **MICE** | |  |  |
| --- | --- | --- | --- |
| **Gene** | |  | **Primer sequence** |
| *GAPDH* | | Forward | CACCAACTGCTTAGCCCC |
|  |  | Reverse | TCTTCTGGGTGGCAGTGATG |
| *β-actin* | | Forward | CACTGTCGAGTCGCGTCC |
|  |  | Reverse | TCATCCATGGCGAACTGGTG |
| *ND6* | | Forward | CCCTCCTTCCAACATAACTCC |
|  |  | Reverse | CAGAGACTTGGGGATCTAACTGA |
| *ND1* | | Forward | CTCCTCGTCCCCATTCTAATC |
|  |  | Reverse | GGTTGTTAAAGGGCGTATTGG |
| *ND2* | | Forward | GCATGAGGAGGACTTAACCAA |
|  |  | Reverse | GGGATGGGTTGTAAGGAAGAA |
| *CytB* | | Forward | GCAGTCATAGCCACAGCATTT |
|  |  | Reverse | TCATTCGACTAGGGTTGTTCC |
| *ND5* | | Forward | ACCCATGACTACCATCAGCAA |
|  |  | Reverse | GGAATCGGACCAGTAGGAAAA |
| *IL-10* | | Forward | GCAACTGTTCCTGAACTCAAC |
|  |  | Reverse | ATCTTTTGGGGTCCGTCAACT |
| *TNFα* | | Forward | CCCTCACACTCAGATCATCTTCT |
|  |  | Reverse | GCTACGACGTGGGCTACAG |
| *IL-6* | | Forward | TAGTCCTTCCTACCCCAATTT |
|  |  | Reverse | TTGGTCCTTAGCCACTCCTTC |
| *IL-1β* | | Forward | GCAACTGTTCCTGAACTCAAC |
|  |  | Reverse | ATCTTTTGGGGTCCGTCAACT |
| *NDUFAB1* | | Forward | GGACCGAGTTCTGTATGTCTTG |
|  |  | Reverse | AAACCCAAATTCGTCTTCCATG |
| *UQCRCR1* | | Forward | ATCAAGGCACTGTCCAAGG |
|  |  | Reverse | TCATTTTCCTGCATCTCCCG |
| *UQCRC2* | | Forward | GTCAGAGGGCTTCCTGAGTG |
|  |  | Reverse | ACTCGTCGAGAAAAGGCGTA |
| *ATP5a1* | | Forward | GCCCTCGGTAATGCTATTGA |
|  |  | Reverse | GCAATCGATGTTTTCCCAGT |
| *SDHb* | | Forward | ACCCCTTCTCTGTCTACCG |
|  |  | Reverse | AATGCTCGCTTCTCCTTGTAG |
| *SDHd* | | Forward | CTTGAATCCCTGCTCTGTGG |
|  |  | Reverse | AAAGCTGAGAGTGCCAAGAG |
| *ND3* | | Forward | AAGCAAATCCATATGAATGCGG |
|  |  | Reverse | GCTCATGGTAGTGGAAGTAGAAG |
| *ND4I* | | Forward | CCAACTCCATAAGCTCCATACC |
|  |  | Reverse | GATTTTGGACGTAATCTGTTCCG |
| *COX1* | | Forward | CCCAGATATAGCATTCCCACG |
|  |  | Reverse | ACTGTTCATCCTGTTCCTGC |
| *COX2* | | Forward | AGTTGATAACCGAGTCGTTCTG |
|  |  | Reverse | CTGTTGCTTGATTTAGTCGGC |
| *COX3* | | Forward | CGTGAAGGAACCTACCAAGG |
|  |  | Reverse | CGCTCAGAAGAATCCTGCAA |
| *ATP6* | | Forward | TCCCAATCGTTGTAGCCATC |
|  |  | Reverse | TGTTGGAAAGAATGGAGTCGG |
| *ATP8* | | Forward | GCCACAACTAGATACATCAACATG |
|  |  | Reverse | TGGTTGTTAGTGATTTTGGTGAAG |
| **Primers used for mtDNA analysis** | | | |
| *COX2* | Forward | | ATAACCGAGTCGTTCTGCCAAT |
|  | Reverse | | TTTCAGAGCATTGGCCATAGAA |
| *Rsp18* | Forward | | TGTGTTAGGGGACTGGTGGACA |
|  | Reverse | | CATCACCCACTTACCCCCAAAA |

| **Rhesus monkeys** |  |  |
| --- | --- | --- |
| **Gene** |  | **Primer sequence** |
| *GAPDH* | Forward | GAGTCAACGGATTTGGTCGT |
|  | Reverse | GGGTGGAATCATACTGGAACA |
| *β-actin* | Forward | GCGCGGCTACAGCTTCA |
|  | Reverse | CTTAATGTCACGCACGATTTCC |
| *ND6* | Forward | ACCACTTCTACCGCCTCATC |
|  | Reverse | AGTATCCTGAGACATGGGGTTC |
| *ND1* | Forward | TCCATGCGGACTATTACAACC |
|  | Reverse | ATAGGAGGAGGGCAATGAAGA |
| *ND2* | Forward | CAGAAGTCACCCAAGGAACAC |
|  | Reverse | TGGAGAGGGTTAGGAGGATGT |
| *CytB* | Forward | GTCCAATGAATCTGAGGAGGA |
|  | Reverse | TGGTTGTGTAGTAGGGGTGGA |
| *ND5* | Forward | CCTGAGCCCTATTCATCACTCT |
|  | Reverse | GGTTTAGTAGAGCGGGGTTGT |
| *IL-10* | Forward | TTTTACCTGGAGGAGGTGATG |
|  | Reverse | GCCTTGCTCTTGTTTTCACAG |
| *TNFα* | Forward | CTCTTCTCATTCCTGCTCGTG |
|  | Reverse | GCTTGGTGGTTTGCTACGAC |
| *IL-6* | Forward | AGCCAGAGTCATTCAGAGCAA |
|  | Reverse | ATTGGAAGTTGGGGTAGGAAG |
| *IL-1β* | Forward | AAAAATGCCTCGTGCTGTCT |
|  | Reverse | TCGTTGCTTGTCTCTCCTTGT |
| *NDUFAB1* | Forward | CGCAGGTTCCTGGTAGAGTTA |
|  | Reverse | ACAGTCCTGGATGCCCTCTAA |
| *UQCRC2* | Forward | ACTTGCATCCAGTCTGACGAC |
|  | Reverse | CCTTGTTGCGGTCACACTTA |
| *ATP5a1* | Forward | GCAGATGCCATGAAGTACACC |
|  | Reverse | GCAGATGCCATGAAGTACACC |
| *SDHb* | Forward | CCAGAGACGACTTCACAGAGG |
|  | Reverse | CCTGGATTCAGACCCTTAGGA |
| *ND3* | Forward | TTCTGACTACCCCAGCTCAAC |
|  | Reverse | GATGCGAGCAGGGTTTAGG |
| *ND4I* | Forward | CCCCTATATGGCCTACACCTG |
|  | Reverse | GTACTGCGGCAAGGACTATTG |
| *COX1* | Forward | CCCCCTTCTTTCCTGCTACTA |
|  | Reverse | GGTGGGAGAAGTTTCCTGCTA |
| *COX2* | Forward | CCCCACACTAGGCCTAAAAAC |
|  | Reverse | GTCCGTAATAGACGCCTGGTC |
| *COX3* | Forward | GTCGGCTTCTTCTGAGCATT |
|  | Reverse | CTATGGGGTTTAGAGGGGTGA |
| *ATP6* | Forward | GCTAAGGGACAAACCTGATCC |
|  | Reverse | GGTAGTTGGCGTGAACGAGTA |
| *ATP8* | Forward | TGACGATGCTTCCCACACTA |
|  | Reverse | GTCAGCGGACATTGTGAGACT |
| **Primers used for mtDNA analysis** | | |
| NADH1 | Forward | CCCTAAAACCCGCCACATCT |
|  | Reverse | CGATGGTGAGAGCTAAGGTC |
| β-actin | Forward | GCGCGGCTACAGCTTCA |
|  | Reverse | CTTAATGTCACGCACGATTTCC |

| **Humans** |  |  |
| --- | --- | --- |
| **Gene** |  | **Primer sequence** |
| *GAPDH* | Forward | CAATGACCCCTTCATTGACC |
|  | Reverse | GACAAGCTTCCCGTTCTCAG |
| *β-actin* | Forward | CTGGCTCCTAGCACCATGAAGAT |
|  | Reverse | GGTGGACAGTGAGGCCAGGAT |
| *ND6* | Forward | GGTGCTGTGGGTGAAAGAGTA |
|  | Reverse | CCTGACCCCTCTCCTTCATAA |
| *ND1* | Forward | TACTACAACCCTTCGCTGACG |
|  | Reverse | AGCGATGGTGAGAGCTAAGGT |
| *ND2* | Forward | CCGGACAATGAACCATAACC |
|  | Reverse | CTCAGAAGTGAAAGGGGGCTA |
| *CytB* | Forward | CTAGCCAACCCCTTAAACACC |
|  | Reverse | CAAGGACGCCTCCTAGTTTGT |
| *ND5* | Forward | TCTCAGCCCTACTCCACTCAA |
|  | Reverse | GTGGGCTATTTTCTGCTAGGG |
| *IL-10* | Forward | GAACCAAGACCCAGACATCAA |
|  | Reverse | AAAGGCATTCTTCACCTGCTC |
| *TNFα* | Forward | TAGCCCATGTTGTAGCAAACC |
|  | Reverse | GCTGGTTATCTCTCAGCTCCA |
| *IL-6* | Forward | ATGAGGAGACTTGCCTGGTG |
|  | Reverse | GGAACTGGATCAGGACTTTTGT |
| *IL-1β* | Forward | TGGGATAACGAGGCTTATGTG |
|  | Reverse | ATGGAGAACACCACTTGTTGC |
| *NDUFAB1* | Forward | CGCAGGTTCCTGGTAGAGTTA |
|  | Reverse | AAAGAACACGGTCCTGGATG |
| *UQCRCR1* | Forward | GCATCTATGCGAGATGTGGTC |
|  | Reverse | GCTTCCTGACATTCTCACTGG |
| *UQCRC2* | Forward | TAAGTGTGACCGCAACAAGG |
|  | Reverse | GGTGACATTGAGCAGGAACTC |
| *ATP5a1* | Forward | CTGACGGACAGATCTTCTTGG |
|  | Reverse | GGATCCGACACGAGATACAGA |
| *SDHb* | Forward | GCAGTCCATAGAAGAGCGTGA |
|  | Reverse | TTGTCTCCGTTCCACCAGTAG |
| *SDHd* | Forward | CGATCTCAGCTTACTGCAACC |
|  | Reverse | CCCTGTAATGCCAGCTACTTG |
| *ND3* | Forward | CCTTTTACCCCTACCATGAGC |
|  | Reverse | GCCAGACTTAGGGCTAGGATG |
| *ND4* | Forward | ACTCATTCTCAACCCCCTGAC |
|  | Reverse | GTTTGTCGTAGGCAGATGGAG |
| *COX1* | Forward | TACGTTGTAGCCCACTTCCAC |
|  | Reverse | GGGTGTAGCCTGAGAATAGGG |
| *COX2* | Forward | GCAGCGCAAGTAGGTCTACAA |
|  | Reverse | GGGCATACAGGACTAGGAAGC |
| *COX3* | Forward | GGCATCTACGGCTCAACATT |
|  | Reverse | GCGGATGAAGCAGATAGTGAG |
| *ATP6* | Forward | CCTACACCCCTTATCCCCATA |
|  | Reverse | GTGGCCTGCAGTAATGTTAGC |
| *ATP8* | Forward | ATACTACCGTATGGCCCACCA |
|  | Reverse | CTTTGGTGAGGGAGGTAGGTG |
| **Primers used for mtDNA** | | |
| ND1 | Forward | CCCTAAAACCCGCCACATCT |
|  | Reverse | GAGCGATGGTGAGAGCTAAGGT |
| β-actin | Forward | ACCCACACTGTGCCCATCTAC |
|  | Reverse | TCGGTGAGGATCTTCATGAGGTA |
