# Supplementary material for: Conserved and species-specific molecular denominators in mammalian skeletal muscle aging
Source: NPJ Aging Mech Dis. 2017 May 5;3:8. doi: 10.1038/s41514-017-0009-8 (PMC5460213; doi:10.1038/s41514-017-0009-8)
Supplement: Supplementary file 10 — Supplemental Table 2 [file 41514_2017_9_MOESM10_ESM.docx]

**Table S2:** Up- and down-regulated pathway interactions between the different species and ages

|  | **Pathways** | **M** | **R** | **RH** | **H** | **TOTAL** |
| --- | --- | --- | --- | --- | --- | --- |
| **M-Y** | **Up** | 41 | 147 | 26 | 216 | 430 |
|  | **Down** | 26 | 69 | 68 | 17 | 180 |
| **O-M** | **Up** | 68 | 74 | 74 | 158 | 374 |
|  | **Down** | 57 | 97 | 29 | 95 | 278 |
| **O-Y** | **Up** | 80 | 102 | 44 | 231 | 457 |
|  | **Down** | 58 | 79 | 35 | 61 | 233 |

M: mouse, R: rat, RH: rhesus monkey, H: human, Y: young, M: middle-aged, O: old.
