# Supplementary material for: Conserved and species-specific molecular denominators in mammalian skeletal muscle aging
Source: NPJ Aging Mech Dis. 2017 May 5;3:8. doi: 10.1038/s41514-017-0009-8 (PMC5460213; doi:10.1038/s41514-017-0009-8)
Supplement: Supplementary file 12 — Supplemental Table 4 [file 41514_2017_9_MOESM12_ESM.docx]

**Table S4**. Z-ratio of the top 20 up- and down-regulated inflammatory genes between the different species and ages.

| **M-Y** |  |  |  |  |  |  |  |
| --- | --- | --- | --- | --- | --- | --- | --- |
| **Mice** |  | **Rats** |  | **Rhesus** |  | **Humans** |  |
| **Symbol** | **Zratio** | **Symbol** | **Zratio** | **Symbol** | **Zratio** | **Symbol** | **Zratio** |
| LEP | 18.76 | THBS4 | 11.02 | FOS | 16.48 | CEBPD | 9.49 |
| ADIPOQ | 10.43 | SLPI | 4.29 | EGR1 | 11.99 | ZFP36 | 7.41 |
| CDO1 | 5.24 | CEBPB | 4.23 | ZFP36 | 7.81 | ZP3 | 7.12 |
| ORM1 | 4.58 | KERA | 3.91 | TFF2 | 7.54 | CEBPB | 5.13 |
| CCL11 | 3.74 | FABP4 | 3.68 | PLA2G2A | 6.80 | ACOT11 | 5.11 |
| LUM | 3.60 | CD36 | 3.57 | TNFRSF12A | 6.66 | THBS4 | 4.99 |
| HP | 3.51 | HSPB1 | 3.11 | APOE | 6.29 | FOS | 4.79 |
| TNFRSF12A | 3.51 | PDE4B | 3.11 | ART3 | 5.70 | HDAC4 | 4.63 |
| AOC3 | 3.39 | GPX4 | 2.90 | PLA2G10 | 5.39 | SLPI | 4.60 |
| CD276 | 3.38 | IER3 | 2.82 | HP | 4.81 | ANGPT1 | 4.19 |
| IL33 | 3.23 | PARK7 | 2.79 | AOX1 | 4.47 | MCL1 | 3.85 |
| PIK3CB | 3.05 | CCL11 | 2.78 | PROS1 | 4.46 | CCL21 | 3.71 |
| APP | 3.01 | CFH | 2.78 | PTGIS | 3.67 | GSN | 3.41 |
| MAOB | 2.84 | GSN | 2.58 | LYZ | 3.51 | LYZ | 3.33 |
| SERPING1 | 2.67 | ZFP36 | 2.57 | CAV1 | 3.50 | IER3 | 3.32 |
| PRKG1 | 2.65 | SLC37A4 | 2.34 | DARC | 3.38 | SFRP1 | 3.19 |
| EPHX2 | 2.60 | CXCL12 | 2.32 | EDN1 | 3.26 | FOXO3 | 3.19 |
| NFE2L1 | 2.56 | CDC42 | 2.32 | HSPB1 | 3.14 | PRNP | 3.19 |
| APOE | 2.54 | LTBP1 | 2.20 | CSF2RA | 3.04 | KERA | 3.09 |
| CASR | 2.50 | CTSB | 2.20 | SLC7A2 | 3.00 | S100A4 | 3.07 |
| ADRB2 | -6.79 | TOLLIP | -2.22 | MAPK9 | -2.51 | MIF | -2.42 |
| JAK2 | -5.75 | NFATC3 | -2.23 | TNFSF12 | -2.56 | DARC | -2.46 |
| SOCS3 | -4.55 | CCR7 | -2.34 | EPHX2 | -2.81 | JAM3 | -2.47 |
| NR4A2 | -3.87 | PYCARD | -2.47 | AKT1 | -3.00 | PLG | -2.55 |
| SLC37A4 | -3.53 | CCRL2 | -2.67 | MGLL | -3.37 | PDE4B | -2.64 |
| PTX3 | -3.29 | CCND1 | -2.68 | IER3 | -3.39 | F2R | -2.65 |
| PON1 | -3.21 | PIK3CD | -2.79 | THBS1 | -3.64 | PTN | -2.65 |
| CXCL1 | -3.13 | JAK2 | -2.81 | CXCR7 | -3.78 | HP | -2.84 |
| SMAD3 | -2.87 | PLAU | -2.99 | CIRBP | -3.90 | MMP9 | -2.91 |
| IGF1 | -2.66 | CNTF | -3.02 | NCL | -4.03 | SELP | -2.98 |
| CEBPB | -2.59 | FYN | -3.06 | PELI1 | -4.16 | LGALS1 | -3.05 |
| EGR1 | -2.54 | AGT | -3.11 | PDE4B | -4.18 | PPBP | -3.07 |
| EDNRB | -2.50 | MCL1 | -3.15 | SLC37A4 | -4.18 | LUM | -3.10 |
| PLA2G7 | -2.49 | ELN | -3.56 | ANGPTL2 | -4.31 | CCL5 | -3.14 |
| GNAI2 | -2.41 | MMP14 | -3.65 | CD74 | -4.50 | TNC | -3.35 |
| TRAF3IP2 | -2.33 | MAPK9 | -3.70 | KERA | -4.69 | IGF1 | -3.37 |
| B4GALT1 | -2.30 | MTA1 | -3.71 | PARP1 | -4.85 | CCL14 | -3.63 |
| ELN | -2.18 | PROS1 | -3.77 | CX3CR1 | -6.22 | GJA1 | -5.50 |
| NFKB1 | -2.15 | FN1 | -4.42 | THBS4 | -6.37 | LITAF | -5.65 |
| GADD45A | -2.07 | NUPR1 | -4.44 | GADD45A | -6.55 | SPP1 | -6.60 |
|  |  |  |  |  |  |  |  |
| **M-O** |  |  |  |  |  |  |  |
| **Mice** |  | **Rats** |  | **Rhesus** |  | **Human** |  |
| **Symbol** | **Zratio** | **Symbol** | **Zratio** | **Symbol** | **Zratio** | **Symbol** | **Zratio** |
| GADD45A | 10.04 | CDO1 | 7.06 | S100A9 | 10.24 | SLPI | 8.56 |
| LGALS3 | 8.68 | LTBP1 | 5.69 | PLA2G2A | 7.50 | GADD45A | 7.41 |
| CXCL13 | 8.04 | GADD45A | 5.47 | HMOX1 | 6.52 | TNFRSF12A | 6.72 |
| S100A8 | 7.51 | SPP1 | 4.92 | ALOX5AP | 6.26 | CCL2 | 6.36 |
| CDKN1A | 5.54 | AOX1 | 4.79 | STAT1 | 6.02 | THBS1 | 5.38 |
| LYZ | 5.02 | ADIPOQ | 4.08 | SNCA | 5.02 | NFIL3 | 5.06 |
| S100A9 | 4.47 | CDKN1A | 3.98 | THBS2 | 4.68 | CD63 | 4.73 |
| ITGAV | 4.28 | NFE2L1 | 3.71 | LYZ | 4.34 | SIRPA | 4.65 |
| C3 | 3.38 | MTA1 | 3.66 | SPP1 | 4.19 | LITAF | 4.25 |
| FCER1G | 3.16 | CTSS | 3.64 | CEBPD | 4.17 | PDE4B | 3.94 |
| EGF | 3.11 | LITAF | 3.6 | PLEC1 | 4.09 | HSPB1 | 3.70 |
| CYLD | 2.77 | ADA | 3.57 | LCP1 | 4.07 | HP | 3.32 |
| MCL1 | 2.77 | LGALS3 | 3.56 | NCL | 3.92 | DIAPH1 | 3.31 |
| CD74 | 2.72 | CCL5 | 3.47 | CD14 | 3.81 | DARC | 3.29 |
| GDNF | 2.72 | ALOX15 | 3.32 | CSF1R | 3.78 | KERA | 3.26 |
| IER3 | 2.64 | IL33 | 3.01 | DARC | 3.57 | FIGF | 3.25 |
| ALOX5AP | 2.54 | PLA2G2A | 2.74 | NFE2L1 | 3.54 | CSPG4 | 3.24 |
| CXCL10 | 2.45 | PTPN2 | 2.72 | THBS4 | 3.45 | SERPING1 | 2.90 |
| TLR4 | 2.41 | SIRPA | 2.72 | CIRBP | 3.28 | IRAK2 | 2.89 |
| FPR2 | 2.36 | APOE | 2.7 | FCGR2A | 3.11 | PLA2G2A | 2.82 |
| KERA | -2.69 | PDE4B | -2.18 | VNN1 | -2.27 | CD38 | -2.33 |
| C1QDC2 | -2.71 | ITGA1 | -2.19 | NR1D1 | -2.28 | FEM1A | -2.34 |
| ADIPOQ | -2.72 | CCND1 | -2.21 | F2 | -2.32 | ATG7 | -2.53 |
| ZFP36 | -2.77 | LUM | -2.38 | AHR | -2.38 | MAOB | -2.54 |
| NEDD9 | -2.86 | ABR | -2.63 | BCR | -2.51 | VEGFA | -2.55 |
| PRKCD | -2.91 | CEBPB | -2.68 | ZFP36 | -2.56 | LYZ | -2.57 |
| VEGFB | -2.97 | LGALS1 | -2.72 | PRKCB | -2.62 | SLC37A4 | -2.60 |
| SERPINF1 | -3.03 | MGLL | -2.75 | VEGFA | -2.62 | ITGB6 | -2.77 |
| PDGFB | -3.05 | CDC42 | -2.75 | DDIT3 | -2.67 | EPAS1 | -2.86 |
| FOS | -3.23 | SERPINF1 | -2.83 | LCN2 | -2.81 | CEBPB | -2.89 |
| BCL6 | -3.35 | GNAI2 | -2.84 | ACHE | -2.86 | ZP3 | -3.24 |
| SMAD3 | -3.58 | PRKCE | -2.9 | ATF3 | -3.01 | CD36 | -3.63 |
| PLA2G7 | -3.63 | CD47 | -2.93 | PLP1 | -3.03 | CD9 | -3.84 |
| HP | -3.66 | MAP2K2 | -3.04 | ADRB2 | -3.26 | ANGPT1 | -4.67 |
| MAOB | -3.71 | PARK7 | -3.1 | PDE4B | -3.33 | ACHE | -4.90 |
| KLF4 | -3.73 | SLC37A4 | -4.75 | TNFRSF9 | -4.11 | ZFP36 | -4.96 |
| NUPR1 | -3.88 | GSN | -5.02 | PLA2G10 | -4.70 | CEBPD | -5.19 |
| PRKG1 | -5.28 | SEMA7A | -5.2 | TFF2 | -6.75 | FOS | -5.36 |
| TIAM1 | -7.23 | KERA | -5.94 | EGR1 | -8.06 | HYAL1 | -6.11 |
| LEP | -8.24 | THBS4 | -15.5 | FOS | -15.09 | ACOT11 | -8.07 |
| **O-Y** |  |  |  |  |  |  |  |
| **Mice** |  | **Rats** |  | **Rhesus** |  | **Human** |  |
| **Symbol** | **Zratio** | **Symbol** | **Zratio** | **Symbol** | **Zratio** | **Symbol** | **Zratio** |
| LGALS3 | 8.33 | LTBP1 | 7.33 | PLA2G2A | 12.76 | SLPI | 11.84 |
| CXCL13 | 7.57 | CDO1 | 7.09 | S100A9 | 9.86 | GADD45A | 9.19 |
| GADD45A | 7.42 | GADD45A | 5.97 | TNFRSF12A | 7.47 | THBS4 | 6.58 |
| S100A8 | 7.41 | SPP1 | 5.36 | LYZ | 7.03 | CCL2 | 6.42 |
| LYZ | 5.69 | ADIPOQ | 5.12 | SNCA | 6.83 | THBS1 | 5.79 |
| CDKN1A | 4.61 | ADA | 4.79 | APOE | 6.43 | KERA | 5.56 |
| LEP | 4.50 | SLPI | 4.57 | DARC | 6.20 | TNFRSF12A | 5.22 |
| ADIPOQ | 4.12 | CD36 | 4.51 | ALOX5AP | 5.94 | PRNP | 5.13 |
| S100A9 | 3.96 | CDKN1A | 4.45 | CEBPD | 5.46 | CCL8 | 5.08 |
| TNFRSF12A | 3.91 | CCL5 | 4.25 | AOX1 | 5.05 | SIRPA | 4.99 |
| C3 | 3.74 | SIRPA | 4.07 | HMOX1 | 4.97 | HDAC4 | 4.81 |
| CYLD | 3.68 | CXCL12 | 3.57 | LCP1 | 4.58 | MFGE8 | 4.79 |
| EGF | 3.38 | CFH | 3.42 | STAT1 | 4.57 | PLA2G2A | 4.61 |
| FCER1G | 3.37 | NFE2L1 | 3.36 | DIAPH1 | 4.49 | CSPG4 | 4.40 |
| ITGAV | 3.13 | AOX1 | 3.33 | HP | 4.18 | GSN | 4.06 |
| CD74 | 3.10 | CTSS | 3.33 | CSF1R | 4.17 | SERPING1 | 3.96 |
| APOE | 2.96 | NFKB2 | 3.32 | ZFP36 | 3.95 | CXCL14 | 3.83 |
| APP | 2.84 | CD63 | 3.24 | STAT3 | 3.85 | NFIL3 | 3.51 |
| NFE2L1 | 2.70 | PLA2G7 | 3.18 | GSN | 3.81 | EGF | 3.38 |
| CIRBP | 2.65 | NR1H3 | 3.15 | FCAR | 3.75 | ANXA1 | 3.37 |
| NEDD9 | -2.30 | PLAU | -2.30 | ADRB2 | -2.14 | CXADR | -2.06 |
| SLC37A4 | -2.46 | HDAC4 | -2.34 | CCL21 | -2.35 | A2M | -2.08 |
| GNAI2 | -2.52 | MAPKAPK2 | -2.49 | AKT1 | -2.67 | PARP1 | -2.10 |
| KERA | -2.65 | MGLL | -2.49 | DDIT3 | -2.77 | EDG1 | -2.19 |
| KLF4 | -2.83 | LUM | -2.50 | PLP1 | -2.97 | ITGB6 | -2.31 |
| PRKG1 | -2.93 | SLC37A4 | -2.51 | IER3 | -3.03 | ETS1 | -2.37 |
| ZFP36 | -2.98 | GSN | -2.57 | EPHX2 | -3.12 | PPBP | -2.58 |
| PTX3 | -3.13 | FYN | -2.97 | PARK7 | -3.21 | CD9 | -2.73 |
| PON1 | -3.15 | CNTF | -3.13 | ACHE | -3.35 | LIAS | -2.76 |
| SERPINF1 | -3.27 | ASH1L | -3.24 | TNFRSF9 | -3.37 | IGF1 | -2.81 |
| PRKCD | -3.32 | ABCD2 | -3.33 | CXCR7 | -3.42 | SLC37A4 | -2.88 |
| CEBPB | -3.45 | ECM1 | -3.41 | ACOT11 | -3.55 | EPAS1 | -2.96 |
| ADRB2 | -3.59 | CD47 | -3.80 | ATF3 | -3.59 | CD38 | -2.98 |
| FOS | -3.63 | PRKCE | -3.88 | ANGPTL2 | -3.67 | ACHE | -3.37 |
| BCL6 | -3.71 | SERPINF1 | -4.34 | KERA | -3.69 | SNCA | -3.53 |
| JAK2 | -4.32 | SEMA7A | -4.35 | SLC37A4 | -4.08 | GJA1 | -3.70 |
| NR4A2 | -4.59 | CCND1 | -4.42 | PARP1 | -4.41 | ACOT11 | -3.78 |
| PLA2G7 | -4.69 | NUPR1 | -5.10 | CX3CR1 | -4.90 | CD36 | -3.78 |
| SMAD3 | -4.88 | FN1 | -5.17 | GADD45A | -5.36 | HYAL1 | -4.37 |
| TIAM1 | -5.14 | THBS4 | -5.29 | PDE4B | -6.61 | SPP1 | -5.07 |
