# Supplementary material for: Conserved and species-specific molecular denominators in mammalian skeletal muscle aging
Source: NPJ Aging Mech Dis. 2017 May 5;3:8. doi: 10.1038/s41514-017-0009-8 (PMC5460213; doi:10.1038/s41514-017-0009-8)
Supplement: Supplementary file 14 — Supplementary file [file 41514_2017_9_MOESM14_ESM.docx]

**Supplementary tables**

**Table S1.** Mouse, Rats, Rhesus monkeys and human oligonucleotides used for real-time RT-qPCR.

**Table S2**. Up- and down-regulated pathway interactions between the different species and ages.

**Table S3**. Z-ratio of all significant up- and down-regulated pathways significantly changed by age.

**Table S4**. Z-ratio of the top 20 up- and down-regulated inflammatory genes between the different species and ages.

**Table S5**. Complete list of common pathways between species.

**Supplementary figures**

**Fig. S1**. Muscle wet weight of soleus (SOL), plantaris (PL), gastrocnemius (GAS), tibialis (TB), extensor digitorum longus (EDL) of young (Y), middle-aged (M) and old (O) mice. Values are expressed as mean ± SEM. ^*^p < 0.05 versus young.

**Fig S2.** Ingenuity pathway analysis (IPA) of mitochondrial dysfunction of genes differentially expressed in skeletal muscle of **(A)** mice, **(B)** rats, rhesus monkeys **(C)** and **(D)** humans in response to age**.** Genes that are significantly up-regulated or down-regulated by age are shown in red and green respectively. Genes in grey did not exhibit significant changes with age. Young (Y), middle-aged (M) and old (O) mice.

**Fig S3.** Ingenuity pathway analysis (IPA) of mTOR signaling of genes differentially expressed in skeletal muscle of **(A)** mice, **(B)** rats, rhesus monkeys **(C)** and **(D)** humans in response to age**.** Genes that are significantly up-regulated or down-regulated by age are shown in red and green respectively. Genes in grey did not exhibit significant changes with age. Young (Y), middle-aged (M) and old (O) mice.

**Fig S4.** Ingenuity pathway analysis (IPA) of IGF-1 signaling of genes differentially expressed in skeletal muscle of **(A)** mice, **(B)** rats, rhesus monkeys **(C)** and **(D)** humans in response to age**.** Genes that are significantly up-regulated or down-regulated by age are shown in red and green respectively. Genes in grey did not exhibit significant changes with age. Young (Y), middle-aged (M) and old (O) mice.

**Fig. S5 (A-F)** Full size sequentially decorated blots of mitochondrial complexes in skeletal muscle of rats.

**Fig. S6 (A-F)** Full size sequentially decorated blots of mitochondrial complexes in skeletal muscle of rhesus monkeys.

**Fig. S7.** Full size in gel complex I activity stain in skeletal muscle of rhesus monkeys.

**Fig. S8 (A-E)** Full size sequentially decorated blots of mitochondrial complexes in skeletal muscle of humans.
